# Synthesis of new substituted 7,12-dihydro-6,12-methanodibenzo[*c,f*]azocine-5-carboxylic acids containing a tetracyclic tetrahydroisoquinoline core structure

**DOI:** 10.3762/bjoc.17.168

**Published:** 2021-10-07

**Authors:** Agnieszka Grajewska, Maria Chrzanowska, Wiktoria Adamska

**Affiliations:** 1 Faculty of Chemistry, Adam Mickiewicz University, ul. Uniwersytetu Poznańskiego 8, 61-614 Poznań, Poland

**Keywords:** amino acids, cyclization, multicomponent reactions, synthetic methods, tetrahydroisoquinoline

## Abstract

A convenient and simple protocol has been developed for the synthesis of a series of new tetracyclic tetrahydroisoquinoline derivatives, 7,12-dihydro-6,12-methanodibenzo[*c,f*]-azocine-5-carboxylic acids by three component Petasis reaction with the use of aminoacetaldehyde acetals bearing substituted benzyl groups as the amine components followed by Pomeranz–Fritsch double cyclization reaction. By applying this method, several acids have been prepared in satisfactory yields. An unprecedented chemical behavior of a Petasis reaction product in diluted HCl solution leading to the formation of a phenylglycine derivative has been observed and the mechanism explaining such reactivity has been proposed.

## Introduction

**Figure 1:**
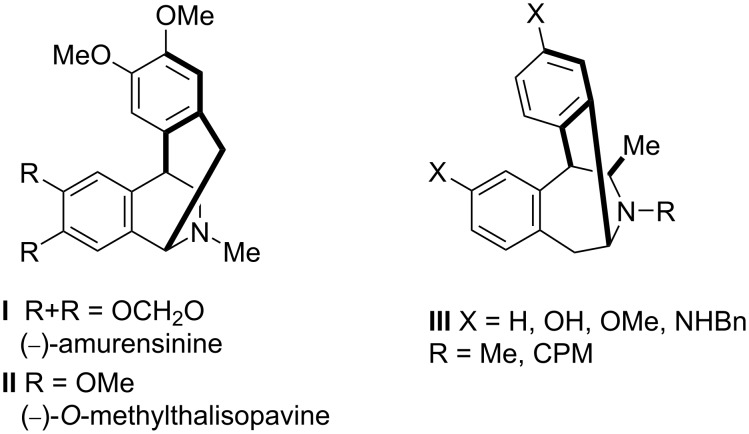
Natural isopavine alkaloids and synthetic derivatives of isopavine.

The tetrahydroisoquinoline moiety is the key structure in large number of natural and synthetic biologically important molecules. Among them, isoquinoline alkaloids constitute a large family of natural products exhibiting a huge range of structural diversity and biological activity [1–3]. Within this family several structural motifs can be identified, for instance, the isopavines, constrained alkaloids possessing the 1,2,3,4-tetrahydroisoquinoline skeleton incorporated into the double cyclized azabicyclo[3.2.2]nonane, isolated from *Papaveraceae* and *Ranunculaceae* species. Natural isopavines (e.g., amurensinine (**I**), and *O*-methylthalisopavine (**II**), Figure 1) as well as their synthetic analogues have attracted much attention [4–5] because of their interactions with the receptors in the central nervous system promising for the treatment of nervous system disorders, such as Parkinson’s and Alzheimer’s disease [6–7].

**Figure 2:**
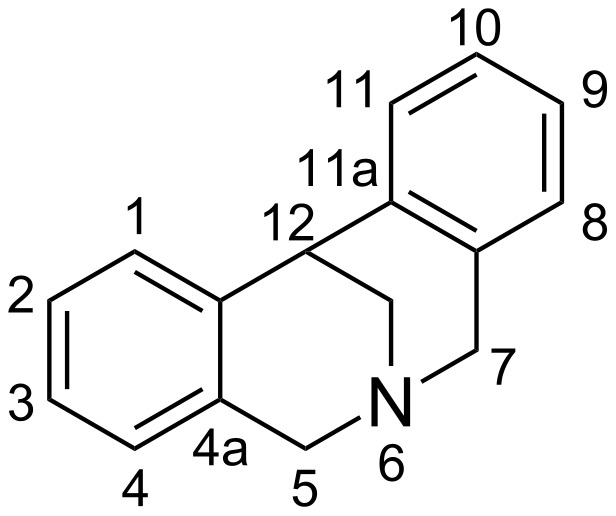
The structure and numbering of dihydromethanodibenzoazocine.

Hanessian and co-workers designed isopavines of type **III** (Figure 1) that act as morphinomimethics and bind strongly to human opioid receptors [8–9]. The synthesis of these compounds involved the stereocontrolled [1,2]-Stevens rearrangement of dihydromethanodibenzoazocines. Dihydromethanodibenzoazocines are example of strained, methylene-bridged tetrahydroisoquinolines (Figure 2) that have attracted much interest of scientists not only due to their resemblance to isopavines and thus their medicinal significance in the central nervous system but also as a building blocks for the synthesis of alkaloids [10–11].

The classical strategy for the synthesis of dihydromethanodibenzoazocines is based on the use of *N*,*N*-dibenzylaminoacetaldehydes [8–9] and less often α-dibenzylaminoketone [12–13] derivatives in the intramolecular Friedel–Crafts double cyclization reaction and *N*,*N*-dibenzylaminoacetaldehyde dialkyl acetals [12,14–16] in the Pomeranz–Fritsch-type double cyclization reaction. There is also one example of employing *p*-quinol acetates as substrates for the synthesis of these compounds [17]. Recently, a methodology for the synthesis of dihydromethanodibenzoazocines based on a combination of Friedel–Crafts and Pictet–Spengler reactions was proposed by Moshkin et al. [18].

**Scheme 1:**
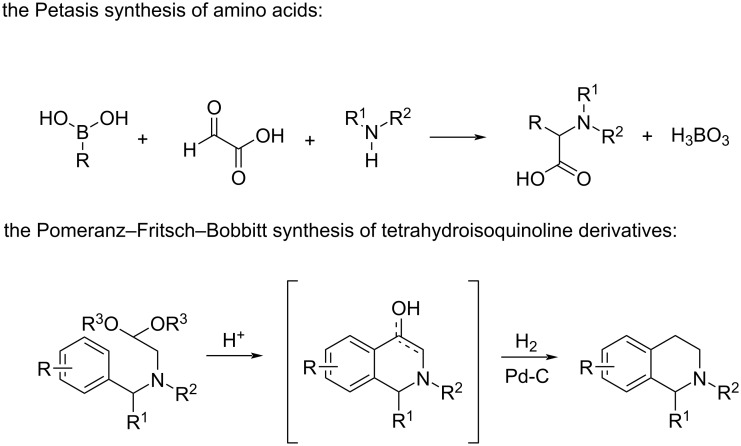
The Petasis reaction and the Pomeranz–Fritsch–Bobbitt cyclization.

We have developed a convenient method for the preparation of C-1-substituted tetrahydroisoquinoline derivatives by using the Petasis three-component reaction followed by the Pomeranz–Fritsch–Bobbitt cyclization. The Petasis reaction between boronic acids, carbonyl derivatives, and amines, leading to the formation of amino acids and the Pomeranz–Fritsch–Bobbitt cyclization of amino acetals, leading to the construction of C-1-functionalized tetrahydroisoquinoline rings, are presented in Scheme 1.

In the Petasis reaction we applied aminoacetaldehyde acetals as the amine component which enabled the preparation of the substrates for the Pomeranz–Fritsch–Bobbitt cyclization in one simple step. The Petasis reaction products were further transformed into racemic tetrahydroisoquinoline-1-carboxylic acids [19], simple isoquinoline alkaloids [20], and (+)-6,7-dimethoxy-1,2,3,4-tetrahydroisoquinoline-1-carboxylic acid [21].

**Scheme 2:**
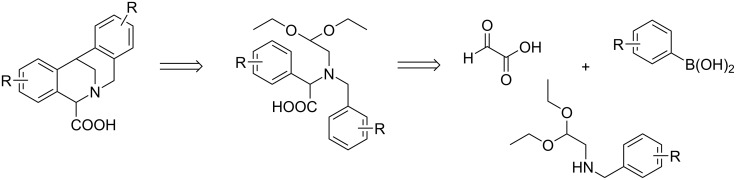
The synthesis of 7,12-dihydro-6,12-methanodibenzo[*c,f*]azocine-5-carboxylic acids via a combination of the Petasis reaction and the Pomeranz–Fritsch reaction – a retrosynthetic approach.

In this paper we report a straightforward synthesis of new strained tetracyclic tetrahydroisoquinoline derivatives, variously substituted 7,12-dihydro-6,12-methanodibenzo[*c,f*]azocine-5-carboxylic acids, via our modified method based on a combination of the Petasis reaction, in which we used aminoacetaldehyde acetals with substituted *N*-benzyl groups as the amine component and the Pomeranz–Fritsch double cyclization reaction (Scheme 2).

## Results and Discussion

**Scheme 3:**
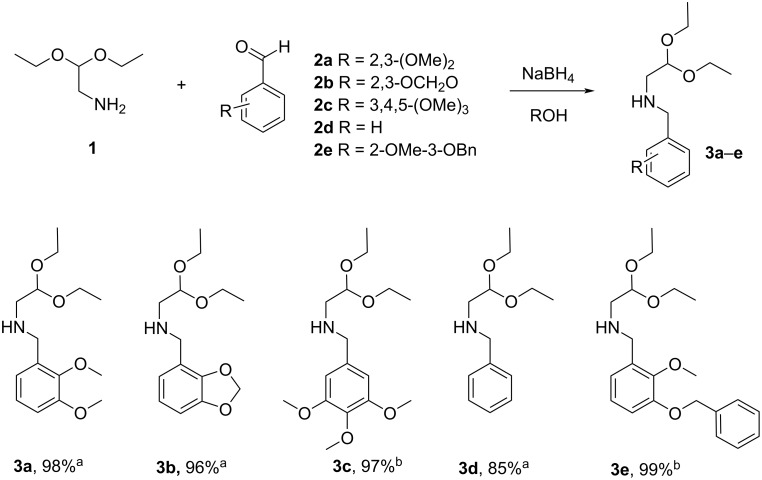
Synthesis of *N*-benzylated aminoacetaldehyde acetals **3a**–**e**. Conditions: a) reaction run in EtOH; b) reaction run in MeOH.

Our investigations commenced with the synthesis of *N*-benzylated aminoacetaldehyde acetals **3a**–**e**, the amine components for the Petasis reaction. The condensation of aminoacetaldehyde diethyl acetal **1** and 2,3-dimethoxybenzaldehyde (**2a**) was carried out at rt in anhydrous ethanol and the formed imine was then reduced *in situ* with sodium borohydride to give the product **3a** with 98% yield. The synthesis of further *N*-benzylated aminoacetaldehyde acetals **3b**–**e** was performed in these reaction conditions in EtOH or MeOH to provide the desired products with high overall yields of 85–99% as indicated in Scheme 3. The aminoacetals **3a**–**e** were suitable for use directly in the next step of the synthesis without further purification.

**Scheme 4:**
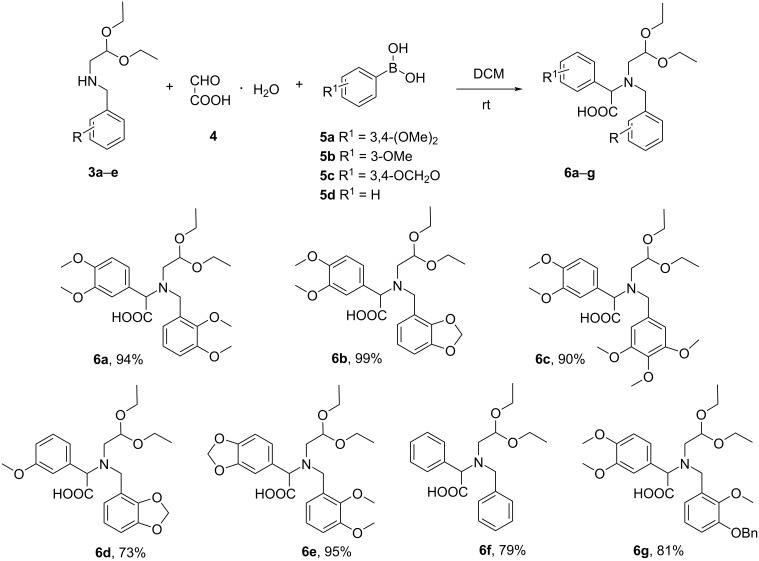
Synthesis of amino acids **6a**–**g**.

Then, the *N*-benzylated aminoacetals **3a**–**e** were subjected to the Petasis reaction with glyoxylic acid hydrate (**4**) and the appropriate boronic acids **5a**–**d** carried out in DCM at rt for 24 h to afford amino acids **6a**–**g** (Scheme 4).

The condensation of *N*-(2,3-dimethoxybenzyl)aminoacetaldehyde acetal (**3a**) with glyoxylic acid hydrate (**4**) and 3,4-dimethoxyphenylboronic acid (**5a**) afforded the Petasis reaction product **6a** in a high 94% yield.

**Scheme 5:**
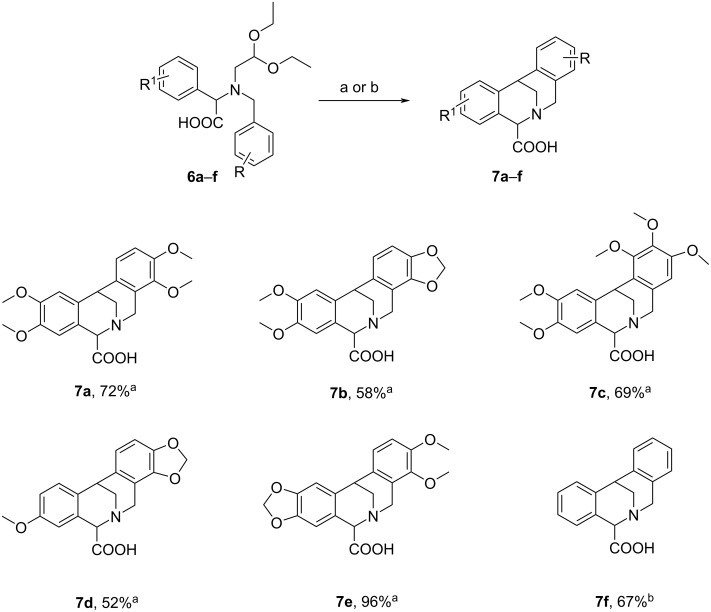
Synthesis of dihydromethanodibenzoazocine-5-carboxylic acids **7a**–**f**. Conditions: a) 20% HCl, rt, 24 h; b) 70% HClO_4_, rt, 12 h.

The double cyclization reaction was easily performed by treatment of **6a** with 20% HCl for 24 h to give dihydromethanodibenzoazocine-5-carboxylic acid (**7a**, Scheme 5) in 72% yield by extraction of the neutralized reaction mixture. Compound **7a** was obtained in two simple steps from **3a** with an overall yield of 67%.

Then the amino acid **6a** was subjected to cyclization under different acidic conditions (not shown). The reaction of **6a** with TiCl_4_ or SnCl_4_ in DCM at 0 °C led exclusively to the formation of **7a** (TLC analysis) but it was impossible to isolate the product in a pure form probably due to the formation of the salts.

When **6a** was treated with 70% HClO_4_ for 2 hours, the TLC analysis indicated the presence of **7a** as the main product together with unreacted substrate and other difficult to separate by-products. When the reaction time was prolonged to 24 h all the substrate was consumed but a mixture of unidentified products was formed.

Then, *N*-2,3-methylenedioxybenzylamino acid **6b** and *N*-3,4,5-trimethoxybenzylamino acid **6c** were prepared from boronic acid **5a**, glyoxylic acid hydrate (**4**) and aminoacetals **3b** and **3c**, in 99% and 90% yield, respectively (Scheme 4). Under the Pomeranz–Fritsch conditions compounds **6b** and **6c** were transformed into dihydromethanodibenzoazocine-5-carboxylic acids **7b** with a yield of 58% and **7c** with a yield of 69%. The overall yields for compounds **7b** and **7c** starting from **3b** and **3c**, were 57% and 62%, respectively (Scheme 5).

Next, 3-methoxyphenylboronic acid (**5b**) and 3,4-methylenedioxyphenylboronic acid (**5c**) reacted easily with aminoacetals **3b** and **3a**, respectively and glyoxylic acid hydrate (**4**), affording the corresponding arylglycine derivatives **6d** and **6e**, in satisfactory yields of 73% and 95%, respectively (Scheme 4). After cyclization in 20% HCl, compounds **6d** and **6e** were transformed into the desired dihydromethanodibenzoazocine-5-carboxylic acids **7d** and **7e** with yields of 52% and 96%, respectively. The overall yields of **7d** and **7e** (from **3d** and **3e**, respectively) were 37% and 91%, respectively (Scheme 5).

In the case of the amino acid **6f**, containing insufficiently activated phenyl rings without alkoxy substituents, prepared from phenylboronic acid (**5d**), glyoxylic acid hydrate (**4**) and an *N*-benzyl aminoacetal **3d** with good yield 79% (Scheme 4), it was necessary to perform the cyclization reaction using 70% HClO_4_ instead of 20% HCl. In these conditions, the desired product **7f** was obtained with 67% yield (53% overall yield from **3d**), while the reaction carried out in 20% HCl led to a mixture of products, among which **7f** was not detected (Scheme 5).

It should be also mentioned that the amino acid **6g** with a benzyloxy-substituted aromatic ring, obtained in the Petasis reaction of boronic acid **5a**, glyoxylic acid hydrate (**4**), and *O*-benzylvanilin-derived aminoacetal **3e** (Scheme 4), when treated with 20% HCl or 70% HClO_4_ gave a colored complex mixture of decomposition products, probably due to the hydrolysis of the benzyl ether.

**Scheme 6:**
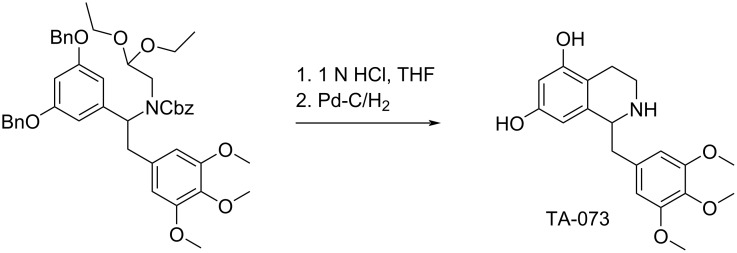
Synthesis of TA-073.

Iwakuma et al. [15] reported the synthesis of 1,2,3,4-tetrahydroisoquinoline derivative TA-073 using substituted *N*-acyl aminoacetal as the key intermediate which, depending on the reaction conditions, could be transformed either into TA-073 or the appropriate double cyclized product, isopavine or pavine (not shown) (Scheme 6).

**Scheme 7:**
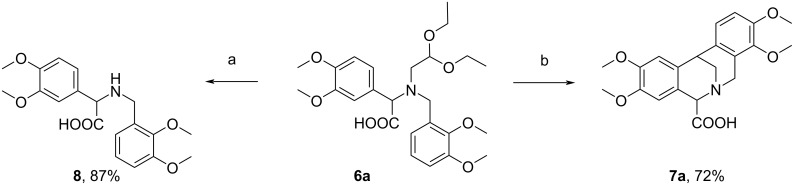
Reaction of **6a** with 4% aqueous HCl solution in THF and with 20% aqueous HCl solution. Conditions: a) 4% HCl (aq), THF, reflux; b) 20% HCl, rt.

Inspired by these results, we next investigated the possibility of obtaining monocyclized derivatives from the Petasis reaction products of type **6**. In this experiment the amino acid **6a** has been chosen as a model compound and it was treated with 4% aqueous HCl in THF at reflux [15]. Surprisingly, in these conditions we isolated the *N*-benzylated phenylglycine derivative **8** as the sole product with 87% yield (Scheme 7). When we decreased the concentration of HCl to 2% in the reaction with **6a**, the substrate was isolated unchanged even after prolonged reaction time.

**Scheme 8:**
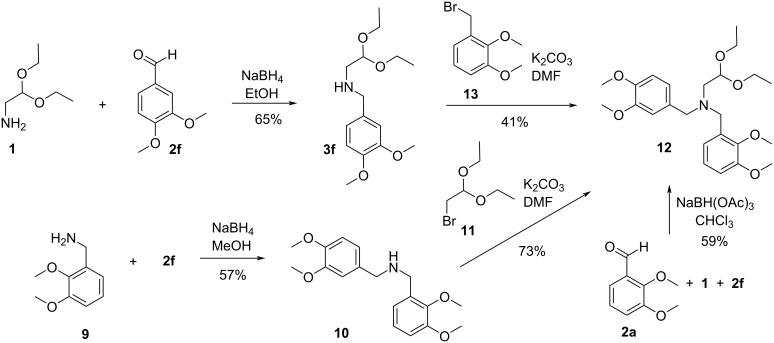
Three pathways of the synthesis of **12**, the decarboxylated analogue of **6a**.

In light of these results, we investigated the chemical behavior of compound **12**, the decarboxylated analogue of **6a**, under the reaction conditions that led to products **7a** or **8** starting from acid **6a**. The substrate for the synthesis of compound **12** was aminoacetal **3f**, obtained with moderate 49% yield through the condensation of aminoacetaldehyde diethyl acetal (**1**) and 3,4-dimethoxybenzaldehyde (**2f**) carried out in refluxing toluene using a Dean*–*Stark apparatus followed by the reduction with NaBH_4_ (not shown). When the condensation of acetal **1** and aldehyde **2f** and the subsequent reduction were carried out in EtOH at rt, according to our procedure applied for the synthesis of aminoacetals **3a**–**e**, the product **3f** was isolated with 65% yield (Scheme 8).

In the reaction of aminoacetal **3f** with 2,3-dimethoxybenzyl bromide (**13**) product **12** was formed, however, it was not pure enough to be used directly in the next step of the synthesis. Purification of the crude reaction mixture by column chromatography was difficult and resulted in a moderate yield of **12** (41%) due to insufficient differences in the *R*_f_ values of compound **12** and the by-product 2,3-dimethoxybenzyl alcohol (not shown), formed through the hydrolysis of **13** (Scheme 8).

In this situation *N*-(2,3-dimethoxybenzyl)veratrylamine **10** was chosen as the substrate for the synthesis of the decarboxylated derivative **12** and simultaneously **10** was the expected product of its reaction with 4% HCl. The amine **10** was obtained by reductive amination of veratral (**2f**) with 2,3-dimethoxybenzylamine (**9**) with 57% yield and subsequently alkylated with bromoacetaldehyde diethyl acetal (**11**) to give **12** with 73% yield.

Additionally, the aminoacetal **12** was also synthesized via another route involving a double reductive amination of the aldehydes **2a** and **2f** with aminoacetaldehyde acetal (**1**) with 59% yield.

**Scheme 9:**
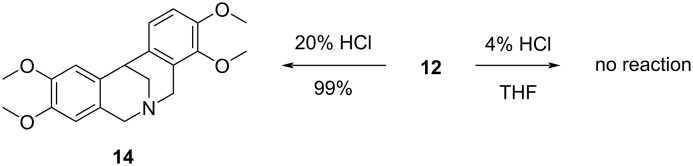
The chemical behavior of **12** in 4% aqueous HCl solution in THF and in 20% aqueous HCl solution.

The aminoacetal **12** was then treated with 4% HCl in THF at reflux but in contrast to the chemical behavior of amino acid **6a** in the same conditions (see Scheme 7), no reaction was observed. The cyclization reaction of **12** carried out in 20% HCl led, as expected, to the double-cyclized derivative **14** (Scheme 9).

**Scheme 10:**
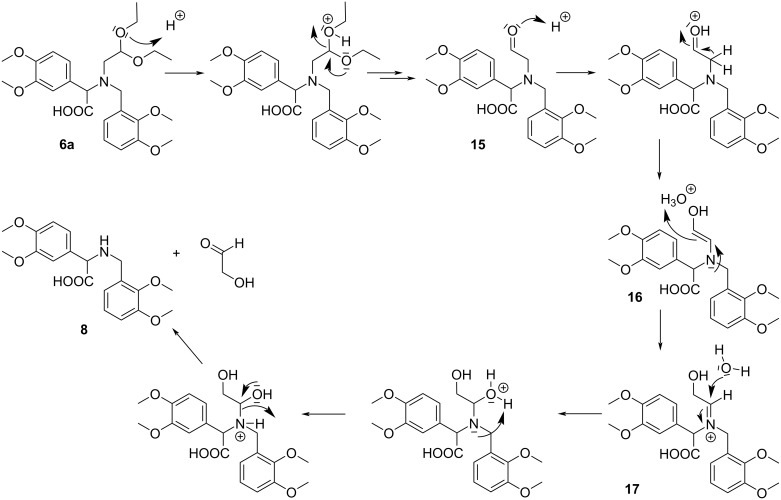
A plausible mechanism of the reaction of **6a** with 4% aqueous HCl solution in THF.

To explain the difference in the reactivity between **6a** and **12** we proposed a plausible mechanism for the reaction of **6a** with diluted HCl (Scheme 10). The mechanism consists of four major steps: the first step is an acid-catalyzed hydrolysis of the acetal function in **6a** to afford aldehyde **15**; the second step is the enolization of the aldehyde **15** to form the tautomeric compound **16**; the next step involves the formation of the iminium ion **17**, which is then hydrolyzed to compound **8** and hydroxyacetaldehyde in the last step.

Thus, the unexpected reactivity of **6a** with diluted HCl may be explained by the electron-withdrawing effect exerted by the adjacent COOH group which facilitates hydrolysis of the iminium ion **17**.

## Conlusion

In conclusion, a straightforward and efficient method for the synthesis of new tetrahydroisoquinoline derivatives, 7,12-dihydro-6,12-methanodibenzo[*c,f*]azocine-5-carboxylic acids **7a**–**f** has been developed. It is based on the Petasis reaction with the use of aminoacetaldehyde acetals bearing substituted benzyl groups as the amine components followed by Pomeranz–Fritsch double cyclization reaction. The presence of the carboxyl group in **7a**–**f** allows synthesis of new derivatives of these unexplored tetracyclic tetrahydroisoquinolines for biochemical studies. The unusual interesting behavior of Petasis reaction product **6a** in diluted HCl solution leading to phenylglycine derivative **8** has been detected and the mechanism explaining this reactivity has been proposed.

## Supporting Information

File 1 Experimental.

File 2 Copies of ^1^H NMR and ^13^C NMR spectra.

## References

[1] Scott J D, Williams R M (2002). Chem Rev.

[2] Bentley K W (2005). Nat Prod Rep.

[3] Bentley K W (1998). The Isoquinoline Alkaloids.

[4] Chrzanowska M, Rozwadowska M D (2004). Chem Rev.

[5] Chrzanowska M, Grajewska A, Rozwadowska M D (2016). Chem Rev.

[6] Weber E, Keana J, Barmettler P (1990). PCP Receptor ligands and the use thereof. International Patent Application.

[7] Childers W E, Abou-Gharbia M A (1990). 10,11-Dihydro-5-alkyl-12-substituted-10,5-(iminomethano)-5H-dibenzo[AD]cycloheptenes as Neuroprotectant Agents. U.S. Patent.

[8] Hanessian S, Mauduit M (2001). Angew Chem, Int Ed.

[9] Hanessian S, Parthasarathy S, Mauduit M, Payza K (2003). J Med Chem.

[10] Hanessian S, Mauduit M, Demont E, Talbot C (2002). Tetrahedron.

[11] Nomoto T, Nasui N, Takayama H (1984). J Chem Soc, Chem Commun.

[12] Suzuki T, Takamoto M, Okamoto T, Takayama H (1986). Chem Pharm Bull.

[13] Coşkun N, Buyukuysal L (1998). Heterocycles.

[14] Bobbitt J M, Shibuya S (1970). J Org Chem.

[15] Yamada K, Takeda M, Itoh N, Ohtsuka H, Tsunashima A, Iwakuma T (1982). Chem Pharm Bull.

[16] Mottinelli M, Leese M P, Potter B V L (2017). Beilstein J Org Chem.

[17] Hara H, Hoshino O, Umezawa B (1985). Chem Pharm Bull.

[18] Buev E M, Stepanov M A, Moshkin V S, Sosnovskikh V Y (2020). Org Lett.

[19] Chrzanowska M, Grajewska A, Meissner Z, Rozwadowska M D, Wiatrowska I (2012). Tetrahedron.

[20] Chrzanowska M, Grajewska A, Rozwadowska M D (2012). Heterocycles.

[21] Bułyszko I, Chrzanowska M, Grajewska A, Rozwadowska M D (2015). Eur J Org Chem.

