# Supplementary material for: Synthesis of new substituted 7,12-dihydro-6,12-methanodibenzo[c,f]azocine-5-carboxylic acids containing a tetracyclic tetrahydroisoquinoline core structure
Source: Beilstein J Org Chem. 2021 Oct 7;17:2511–9. doi: 10.3762/bjoc.17.168 (PMC8505899; doi:10.3762/bjoc.17.168)
Supplement: File 1 — Experimental. [file Beilstein_J_Org_Chem-17-2511-s001.pdf]

BEILSTEIN JOURNAL OF ORGANIC CHEMISTRY

# Supporting Information

for

# Synthesis of new substituted 7,12-dihydro-6,12-methanodibenzo[*c,f*]azocine-5-carboxylic acids containing a tetracyclic tetrahydroisoquinoline core structure

Agnieszka Grajewska, Maria Chrzanowska and Wiktoria Adamska

*Beilstein J. Org. Chem.* **2021,** *17,* 2511–2519. doi:10.3762/bjoc.17.168

# Experimental

License and Terms: This is a supporting information file under the terms of the Creative Commons Attribution License (https://creativecommons.org/licenses/by/4.0). Please note that the reuse, redistribution and reproduction in particular requires that the author(s) and source are credited and that individual graphics may be subject to special legal provisions.
The license is subject to the *Beilstein Journal of Organic Chemistry* terms and conditions: (https://www.beilstein-journals.org/bjoc/terms)

**General:** Melting points were determined on a Koffler block and are uncorrected. IR spectra were recorded on a Bruker FT-IR IFS 113V spectrophotometer or Jasco FT-IR 4600 spectrophotometer with ATR PRO ONE using a diamond crystal. $^{1}$H and $^{13}$C NMR spectra were recorded on a Bruker ASCEND 400 spectrometer. Chemical shifts are reported in parts per million (ppm). Spectra are referenced using an internal reference (tetramethylsilane or $CDCl_3$, DMSO-d6 residual solvent peak). Mass spectra (EI) were measured using an AMD402 spectrometer. High-resolution mass spectra (HRMS) were measured using an Impact HD (Bruker Daltonics) spectrometer. Merck DC-Alufolien Kieselgel $60_{254}$ were used for TLC and silica gel (100–200 mesh ASTM) for column chromatography. All reagents and solvents were purchased from commercial suppliers and used as received unless otherwise noted. *O*-Benzylvanilin was synthesized according to the literature procedure [1].

**2,3-Methylenedioxybenzaldehyde (2b)**

2,3-Dihydroxybenzaldehyde (319 mg, 2.31 mmol) and $CH_2I_2$ (897 mg, 0.27 mL, 3.35 mmol) were dissolved in anhydrous DMF (0.3 mL). Then, anhydrous $K_2CO_3$ (341 mg, 2.47 mmol) was added and the mixture was heated at 100 °C for 5 h under reflux condenser capped with a calcium chloride drying tube. After that time diethyl ether was added (5 mL) at room temperature, and the mixture was stirred for 10 min. The solvents were decanted and the residue was washed with fresh ether (3 × 5mL). The extracts were combined, washed with 10% NaOH (3 × 5mL), water (3 × 5mL), and dried over anhydrous $Na_2SO_4$. The solvent was evaporated and the residue was purified by column chromatography (hexane/ethyl acetate 96:4 v/v) to obtain **2b** as a yellowish solid (246 mg, 71%). The product was identical with commercial sample supplied by Sigma Aldrich.

**Preparation of aminoacetaldehyde acetals 3a–f – general procedure:**

Aminoacetaldehyde diethyl acetal (**1**, 1 equiv) and the aromatic aldehyde **2a–f** (1 equiv) were dissolved in anhydrous ethanol (0.7 mL per 1 mmol of substrates) or methanol (1.7 mL per 1 mmol of substrates) and the solution was stirred at room temperature for 48 h. After that time, the solution was cooled in an ice–water bath and $NaBH_4$ (1.0–1.5 equiv) was added portion-wise. The reaction mixture was stirred at room temperature for 18 h, then the solvent was evaporated under reduced pressure, and the residue was treated with water. After extraction with DCM, drying over $Na_2SO_4$, and evaporation of the solvent, products **3a–e** were obtained as a colorless to pale yellow oil. It was pure enough to use in the next step without further purification. An analytical sample was obtained by purification of the crude material via column chromatography on silica gel.

**N-(2,3-Dimethoxybenzyl)aminoacetaldehyde diethyl acetal (3a)**

The compound **3a** was synthesized according to the general procedure from aminoacetaldehyde diethyl acetal (**1**, 746 mg, 5.60 mmol), 2,3-dimethoxybenzaldehyde (**2a**, 931 mg, 5.60 mmol) in ethanol (3.92 mL) using $NaBH_4$ (221 mg, 5.84 mmol). The crude product **3a** obtained as a pale yellowish liquid (1555 mg, 98%) was pure enough to use in the next step without further purification. An analytical sample was purified by flash chromatography (100:0 to 97:3 v/v DCM/MeOH). The $^{1}H$ NMR spectrum of **3a** was identical with that in the literature [2].

**N-(2,3-Methylenedioxybenzyl)aminoacetaldehyde diethyl acetal (3b)**

The compound **3b** was synthesized according to the general procedure from aminoacetaldehyde diethyl acetal (**1**, 500 mg, 3.75 mmol), 2,3-methylenedioxybenzaldehyde (**2b**, 563 mg, 3.75 mmol) in ethanol (2.5 mL) using $NaBH_4$ (188 mg, 4.97 mmol). The crude product **3b** obtained as a pale yellowish liquid (961 mg, 96%) was pure enough to use in the next step without further purification. An analytical sample was purified by flash chromatography (100:0 to 97:3 v/v DCM/MeOH). $^{1}H$ NMR (400 MHz, $CDCl_3$): δ = 6.83 (s, 1H), 6.75 (s, 2H), 5.93 (s, 2H), 4.60 (t, *J* = 5.6 Hz, 1H), 3.71 (s, 2H), 3.72 – 3.65 (m, 2H), 3.57 – 3.49 (m, 2H), 2.72 (d, *J* = 5.6 Hz, 2H), 1.56 (s, broad, 1H), 1.21 (t, *J* = 7.1 Hz, 6H); $^{13}C$ NMR (101 MHz, $CDCl_3$): δ = 147.7, 146.5, 134.2, 121.2, 108.7, 108.1, 102.2, 100.9, 62.4, 53.7, 51.4, 15.4; IR (neat): $\tilde{\nu}$ = 2978, 2885, 1488, 1440, 1245, 1034 $cm^{-1}$; EI MS: m/z (%): 267 ($M^+$, 3), 164 (5), 151 (12), 135 (100), 103 (58); HRMS: m/z calcd for $C_{14}H_{21}NO_4$: 267.14706; found: 267.14793.

**N-(3,4,5-Trimethoxybenzyl)aminoacetaldehyde diethyl acetal (3c)**

The compound **3c** was synthesized according to the general procedure from aminoacetaldehyde diethyl acetal (**1**, 426 mg, 3.20 mmol), 3,4,5-trimethoxybenzaldehyde (**2c**, 628 mg, 3.20 mmol) in methanol (5.4 mL) using $NaBH_4$ (181 mg, 4.78 mmol). The crude product **3c** obtained as a colorless liquid (974 mg, 97%) was pure enough to use in the next step without further purification. An analytical sample was purified by flash chromatography (100:0 to 97:3 v/v DCM/MeOH). $^{1}H$ NMR (400 MHz, $CDCl_3$): δ = 6.56 (s, 2H), 4.63 (t, *J* = 5.5 Hz, 1H), 3.86 (2s, 6H), 3.83 (s, 3H), 3.75 (s, 2H), 3.74 – 3.66 (m, 2H), 3.58 – 3.50 (m, 2H), 2.76 (d, *J* = 5.5 Hz, 2H), 1.61 (s, broad, 1H), 1.21 (t, *J* = 7.1 Hz, 6H); $^{13}C$ NMR (101 MHz, $CDCl_3$): δ = 153.2, 136.8, 136.0, 104.8, 102.1, 62.3, 60.8, 56.0, 54.1, 51.5, 15.4; IR (neat): $\tilde{\nu}$ = 2973, 2896, 1589, 1505, 1456, 1235, 1123, 1056 $cm^{-1}$; EI MS: m/z (%): 313 ($M^+$, 10), 267 (11), 222 (14), 197 (23), 181 (100), 103 (46); HRMS: m/z calcd for $C_{16}H_{27}NO_5$: 313.18893; found: 313.18965.

**N-Benzylaminoacetaldehyde diethyl acetal (3d)**

The compound **3d** was synthesized according to the general procedure from aminoacetaldehyde diethyl acetal (**1**, 1.150 g, 8.63 mmol), benzaldehyde (**2d**, 916 mg, 8.63 mmol) in ethanol (6.0 mL) using $NaBH_4$ (326 mg, 8.59 mmol). The crude product **3d** obtained as a pale yellowish liquid (1638 mg, 85%) was pure enough to use in the next step without further purification. The $^{1}H$ NMR spectrum of **3d** was identical with that in the literature [3].

### *N*-(3-Benzyloxy-2-methoxybenzyl)aminoacetaldehyde diethyl acetal (3e)

The compound **3e** was synthesized according to the general procedure from aminoacetaldehyde diethyl acetal (**1**, 436 mg, 3.27 mmol), *O*-benzylvanilin (**2e**, 792 mg, 3.27 mmol) in methanol (5.6 mL) using $NaBH_4$ (180 mg, 4.76 mmol). The crude product **3e** obtained as a colorless liquid (1163 mg, 99%) was pure enough to use in the next step without further purification. An analytical sample was purified by flash chromatography (100:0 to 98:2 v/v DCM/MeOH). $^{1}H$ NMR (400 MHz, $CDCl_3$): δ = 7.50 – 7.45 (m, 2H), 7.42 – 7.31 (m, 3H), 7.05 (t, *J* = 7.9 Hz, 1H), 6.91 (ddd, *J* = 11.8, 7.9, 1.4 Hz, 2H), 5.06 (s, 2H), 4.58 (t, *J* = 5.6 Hz, 1H), 3.91 (s, 3H), 3.76 (s, 2H), 3.69 – 3.62 (m, 2H), 3.54 – 3.46 (m, 2H), 2.70 (d, *J* = 5.6 Hz, 2H), 1.80 (s, broad, 1H), 1.19 (t, *J* = 7.1 Hz, 6H); $^{13}C$ NMR (101 MHz, $CDCl_3$) δ = 152.7, 146.1, 137.9, 134.2, 128.4, 128.1, 127.9, 123.9, 121.6, 111.4, 102.3, 74.8, 62.2, 55.8, 51.5, 48.8, 15.3; IR (neat): $\tilde{\nu}$ = 2976, 2891, 1477, 1267, 1061 $cm^{-1}$; EI MS: m/z (%): 359 ($M^+$, 2), 313 (12), 227 (26), 195 (16), 181 (14), 162 (15), 91 (100); HRMS: m/z calcd for $C_{21}H_{29}NO_4$: 359.20966; found: 359.21025.

### *N*-(3,4-Dimethoxybenzyl)aminoacetaldehyde diethyl acetal (3f)

#### Method A

The compound **3f** was synthesized according to the general procedure from aminoacetaldehyde diethyl acetal (**1**, 253 mg, 1.90 mmol), 3,4-dimethoxybenzaldehyde (**2f**, 316 mg, 1.90 mmol) in ethanol (1.5 mL) using $NaBH_4$ (98 mg, 2.59 mmol). The crude product obtained as a colorless liquid (350 mg, 65%) was pure enough to use in the next step without further purification. The $^{1}H$ NMR spectrum of the product was identical with that in the literature [3].

**Method B**

Aminoacetaldehyde diethyl acetal (**1**, 716 mg, 5.38 mmol) and 3,4-dimethoxybenzaldehyde (**2f**, 907 mg, 5.46 mmol) were dissolved in toluene (4.5 mL), and the solution was heated at reflux (Dean–Stark trap) for 7 h. After that time, toluene was evaporated, and the residue was dissolved in 96% ethanol (4.5 mL). The resulting solution was cooled in an ice–water bath and then treated with $NaBH_4$ (292 mg, 7.72 mmol). The reaction mixture was stirred at room temperature for 18 h, then the solvent was evaporated under reduced pressure, and the residue was treated with water. After extraction with DCM, drying over $Na_2SO_4$, and evaporation of the solvent, product **3f** was obtained as a pale yellow oil. It was purified by column chromatography (100:0 to 90:10 v/v DCM/MeOH and 70:30 to 15:85 v/v hexane/EtOAc) to give pure **3f** (748 mg, 49% yield) as a colorless oil.

**Synthesis of Petasis reaction products 6a–g – general procedure:**

In a manner similar to that described in [4], a suspension of glyoxylic acid monohydrate (**4**) and boronic acid **5a–d** in a 1:1 molar ratio in DCM (5 mL per 1 mmol of substrates) was stirred in a round-bottomed flask at room temperature for 5–10 min, and then aminoacetaldehyde acetal **3a–e** (1 molar equiv) was added. The clear solution was stirred at room temperature for 24–48 h. After that time, the inorganic solid precipitate was removed by filtration, washed with DCM and the solvent was removed under reduced pressure. The crude product was purified by column chromatography.

***N*-(2,3-Dimethoxybenzyl)-*N*-(2,2-diethoxyethyl)-3,4-dimethoxyphenylglycine (6a)**

The compound **6a** was synthesized according to the general procedure from 3,4-dimethoxyphenylboronic acid (**5a**), glyoxylic acid monohydrate (**4**), and *N*-(2,3-dimethoxybenzyl)aminoacetaldehyde diethyl acetal (**3a**) on a 5.49 mmol scale (reaction time: 24 h). The crude product was purified by column chromatography (99:1 to 92:8 v/v DCM/MeOH) to give pure **6a** (2.454 g, 94%) as a colorless foam. $^{1}H$ NMR (400 MHz, $CDCl_3$): δ = 7.04 (dd, *J* = 8.1, 7.7 Hz, 1H), 6.93 – 6.86 (m, 5H), 4.80 (s, 1H), 4.36 (t, *J* = 5.2 Hz, 1H), 3.92 – 3.89 (overlapped: s, 3H and d, 1H), 3.89 (s, 3H), 3.89 (s, 3H), 3.88 (s, 3H), 3.64 – 3.55 (m, 3H), 3.48 – 3.42 (m, 1H), 3.41 – 3.31 (m, 1H), 2.74 (dd, *J* = 14.0, 5.5 Hz, 1H), 2.56 (dd, *J* = 14.0, 4.8 Hz, 1H), 1.18 (t, *J* = 7.0 Hz, 3H), 1.15 (t, *J* = 7.1 Hz, 3H); $^{13}C$ NMR (101 MHz, $CDCl_3$): δ = 172.5, 152.7, 149.2, 148.8, 147.9, 129.7, 125.4, 124.3, 123.0, 122.9, 113.4, 112.7, 111.0, 101.5, 69.7, 63.4, 63.2, 61.0, 56.0, 55.9, 55.8, 53.1, 52.4, 15.2, 15.1; IR (KBr): $\tilde{\nu}$ = 2936, 2837, 1717, 1629, 1516, 1265, 1146 $cm^{-1}$; EI MS: m/z (%): 433 (1) $[M–C_2H_5O]^+$, 356 (8), 315

(7), 238 (16), 194 (20), 166 (27), 151 (100), 136 (37), 101 (26); HRMS (ESI): m/z calcd for $C_{25}H_{35}NO_8$ $[M+H]^+$: 478.2441; found: 478.2452.

***N*-(2,3-Methylenedioxybenzyl)-*N*-(2,2-diethoxyethyl)-3,4-dimethoxyphenylglycine (6b)**

The compound **6b** was synthesized according to the general procedure from 3,4-dimethoxyphenylboronic acid (**5a**), glyoxylic acid monohydrate (**4**), and *N*-(2,3-methylenedioxybenzyl)aminoacetaldehyde diethyl acetal (**3b**) on a 0.68 mmol scale (reaction time: 24 h). The crude product was purified by column chromatography (99.6:0.4 to 96:4 v/v DCM/MeOH) to give pure **6b** (311mg, 99%) as a colorless foam. $^1$H NMR (400 MHz, $CDCl_3$): δ = 6.89 – 6.80 (m, 4H), 6.78 – 6.76 (m, 2H), 6.01 (s, 2H), 4.73 (s, 1H), 4.51 (dd, *J* = 6.2, 4.2 Hz, 1H), 3.90 – 3.87 (overlapped: d, 1H; s, 3H; s, 3H), 3.87 (s, 3H), 3.72 – 3.60 (m, 2H), 3.56 (d, *J* = 13.6 Hz, 1H), 3.51 – 3.42 (m, 2H), 2.72 (dd, *J* = 13.9, 6.2 Hz, 1H), 2.45 (dd, *J* = 13.9, 4.1 Hz, 1H), 1.22 (t, *J* = 7.0 Hz, 3H), 1.20 (t, *J* = 7.1 Hz, 3H); $^{13}$C NMR (101 MHz, $CDCl_3$): δ = 172.7, 149.1, 148.7, 147.6, 146.0, 125.1, 122.9, 122.8, 122.2, 118.8, 113.2, 110.9, 108.6, 101.3, 100.9, 68.8, 63.4, 63.1, 55.9, 55.9, 53.7, 52.0, 15.3, 15.1; IR (neat): $\tilde{\nu}$ = 2973, 2889, 1603, 1508, 1238, 1030 cm$^{-1}$; EI MS: m/z (%): 416 (7) $[M-C_2H_5O]^+$, 372 (11), 299 (11), 223 (18), 209 (12), 135 (100); HRMS (ESI): m/z calcd for $C_{24}H_{31}NO_8$ $[M+H]^+$: 462.2128; found: 462.2115.

***N*-(3,4,5-Trimethoxybenzyl)-*N*-(2,2-diethoxyethyl)-3,4-dimethoxyphenylglycine (6c)**

The compound **6c** was synthesized according to the general procedure from 3,4-dimethoxyphenylboronic acid (**5a**), glyoxylic acid monohydrate (**4**), and *N*-(3,4,5-trimethoxybenzyl)aminoacetaldehyde diethyl acetal (**3c**) on a 1.08 mmol scale (reaction time: 24 h). The crude product was purified by column chromatography (99.4:0.6 to 90:10 v/v DCM/MeOH) to give pure **6c** (493 mg, 90% yield) as a colorless oil. $^1$H NMR (400 MHz, $CDCl_3$): δ = 6.85 (s, 2H), 6.82 (s, 1H), 6.60 (s, 2H), 4.73 (s, 1H), 4.44 (dd, *J* = 6.0, 3.8 Hz, 1H), 3.95 (d, *J* = 13.7 Hz, 1H), 3.87 (s, 3H), 3.85 (s, broad, 9H), 3.84 (s, 3H), 3.75 – 3.69 (m, 1H), 3.65 – 3.55 (m, 2H), 3.49 – 3.42 (m, 2H), 2.84 (dd, *J* = 13.9, 6.3 Hz, 1H), 2.58 (dd, *J* = 13.9, 3.6 Hz, 1H), 1.23 (t, *J* = 7.0 Hz, 3H), 1.17 (t, *J* = 7.0 Hz, 3H); $^{13}$C NMR (101 MHz, $CDCl_3$): δ = 172.6, 153.5, 149.2, 148.9, 137.6, 132.5, 125.8, 122.6, 113.0, 110.9, 105.7, 101.0, 69.0, 63.7, 63.1, 60.8, 57.5, 56.1, 55.9, 55.9, 53.3, 15.2, 15.2. IR (neat): $\tilde{\nu}$ = 2968, 1717, 1589, 1507, 1238, 1123 cm$^{-1}$; EI MS: m/z (%): 461 (2), 417 (6), 239 (3), 181 (100); Elemental analysis: calcd (%) for $C_{26}H_{37}NO_9 \cdot 1/5\ H_2O$: C: 61.09, H: 7.37, N: 2.74; found: C: 61.13, H: 7.61, N: 2.78.

***N*-(2,3-Methylenedioxybenzyl)-*N*-(2,2-diethoxyethyl)-3-methoxyphenylglycine (6d)**

The compound **6d** was synthesized according to the general procedure from 3-methoxyphenylboronic acid (**5b**), glyoxylic acid monohydrate (**4**), and *N*-(2,3-methylenedioxybenzyl)aminoacetaldehyde diethyl acetal (**3b**) on a 1 mmol scale (reaction time: 24 h). The crude product was purified by column chromatography (100:0 to 90:10 v/v DCM/MeOH) to give pure **6d** (315 mg, 73% yield) as a colorless foam. $^{1}$H NMR (400 MHz, $CDCl_3$): δ = 7.31 – 7.27 (m, 1H), 6.91 – 6.88 (m, 4H), 6.82 (dd, *J* = 8.0, 1.2 Hz, 1H), 6.78 (d, *J* = 7.9 Hz, 1H), 5.97 (d, *J* = 0.8 Hz, 2H), 4.75 (s, 1H), 4.44 (dd, *J* = 6.2, 3.9 Hz, 1H), 3.93 (d, *J* = 13.5 Hz, 1H), 3.80 (s, 3H), 3.76 – 3.70 (m, 1H), 3.66 (d, *J* = 13.6 Hz, 1H), 3.61 – 3.54 (m, 1H), 3.51 – 3.40 (m, 2H), 2.86 (dd, *J* = 14.0, 6.3 Hz, 1H), 2.59 (dd, *J* = 14.0, 3.8 Hz, 1H), 1.24 (t, *J* = 7.0 Hz, 3H), 1.18 (t, *J* = 7.0 Hz, 3H); $^{13}$C NMR (101 MHz, $CDCl_3$): δ = 172.6, 159.6, 148.1, 147.4, 134.9, 130.6, 129.5, 122.5, 122.2, 115.8, 113.8, 109.2, 108.3, 101.1, 101.1, 68.6, 63.7, 63.0, 57.1, 55.2, 53.2, 15.2, 15.2; IR (neat): $\tilde{\nu}$ = 2974, 2893, 1716, 1601, 1488, 1247, 1036 $cm^{-1}$; EI MS: m/z (%): 387 (1), 341 (29), 269 (59), 148 (16), 135 (100); Elemental analysis: calcd (%) for $C_{23}H_{29}NO_7 \cdot H_2O$: C: 61.46, H: 6.95, N: 3.12; found: C: 61.24, H: 7.08, N: 2.96.

***N*-(2,3-Dimethoxybenzyl)-*N*-(2,2-diethoxyethyl)-3,4-methylenedioxyphenylglycine (6e)**

The compound **6e** was synthesized according to the general procedure from 3,4-methylenedioxyphenylboronic acid (**5c**), glyoxylic acid monohydrate (**4**), and *N*-(2,3-dimethoxybenzyl)aminoacetaldehyde diethyl acetal (**3a**) on a 1.26 mmol scale (reaction time: 24 h). The crude product was purified by column chromatography (100:0 to 90:10 v/v DCM/MeOH) to give pure **6e** (552 mg, 95%) as a colorless foam. $^{1}$H NMR (400 MHz, $CDCl_3$): δ = 7.04 (t, *J* = 7.9 Hz, 1H), 6.91 (dd, *J* = 8.2, 1.3 Hz, 1H), 6.86 (dd, *J* = 7.7, 1.1 Hz, 1H), 6.84 – 6.82 (m, 3H), 5.98 (dd, *J* = 4.4, 1.4 Hz, 2H), 4.79 (s, 1H), 4.35 (t, *J* = 5.2 Hz, 1H), 3.90 (s, 3H), 3.89 – 3.86 (overlapped: d, 1H and s, 3H), 3.66 – 3.58 (m, 1H), 3.57 – 3.51 (m, 2H), 3.48 – 3.40 (m, 1H), 3.38 – 3.31 (m, 1H), 2.71 (dd, *J* = 14.0, 5.4 Hz, 1H), 2.56 (dd, *J* = 14.0, 4.9 Hz, 1H), 1.18 (t, *J* = 7.2 Hz, 3H), 1.15 (t, *J* = 7.1 Hz, 3H); $^{13}$C NMR (101 MHz, $CDCl_3$): δ = 172.6, 152.7, 147.9, 147.7, 147.7, 129.9, 126.6, 124.3, 124.2, 122.9, 112.6, 110.5, 108.2, 101.7, 101.2, 69.6, 63.5, 63.0, 61.0, 55.8, 53.0, 52.4, 15.2, 15.1; IR (neat): $\tilde{\nu}$ = 2973, 2889, 1712, 1611, 1483, 1230, 1035 $cm^{-1}$; EI MS: m/z (%): 416 (10) $[M–C_2H_5O]^+$, 371 (23), 340 (55), 299 (35), 270 (43), 209 (13), 179 (28), 151 (100), 136 (80); Elemental analysis: calcd (%) for $C_{24}H_{31}NO_8$: C: 62.46, H: 6.77, N: 3.04; found: C: 62.41, H: 6.61, N: 3.22.

***N*-Benzyl-*N*-(2,2-diethoxyethyl)phenylglycine (6f)**

The compound **6f** was synthesized according to the general procedure from phenylboronic acid (**5d**), glyoxylic acid monohydrate (**4**), and *N*-benzylaminoacetaldehyde diethyl acetal (**3d**) on a

2.38 mmol scale (reaction time: 48 h). The crude product was purified by column chromatography (100:0 to 97:3 v/v DCM/MeOH) to give pure **6f** (672 mg, 79%) as a colorless oil. $^{1}$H NMR (400 MHz, $CDCl_3$): δ = 7.41 – 7.33 (m, 8H), 7.32 – 7.30 (m, 2H), 4.78 (s, 1H), 4.43 (dd, *J* = 6.4, 3.7 Hz, 1H), 4.04 (d, *J* = 13.6 Hz, 1H), 3.75 – 3.69 (m, 1H), 3.65 (d, *J* = 13.7 Hz, 1H), 3.61 – 3.55 (m, 1H), 3.50 – 3.40 (m, 2H), 2.85 (dd, *J* = 13.9, 6.5 Hz, 1H), 2.51 (dd, *J* = 13.9, 3.7 Hz, 1H), 1.25 (t, *J* = 7.0 Hz, 3H), 1.18 (t, *J* = 7.0 Hz, 3H); $^{13}$C NMR (101 MHz, $CDCl_3$): δ = 172.5, 136.9, 133.1, 130.1, 129.1, 128.9, 128.6, 128.5, 128.1, 101.0, 69.0, 63.8, 63.2, 57.5, 53.6, 15.3, 15.2; IR (neat): $\tilde{\nu}$ = 2974, 2891, 1718, 1625, 1372, 1120, 1055 $cm^{-1}$; EI MS: m/z (%): 357 ($M^+$, 1), 312 (25), 254 (19), 194 (8), 117 (18), 102 (30), 91 (100); HRMS (ESI): m/z calcd for $C_{21}H_{27}NO_4$ $[M+H]^+$: 358.2018; found: 358.2018.

***N*-(3-Benzyloxy-2-methoxybenzyl)-*N*-(2,2-diethoxyethyl)-3,4-dimethoxyphenylglycine (6g)**

The compound **6g** was synthesized according to the general procedure from 3,4-dimethoxyphenylboronic acid (**5a**), glyoxylic acid monohydrate (**4**), and *N*-3-benzyloxy-2-methoxybenzylaminoacetaldehyde diethyl acetal (**3e**) on a 1.10 mmol scale (reaction time: 48 h). The crude product was purified by column chromatography (99.5:0.5 to 96:5 v/v DCM/MeOH) to give pure **6g** (493 mg, 81%) as a colorless oil. $^{1}$H NMR (400 MHz, $CDCl_3$): δ = 7.36 – 7.30 (m, 5H), 7.07 (t, *J* = 7.9 Hz, 1H), 6.93 (d, *J* = 7.9 Hz, 2H), 6.81 – 6.75 (m, 3H), 5.14 (d, *J* = 11.1 Hz, 1H), 4.97 (d, *J* = 11.1 Hz, 1H), 4.70 (s, 1H), 4.31 (dd, *J* = 5.7, 4.7 Hz, 1H), 3.91 (s, 3H), 3.86 (s, 3H), 3.75 (s, 3H), 3.65 – 3.50 (m, 4H), 3.41 – 3.29 (m, 2H), 2.69 (dd, *J* = 14.0, 5.9 Hz, 1H), 2.47 (dd, *J* = 14.0, 4.4 Hz, 1H), 1.15 (t, *J* = 7.1 Hz, 3H), 1.14 (t, *J* = 7.0 Hz, 3H); $^{13}$C NMR (101 MHz, $CDCl_3$): δ = 172.3, 152.7, 149.0, 148.6, 146.1, 136.9, 130.4, 128.8, 128.3, 128.2, 125.3, 124.4, 123.0, 122.3, 113.2, 112.3, 110.8, 101.1, 75.0, 69.5, 63.3, 63.2, 55.8, 55.8, 55.7, 53.1, 51.9, 15.1, 15.0; IR (neat): $\tilde{\nu}$ = 2934, 1714, 1594, 1515, 1257, 1059 $cm^{-1}$; EI MS: m/z (%): 509 (5), 463 (27), 356 (45), 300 (69), 280 (16), 254 (47), 227 (17), 178 (22), 166 (18), 151 (35), 91 (100); HRMS (ESI): m/z calcd for $C_{31}H_{39}NO_8$ $[M+H]^+$: 554.2754, found: 554.2758.

**Preparation of 7,12-dihydro-6,12-methanodibenzo[*c,f*]azocine-5-carboxylic acids 7a–e**

**General procedure:**

Compounds **6a–e** were dissolved in 20% hydrochloric acid (10 mL per 1 mmol), and the resulting solution was stirred at room temperature for 24 h. After that time 10% aqueous NaOH was added dropwise until the solution was neutral to litmus paper. The solution was extracted three times with a mixture of $CHCl_3$ and iPrOH 3:1 (v/v). The combined organic extracts were dried over $Na_2SO_4$ and concentrated in vacuo to afford sufficiently pure products **7a–e**. An analytical sample for characterization was prepared by chromatographic purification or recrystallization.

**2,3,8,9-Tetramethoxy-7,12-dihydro-6,12-methanodibenzo[*c,f*]azocine-5-carboxylic acid (7a)**

The compound **7a** was synthesized according to the general procedure from *N*-(2,3-dimethoxybenzyl-*N*-(2,2-diethoxyethyl)-3,4-dimethoxyphenylglycine (**6a**, 525 mg, 1.1 mmol). The crude compound (423 mg, quant.) was purified by flash chromatography (99:1 to 94:6 v/v DCM/MeOH) to give **7a** (305 mg, 72%), white solid.

M.p. 207 – 210 °C (dec.); $^{1}$H NMR (401 MHz, $CDCl_3$): δ = 7.39 (s, 1H), 6.98 (d, *J* = 8.4 Hz, 1H), 6.79 (d, *J* = 8.5 Hz, 1H), 6.69 (s, 1H), 4.91 (d, *J* = 17.9 Hz, 1H), 4.61 (s, 1H), 4.30 (d, *J* = 17.9 Hz, 1H), 4.01 – 3.94 (m, 1H), 3.87 (s, 3H), 3.83 (s, broad, 4H), 3.80 (s, 3H), 3.79 (s, 3H), 3.63 (d, *J* = 12.8 Hz, 1H). $^{13}$C NMR (101 MHz, $CDCl_3$): δ = 170.7, 150.8, 148.5, 147.9, 145.6, 130.7, 130.4, 122.2, 122.0, 119.5, 112.1, 111.4, 109.2, 68.6, 60.1, 56.0, 56.0, 55.8, 53.0, 47.3, 35.3. IR (KBr): $\tilde{\nu}$ = 3428, 2927, 1640, 1608, 1514, 1494, 1274 cm$^{-1}$; EI MS: m/z (%): 385 ($M^+$, 2), 341 (44), 312 (46), 297 (17), 190 (100); HRMS: m/z calcd for $C_{21}H_{23}NO_6$: 385.15253; found: 385.15263.

**2,3-Dimethoxy-8,9-methylenedioxy-7,12-dihydro-6,12-methanodibenzo[*c,f*]azocine-5-carboxylic acid (7b)**

The compound **7b** was synthesized according to the general procedure from *N*-(2,3-methylenedioxybenzyl)-*N*-(2,2-diethoxyethyl)-3,4-dimethoxyphenylglycine (**6b**, 321 mg, 0.695 mmol). The crude compound (211 mg, 82%) was purified by flash chromatography (99:1 to 96:4 v/v DCM/MeOH) to give **7b** (149 mg, 58%), white solid. M.p. 193 – 195 °C (dec.); $^{1}$H NMR (400 MHz, DMSO-d6): δ = 7.08 (s, 1H), 7.05 (s, 1H), 6.92 (s, 1H), 6.56 (s, 1H), 5.92 (s, 1H), 5.85 (s, 1H), 4.49 (s, 1H), 4.02 (d, *J* = 17.5 Hz, 1H), 3.76 (s, 3H), 3.68 (s, 1H), 3.64 (s, 3H), 3.56 (d, *J* = 13.0 Hz, 1H), 3.26 (d, *J* = 12.8 Hz, 2H); $^{13}$C NMR (101 MHz, DMSO-d6): δ

= 171.1, 147.8, 146.8, 145.6, 145.6, 132.9, 130.8, 124.4, 121.8, 111.2, 110.7, 107.8, 106.0, 100.5, 66.9, 56.3, 55.7, 55.6, 46.6, 34.3; IR (neat): $\tilde{\nu}$ = 3316, 2993, 1639, 1509, 1351, 1230, 1031 $cm^{-1}$; EI MS: m/z (%): 340 (3), 338 (17), 324 (100), 310 (21), 295 (21), 204 (26); HRMS (ESI): m/z calcd for $[C_{20}H_{19}NO_6+H]^+$: 370.129; found: 370.1287.

**2,3,9,10,11-Pentamethoxy-7,12-dihydro-6,12-methanodibenzo[*c*,*f*]azocine-5-carboxylic acid (7c)**

The compound **7c** was synthesized according to the general procedure from *N*-(3,4,5-trimethoxybenzyl)-*N*-(2,2-diethoxyethyl)-3,4-dimethoxyphenylglycine (**6c**, 198 mg, 0.39 mmol). The crude compound (141 mg, 87%) was purified by flash chromatography (99:1 to 96:4 v/v DCM/MeOH) to give **7c** (112 mg, 69%), white solid. M.p. 167–170 °C; $^1H$ NMR (400 MHz, $CDCl_3$): δ = 7.41 (s, 1H), 6.99 (s, 1H), 6.33 (s, 1H), 5.05 (d, *J* = 17.3 Hz, 1H), 4.74 (s, 1H), 4.26 (s, 1H), 4.21 (d, *J* = 17.5 Hz, 1H), 4.03 – 4.07 (m, 4H), 3.87 (s, 3H), 3.83 (s, 3H), 3.81 (s, 3H), 3.77 (s, 3H), 3.59 (d, *J* = 12.2 Hz, 1H); $^{13}C$ NMR (101 MHz, $CDCl_3$): δ = 170.5, 152.9, 149.8, 148.4, 147.8, 140.8, 129.9, 123.3, 123.1, 119.3, 111.2, 110.2, 104.6, 68.7, 61.0, 60.7, 56.0, 56.0, 55.9, 55.8, 47.8, 29.1. IR (neat): $\tilde{\nu}$ = 3353, 2940, 1612, 1516, 1496, 1460, 1347, 1115 $cm^{-1}$; EI MS: m/z (%): 414 (5), 370 (100), 356 (40), 190 (57); HRMS (ESI): m/z calcd for $[C_{22}H_{26}NO_7+H]^+$: 416.1709; found: 416.1703.

**3-Methoxy-8,9-methylenedioxy-7,12-dihydro-6,12-methanodibenzo[*c*,*f*]azocine-5-carboxylic acid (7d)**

The compound **7d** was synthesized according to the general procedure from *N*-(2,3-methylenedioxybenzyl-*N*-(2,2-diethoxyethyl)-3-methoxyphenylglycine (**6d**, 100 mg, 0.232 mmol). The crude compound (58 mg, 73%) was digested with DCM for 10 min. to give pure **7d** (41 mg, 52%), white solid. M.p. 200–205 °C (dec.); $^1H$ NMR (300 MHz, DMSO-d6): δ = 7.33 (d, *J* = 8.4 Hz, 1H), 6.95 (s, 1H), 6.89 (d, *J* = 2.4 Hz, 1H), 6.74 (dd, *J* = 8.3, 2.6 Hz, 1H), 6.55 (s, 1H), 5.91 (d, *J* = 0.8 Hz, 1H), 5.84 (d, *J* = 0.8 Hz, 1H), 4.54 (s, 1H), 4.45 (d, *J* = 17.5 Hz, 1H), 4.00 (d, *J* = 17.5 Hz, 1H), 3.70 (s, 1H), 3.66 (s, 3H), 3.56 (d, *J* = 13.0 Hz, 2H), 3.25 (d, *J* = 11.6 Hz, 2H); $^{13}C$ NMR (76 MHz, DMSO-d6): δ = 171.1, 157.1, 145.6, 145.6, 133.2, 132.9, 131.6, 128.1, 124.5, 112.8, 112.7, 107.6, 106.0, 100.5, 67.3, 56.4, 55.0, 46.6, 34.1; IR (neat): $\tilde{\nu}$ = 2974, 2516, 1611, 1503, 1484, 1461, 1360, 1243, 1029 $cm^{-1}$; EI MS: m/z (%): 339 ($M^+$, 2), 294 (67), 266 (100), 223 (8), 160 (35); HRMS (ESI): m/z calcd for $[C_{19}H_{17}NO_5+H]^+$: 340.1185; found: 340.1177.

**2,3-Methylenedioxy-8,9-dimethoxy-7,12-dihydro-6,12-methanodibenzo[*c,f*]azocine-5-carboxylic acid (7e)**

The compound **7e** was synthesized according to the general procedure from *N*-(2,3-dimethoxybenzyl)-*N*-(2,2-diethoxyethyl)-3,4-methylenedioxyphenylglycine (**6e**, 152 mg, 0.33 mmol). The crude compound was recrystallized from 96% EtOH to give **7e** (114 mg, 96%), white solid. M.p. 167 °C (dec.); $^{1}$H NMR (400 MHz, DMSO-d6): δ = 7.13 (d, *J* = 8.4 Hz, 1H), 7.02 (s, 1H), 6.86 (d, *J* = 8.4 Hz, 1H), 6.81 (s, 1H), 5.94 (s, 1H), 5.89 (s, 1H), 4.72 (s, 1H), 4.50 (d, *J* = 18.1 Hz, 1H), 4.19 (d, *J* = 18.1 Hz, 1H), 3.78 (s, 1H), 3.73 (s, 3H), 3.68 (s, 3H), 3.63 (d, *J* = 12.5 Hz, 1H), 3.27 (d, *J* = 12.1 Hz, 1H); $^{13}$C NMR (101 MHz, DMSO-d6): δ = 170.9, 150.2, 146.3, 145.4, 144.8, 133.7, 132.3, 124.1, 122.6, 122.1, 111.7, 107.6, 107.3, 100.8, 66.7, 59.4, 55.7, 52.4, 46.1, 33.9; IR (neat): $\tilde{\nu}$ = 3011, 2827, 1646, 1481, 1242, 1034 cm$^{-1}$; EI MS: m/z (%): 369 (3), 354 (2), 324 (2), 296 (75), 281 (32), 266 (16), 238 (10), 174 (100), 152 (17); HRMS: m/z calcd for $C_{20}H_{19}NO_6$: 369.12125; found: 369.12265.

**7,12-Dihydro-6,12-methanodibenzo[*c,f*]azocine-5-carboxylic acid (7f)**

*N*-Benzyl-*N*-(2,2-diethoxyethyl)-phenylglycine (**6f**, 199 mg, 0.357 mmol) was dissolved in 70% perchloric acid (1.61 mL) and the resulting solution was stirred at room temperature for 12 h. After that time, the mixture was neutralized with 10% sodium hydroxide. The solution was extracted four times with a mixture of $CHCl_3$ and iPrOH 3:1 (v/v). The combined organic extracts were dried over $Na_2SO_4$ and concentrated in vacuo to afford the crude product (125 mg, 85%) which was purified by flash chromatography (98:2 to 96:4 v/v DCM/MeOH) to give **7f** (99 mg, 67%), white solid. M.p. 150 °C (dec.); $^{1}$H NMR (400 MHz, $CDCl_3$): δ = 7.76 (d, *J* = 7.4 Hz, 1H), 7.28 – 7.24 (m, 2H), 7.12 (dq, *J* = 15.1, 7.6 Hz, 4H), 6.94 (d, *J* = 7.2 Hz, 1H), 5.07 (d, *J* = 17.4 Hz, 1H), 4.91 (s, 1H), 4.31 (d, *J* = 17.4 Hz, 1H), 4.16 (d, *J* = 12.3 Hz, 1H), 4.01 (s, 1H), 3.71 (d, *J* = 12.3 Hz, 1H); $^{13}$C NMR (101 MHz, $CDCl_3$): δ = 170.3, 137.5, 137.2, 129.0, 127.9, 127.8, 127.7, 127.5, 127.3, 127.3, 127.0, 126.9, 126.6, 68.9, 55.8, 47.0, 36.4; IR (neat): $\tilde{\nu}$ = 3369, 1642, 1337, 1080 cm$^{-1}$; EI MS: m/z (%): 266 [$(M+1)^+$, 2], 265 ($M^+$, 2), 220 (100), 192 (30), 178 (33), 129 (17); HRMS (ESI): m/z calcd for $[C_{17}H_{15}NO_2+H]^+$: 266.1182; found: 266.1182.

***N*-(2,3-Dimethoxybenzyl)-3,4-dimethoxyphenylglycine (8)**

*N*-(2,3-Dimethoxybenzyl)-*N*-(2,2-diethoxyethyl)-3,4-dimethoxyphenylglycine (**6a**, 210 mg, 0.44 mmol) was dissolved in THF (3.2 mL). Then, 0.44 mL of a 4% aqueous HCl solution was added and the reaction mixture was heated under reflux for 1.5 h. After that time water (5 mL) was added at rt and the mixture was extracted with diethyl ether (3 × 5 mL). The aqueous phase

was neutralized by the slow addition of 10% NaOH and it was extracted three times with mixture of $CHCl_3$ and iPrOH 3:1 (v/v). The combined organic extracts were dried over $Na_2SO_4$ and concentrated in vacuo to afford sufficiently pure **8** (138 mg, 87%). An analytical sample for characterization was prepared by chromatographic purification (9:1 to 6:4 v/v hexane/ethyl acetate). M.p. 134-136 °C; $^1$H NMR (403 MHz, DMSO-d6): δ = 7.05 (dd, *J* = 9.2, 6.2 Hz, 1H), 7.00 – 6.98 (m, 3H), 6.91 (s, broad, 2H), 4.19 (s, 1H), 3.79 (s, 3H), 3.76 (s, 2H), 3.74 (s, 3H), 3.72 (s, 3H), 3.67 (s, 3H); $^{13}$C NMR (75 MHz, DMSO-d6): δ = 171.0, 152.2, 148.5, 148.4, 146.8, 130.2, 129.8, 123.8, 121.4, 120.6, 112.4, 111.5, 111.4, 64.6, 60.2, 55.7, 55.5, 55.4, 44.2; IR (neat): $\tilde{\nu}$ = 2940, 1606, 1520, 1261, 1151, 1023 cm$^{-1}$; EI MS: m/z (%): 315 (46), 165 (100), 150 (56), 105 (10), 91 (33); HRMS (ESI): m/z calcd for $[C_{19}H_{23}NO_6+H]^+$: 362.1603; found: 362.1611.

**_N_-(2,3-Dimethoxybenzyl)-3,4-dimethoxybenzylamine (10)**

3,4-Dimethoxybenzaldehyde (**2f**, 219 mg, 1.32 mmol) and 2,3-dimethoxybenzylamine (**9**, 221 mg, 1.32 mmol) were dissolved in methanol (1.5 mL) and the solution was stirred at room temperature for 24 h. After that time, the solution was cooled in an ice–water bath and $NaBH_4$ (50 mg, 1.32 mmol) was added portion-wise. The reaction mixture was stirred at room temperature for 18 h, then the solvent was evaporated under reduced pressure and the residue was treated with water. After extraction with DCM, drying over $Na_2SO_4$, and evaporation of the solvent, the crude product was obtained as a pale yellow oil. It was purified by column chromatography (100:0 to 95:5 v/v DCM/MeOH) to afford pure **10** (240 mg, 57%) as a colorless oil. $^1$H NMR (400 MHz, $CDCl_3$): δ = 7.03 (t, *J* = 7.9 Hz, 1H), 6.96 – 6.90 (m, 2H), 6.86 (dd, *J* = 8.1, 1.5 Hz, 2H), 6.81 (d, *J* = 8.2 Hz, 1H), 3.89 (s, 3H), 3.87 (s, 3H), 3.87 (s, 3H), 3.84 (s, 5H), 3.74 (s, 2H), 2.44 (s, broad, 1H); $^{13}$C NMR (101 MHz, $CDCl_3$): δ = 152.6, 148.9, 148.0, 147.4, 133.4, 132.4, 123.9, 121.9, 120.3, 111.5, 111.5, 110.9, 60.7, 55.9, 55.8, 55.7, 52.6, 47.9; IR (neat): $\tilde{\nu}$ = 3333, 2934, 2832, 1586, 1512, 1462, 1226 cm$^{-1}$; EI MS: m/z (%): 317 ($M^+$, 5), 166 (49), 151 (100), 137 (29), 121 (24), 91 (9); HRMS (ESI): m/z calcd for $C_{18}H_{23}NO_4$ $[M+H]^+$: 318.1705; found: 318.1700.

**_N_-(2,3-Dimethoxybenzyl)-_N_-(3,4-dimethoxybenzyl)aminoacetaldehyde diethyl acetal (12)**

**Method A**

*N*-(3,4-Dimethoxybenzyl)aminoacetaldehyde diethyl acetal (**3f**, 349 mg, 1.23 mmol) and 2,3-dimethoxybenzyl bromide (**13**, 296 mg, 1.28 mmol) were dissolved in anhydrous DMF (2 mL). Then anhydrous $K_2CO_3$ (225 mg, 1.63 mmol) was added and the mixture was heated at reflux

for 2 h under a reflux condenser capped with a calcium chloride drying tube. After that time water (15 mL) was added at rt and the mixture was extracted with diethyl ether (4 × 10 mL). The combined organic extracts were dried over $Na_2SO_4$ and concentrated in vacuo. The residue was purified by column chromatography (100:0 to 95:5 v/v DCM/MeOH) to deposit pure **12** (219 mg, 41%) as a colorless oil.

**Method B**

*N*-(2,3-Dimethoxybenzyl)veratrylamine (**10**, 149 mg, 0.47 mmol) and bromoacetaldehyde diethyl acetal (**11**, 138 mg, 0.70 mmol) were dissolved in anhydrous DMF (0.5 mL). Then, $K_2CO_3$ (97 mg, 0.70 mmol) and KI (20 mg, 0.12 mmol) were added and the mixture was heated at 90 °C for 12 h under a reflux condenser capped with a calcium chloride drying tube. After that time water (5 mL) was added at rt and the mixture was extracted with diethyl ether (4 x 10 mL). The combined organic extracts were dried over $Na_2SO_4$ and concentrated in vacuo. The residue was purified by column chromatography (100:0 to 100:0.3 v/v DCM/MeOH) to deposit pure **12** (150 mg, 73% yield) as a colorless oil.

**Method C**

2,2-Diethoxyethylamine (**1**, 422 mg, 3.17 mmol) and 2,3-dimethoxybenzaldehyde (**2a**, 527 mg, 3.17 mmol) were dissolved in $CHCl_3$ (15 mL). Then to the solution $NaBH(OAc)_3$ (1028 mg, 4.85 mmol) was added portion-wise. After stirring for 2.5 h at rt, veratraldehyde (**2f**, 527 mg, 3.17 mmol) dissolved in $CHCl_3$ (1 mL) was introduced followed by $NaBH(OAc)_3$ (1028 mg, 4.85 mmol). After stirring for 12 h at rt, the reaction was quenched with a saturated aqueous solution of $NaHCO_3$ and the aqueous layer was extracted with DCM (3 × 25 mL). The combined organic extracts were washed with water (25 mL) and dried over $Na_2SO_4$, filtered, and evaporated in vacuo to give a pale yellow oil. The crude product was purified by column chromatography (100:0 to 95:5 v/v DCM/MeOH) to give pure **12** (811 mg, 59%) as a colorless oil.

$^1$H NMR (400 MHz, $CDCl_3$): δ = 7.18 – 7.13 (m, 1H), 7.03 (t, *J* = 7.9 Hz, 1H), 6.97 (d, *J* = 1.5 Hz, 1H), 6.88 (dd, *J* = 8.2, 1.5 Hz, 1H), 6.82 – 6.78 (m, 2H), 4.62 (t, *J* = 5.2 Hz, 1H), 3.87 (s, 3H), 3.86 (s, 3H), 3.86 (s, 3H), 3.79 (s, 3H), 3.74 (s, 2H), 3.63 (s, 2H), 3.61 – 3.55 (m, 2H), 3.50 – 3.42 (m, 2H), 2.66 (d, *J* = 5.2 Hz, 2H), 1.17 (t, *J* = 7.0 Hz, 6H); $^{13}$C NMR (101 MHz, $CDCl_3$): δ = 152.6, 148.7, 147.8, 147.6, 133.5, 132.5, 123.6, 122.2, 120.8, 111.9, 110.7, 102.1, 61.7, 60.6, 58.9, 56.1, 55.9, 55.7, 55.7, 52.4, 15.3; IR (neat): $\tilde{\nu}$ = 2971, 2832, 1587, 1512, 1476,

1229, 1129 cm$^{-1}$; EI MS: m/z (%): 433 ($M^+$, 1), 388 (3), 329 (100), 150 (24); HRMS (ESI): m/z calcd for $C_{24}H_{35}NO_6$ $[M+H]^+$: 434.2542; found: 434.2556.

**2,3,8,9-Tetramethoxy-7,12-dihydro-5*H*-6,12-methanodibenzo[*c,f*]azocine (14)**

*N*-(2,3-Dimethoxybenzyl)-*N*-(3,4-dimethoxybenzyl)aminoacetaldehyde diethyl acetal (**12**, 249 mg, 0,57 mmol) was dissolved in HCl (20%, 6 mL) and the mixture was stirred at room temperature for 24 h. After that time aqueous 20% NaOH solution was added to adjust the pH to alkaline and the mixture was extracted with DCM (3 × 10 mL). The combined organic extracts were dried over $Na_2SO_4$ and concentrated in vacuo. The crude product **14** (194 mg, 99%) was obtained as a pale yellow oil. An analytical sample was purified by flash chromatography (100:0.2 to 100:0.4 v/v DCM/MeOH). $^1$H NMR (400 MHz, $CDCl_3$): δ = 6.95 (d, *J* = 8.3 Hz, 1H), 6.72 (d, *J* = 8.3 Hz, 1H), 6.70 (s, 1H), 6.49 (s, 1H), 4.56 (d, *J* = 6.2 Hz, 1H), 4.51 (d, *J* = 7.1 Hz, 1H), 4.03 (d, *J* = 18.5 Hz, 1H), 3.87 – 3.83 (overlapped: s, 3H and d, 1H), 3.80 – 3.79 (overlapped: 3s, 9H), 3.59 (s, 1H), 3.36 (d, *J* = 12.5 Hz, 1H), 3.30 (d, *J* = 12.7 Hz, 1H); $^{13}$C NMR (101 MHz, $CDCl_3$): δ = 150.3, 147.5, 147.4, 145.5, 134.0, 132.6, 127.5, 125.4, 122.0, 110.7, 110.3, 108.9, 59.8, 57.2, 56.0, 55.8, 55.7, 53.4, 49.1, 35.0; IR (neat): $\tilde{\nu}$ = 2931, 2832, 1488, 1269, 1222, 1125, 1063 cm$^{-1}$; EI MS: m/z (%): 341 ($M^+$, 65), 340 (100), 339 (73), 323 (7), 267 (2), 151 (4); HRMS (ESI): m/z calcd for $C_{20}H_{23}NO_4$ $[M+H]^+$: 342.1705; found: 342.1699.

## References


[1] Radix, S.; Barret, R. *Tetrahedron* **2007**, *63*, 12379–12387.

[2] Lesèche, B.; Gilbert, J.; Viel, C. *J. Heterocycl. Chem.* **1981**, *18*, 143–153.

[3] Adeyemi, Ch. M.; Conibear, A. C.; Mutorwa, M. K.; Nokalipa, I. C.; Isaacsc, M.; Mnkandhla, D.; Hoppe, H. C.; Lobb, K. A.; Klein, R.; Kaye, P. T. *Bioorg. Chem.* **2020**, *101*, 103947.

[4] Bułyszko, I.; Chrzanowska, M.; Grajewska, A.; Rozwadowska, M. D. *Eur. J. Org. Chem.* **2015**, 383−388.
