# Supplementary material for: Synthesis of new substituted 7,12-dihydro-6,12-methanodibenzo[c,f]azocine-5-carboxylic acids containing a tetracyclic tetrahydroisoquinoline core structure
Source: Beilstein J Org Chem. 2021 Oct 7;17:2511–9. doi: 10.3762/bjoc.17.168 (PMC8505899; doi:10.3762/bjoc.17.168)

BEILSTEIN JOURNAL OF ORGANIC CHEMISTRY

Supporting Information

for

Synthesis of new substituted 7,12-dihydro-6,12-methanodibenzo[*c,f*]azocine-5-carboxylic acids containing a tetracyclic tetrahydroisoquinoline core structure

Agnieszka Grajewska, Maria Chrzanowska and Wiktoria Adamska

Beilstein J. Org. Chem. **2021,** *17,* 2511–2519. doi:10.3762/bjoc.17.168

Copies of ^{1}H NMR and ^{13}C NMR spectra

License and Terms: This is a supporting information file under the terms of the Creative Commons Attribution License (https://creativecommons.org/licenses/by/4.0). Please note that the reuse, redistribution and reproduction in particular requires that the author(s) and source are credited and that individual graphics may be subject to special legal provisions.
The license is subject to the *Beilstein Journal of Organic Chemistry* terms and conditions: (https://www.beilstein-journals.org/bjoc/terms)

Table of contents

^1H NMR and ^{13}C NMR of *N*-(2,3-methylenedioxybenzyl)aminoacetaldehyde diethyl acetal (**3b**) S2-S3

^1H NMR and ^{13}C NMR of *N*-(3,4,5-trimethoxybenzyl)aminoacetaldehyde diethyl acetal (**3c**) S4-S5

^1H NMR and ^{13}C NMR of *N*-(3-benzyloxy-2-methoxybenzyl)aminoacetaldehyde diethyl acetal (**3e**) S6-S7

^1H NMR and ^{13}C NMR of *N*-(2,3-dimethoxybenzyl)-*N*-(2,2-diethoxyethyl)-3,4-dimethoxyphenylglycine (**6a**) S8-S9

^1H NMR and ^{13}C NMR of *N*-(2,3-methylenedioxybenzyl)-*N*-(2,2-diethoxyethyl)-3,4-dimethoxyphenylglycine (**6b**) S10-S11

^1H NMR and ^{13}C NMR of *N*-(3,4,5-trimethoxybenzyl)-*N*-(2,2-diethoxyethyl)-3,4-dimethoxyphenylglycine (**6c**) S12-S13

^1H NMR and ^{13}C NMR of *N*-(2,3-methylenedioxybenzyl)-*N*-(2,2-diethoxyethyl)-3-methoxyphenylglycine (**6d**) S14-S15

^1H NMR and ^{13}C NMR of *N*-(2,3-dimethoxybenzyl)-*N*-(2,2-diethoxyethyl)-3,4-methylenedioxyphenylglycine (**6e**) S16-S17

^1H NMR and ^{13}C NMR of *N*-benzyl-*N*-(2,2-diethoxyethyl)phenylglycine (**6f**) S18-S19

^1H NMR and ^{13}C NMR of *N*-(3-benzyloxy-2-methoxybenzyl)-*N*-(2,2-diethoxyethyl)-3,4-dimethoxyphenylglycine (**6g**) S20-S21

^1H NMR and ^{13}C NMR of 2,3,8,9-tetramethoxy-7,12-dihydro-6,12-methanodibenzo[*c*,*f*]azocine-5-carboxylic acid (**7a**) S22-S23

^1H NMR and ^{13}C NMR of 2,3-dimethoxy-8,9-methylenedioxy-7,12-dihydro-6,12-methanodibenzo[*c*,*f*]azocine-5-carboxylic acid (**7b**) S24-S25

^1H NMR and ^{13}C NMR of 2,3,9,10,11-pentamethoxy-7,12-dihydro-6,12-methanodibenzo[*c*,*f*]azocine-5-carboxylic acid (**7c**) S26-S27

^1H NMR and ^{13}C NMR of 3-methoxy-8,9-methylenedioxy-7,12-dihydro-6,12-methanodibenzo[*c*,*f*]azocine-5-carboxylic acid (**7d**) S28-S29

^1H NMR and ^{13}C NMR of 2,3-methylenedioxy-8,9-dimethoxy-7,12-dihydro-6,12-methanodibenzo[*c*,*f*]azocine-5-carboxylic acid (**7e**) S30-S31

^1H NMR and ^{13}C NMR of 7,12-dihydro-6,12-methanodibenzo[*c*,*f*]azocine-5-carboxylic acid (**7f**) S32-S33

^1H NMR and ^{13}C NMR of *N*-(2,3-dimethoxybenzyl)-3,4-dimethoxyphenylglycine (**8**) S34-S35

^1H NMR and ^{13}C NMR of *N*-(2,3-dimethoxybenzyl)-3,4-dimethoxybenzylamine (**10**) S36-S37

^1H NMR and ^{13}C NMR of *N*-(2,3-dimethoxybenzyl)-*N*-(3,4-dimethoxybenzyl)aminoacetaldehyde diethyl acetal (**12**) S38-S39

^1H NMR and ^{13}C NMR of 2,3,8,9-tetramethoxy-7,12-dihydro-5*H*-6,12-methanodibenzo[*c*,*f*]azocine (**14**) S40-S41

^{1}H NMR of *N*-(2,3-methylenedioxybenzyl)aminoacetaldehyde diethyl acetal **(3b)**

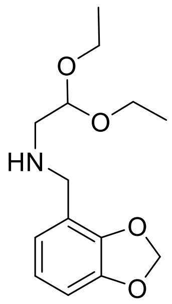

3b

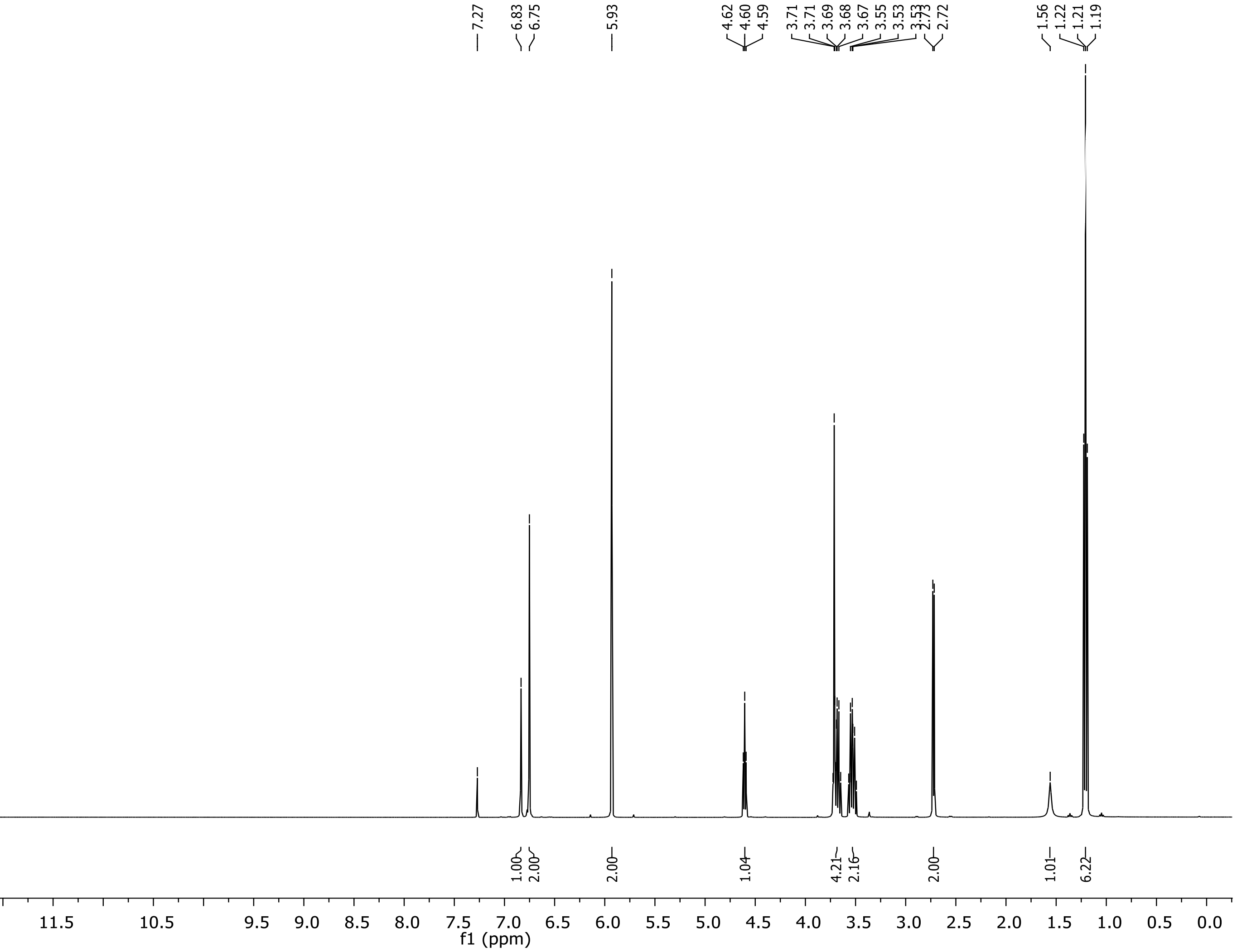

^{13}C NMR of *N*-(2,3-methylenedioxybenzyl)aminoacetaldehyde diethyl acetal **(3b)**

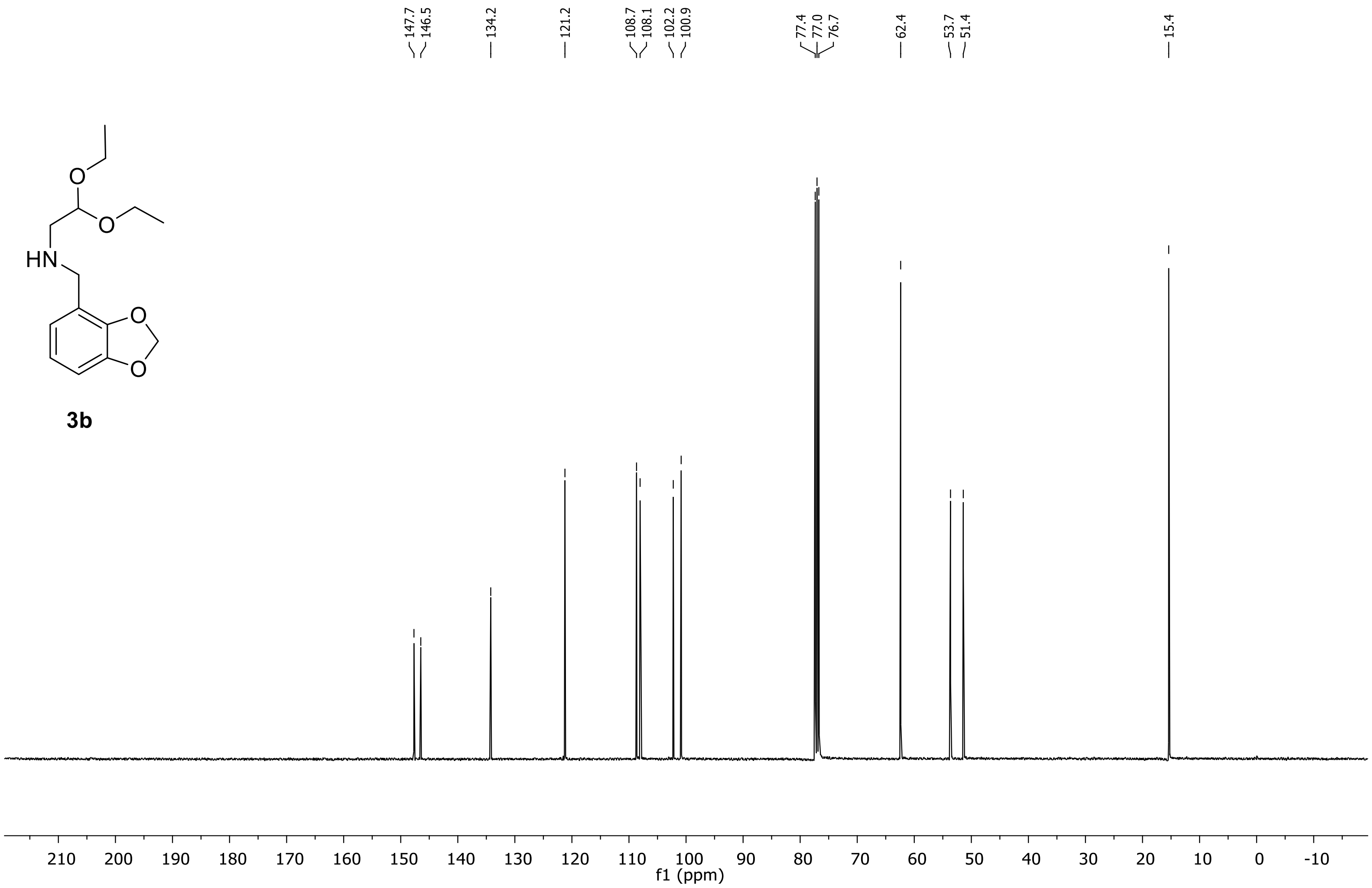

3b

^{1}H NMR of *N*-(3,4,5-trimethoxybenzyl)aminoacetaldehyde diethyl acetal **(3c)**

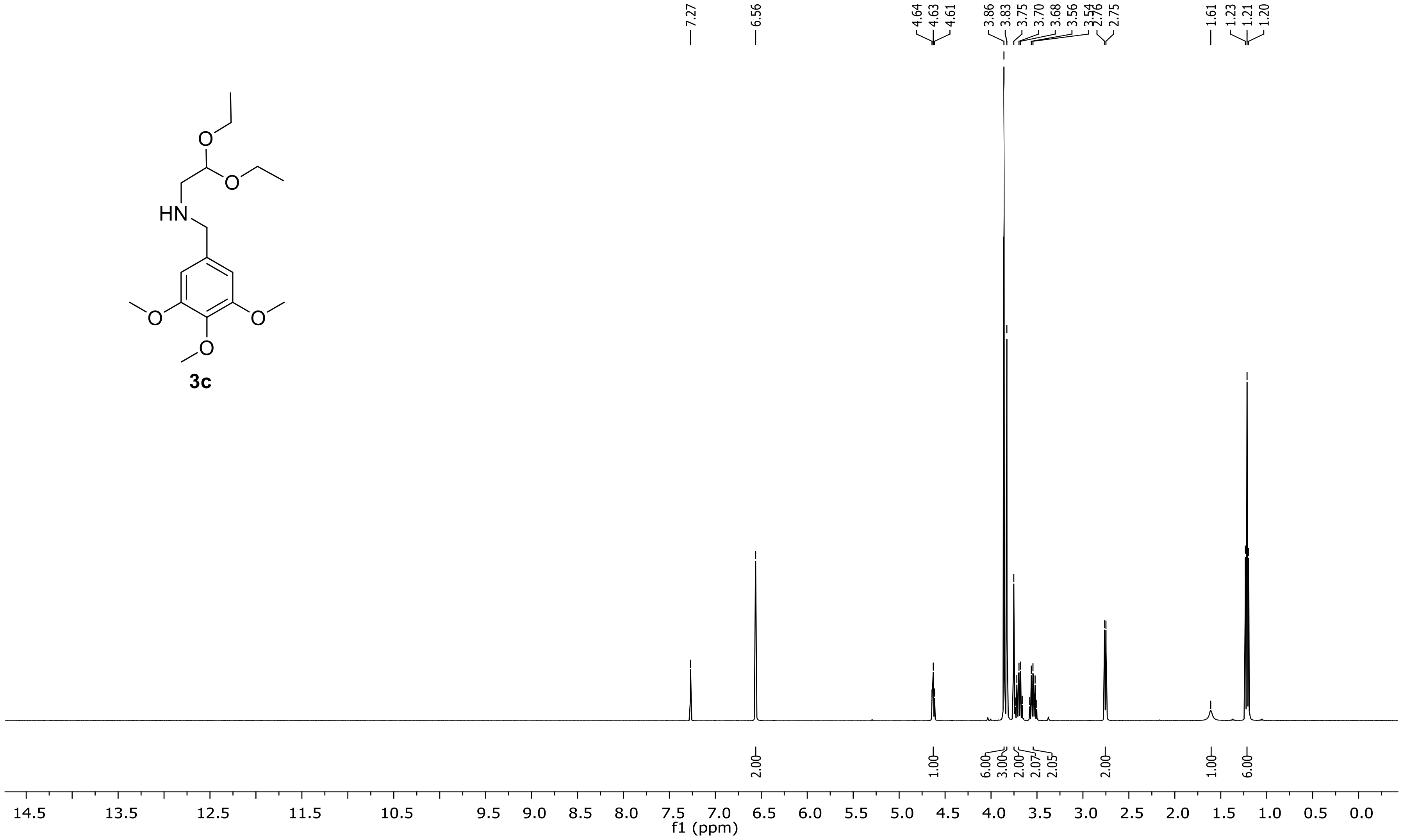

^{13}C NMR of *N*-(3,4,5-trimethoxybenzyl)aminoacetaldehyde diethyl acetal **(3c)**

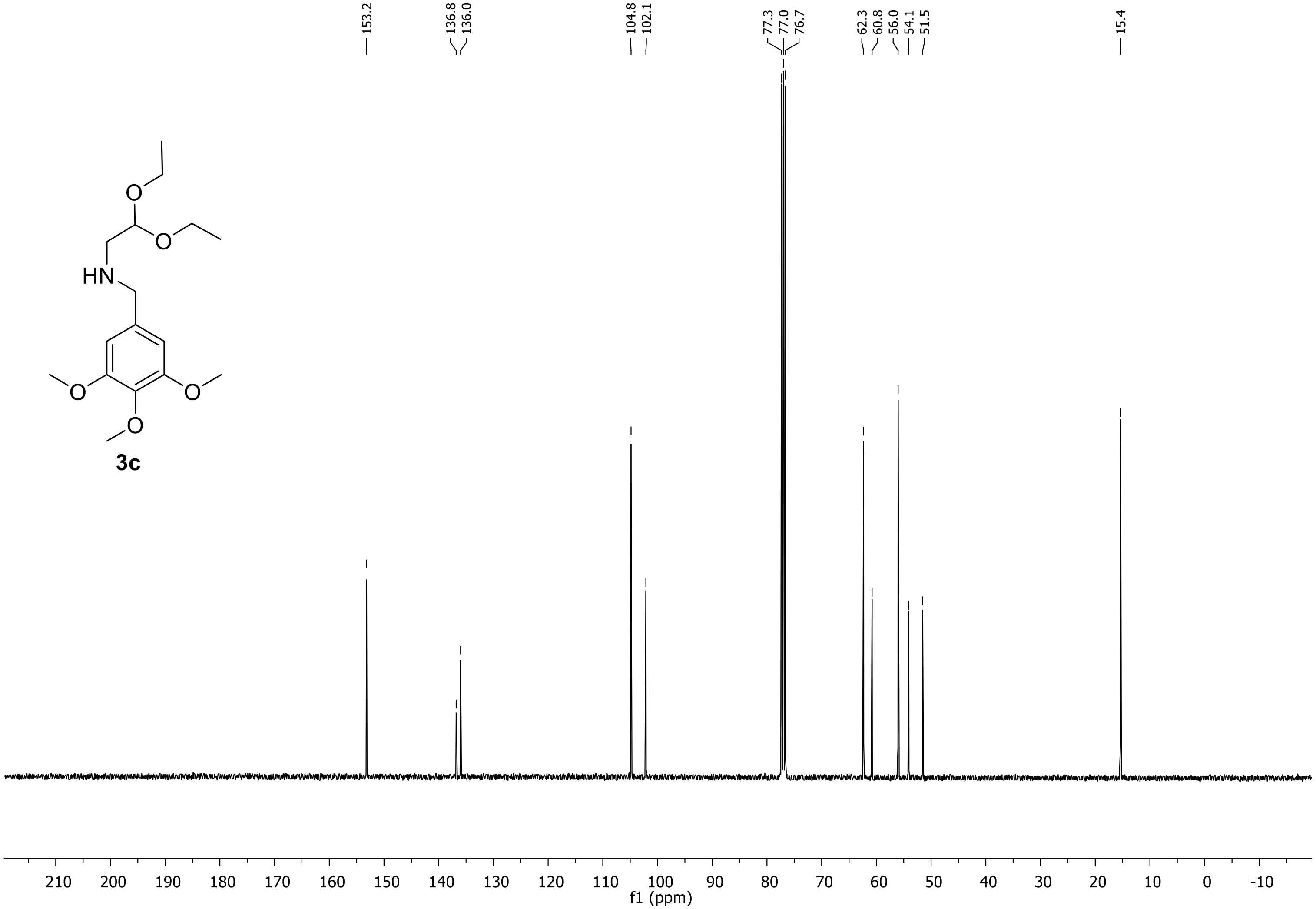

^{1}H NMR of *N*-(3-benzyloxy-2-methoxybenzyl)aminoacetaldehyde diethyl acetal **(3e)**

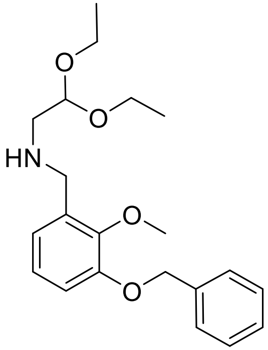

3e

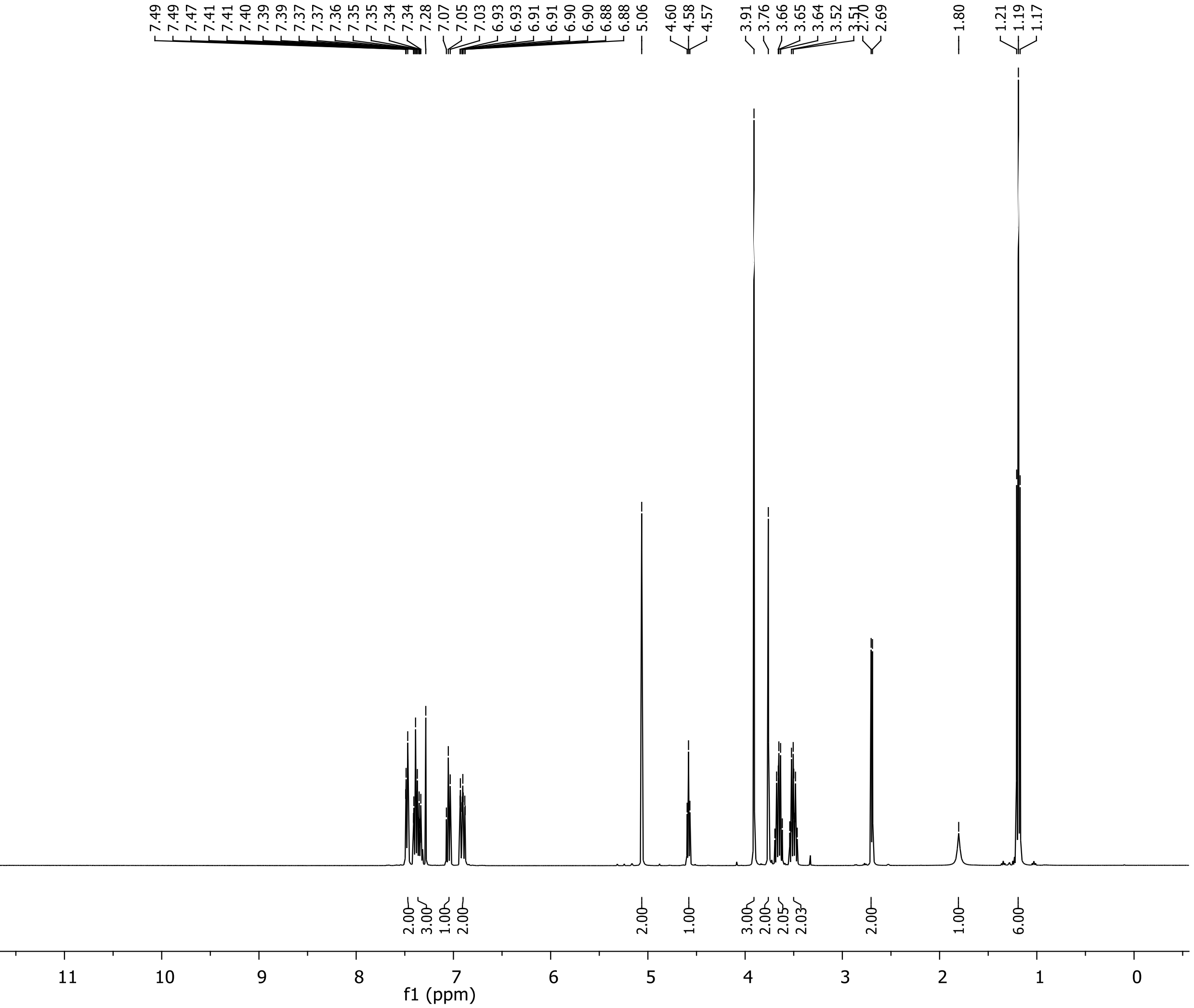

^{13}C NMR of *N*-(3-benzyloxy-2-methoxybenzyl)aminoacetaldehyde diethyl acetal **(3e)**

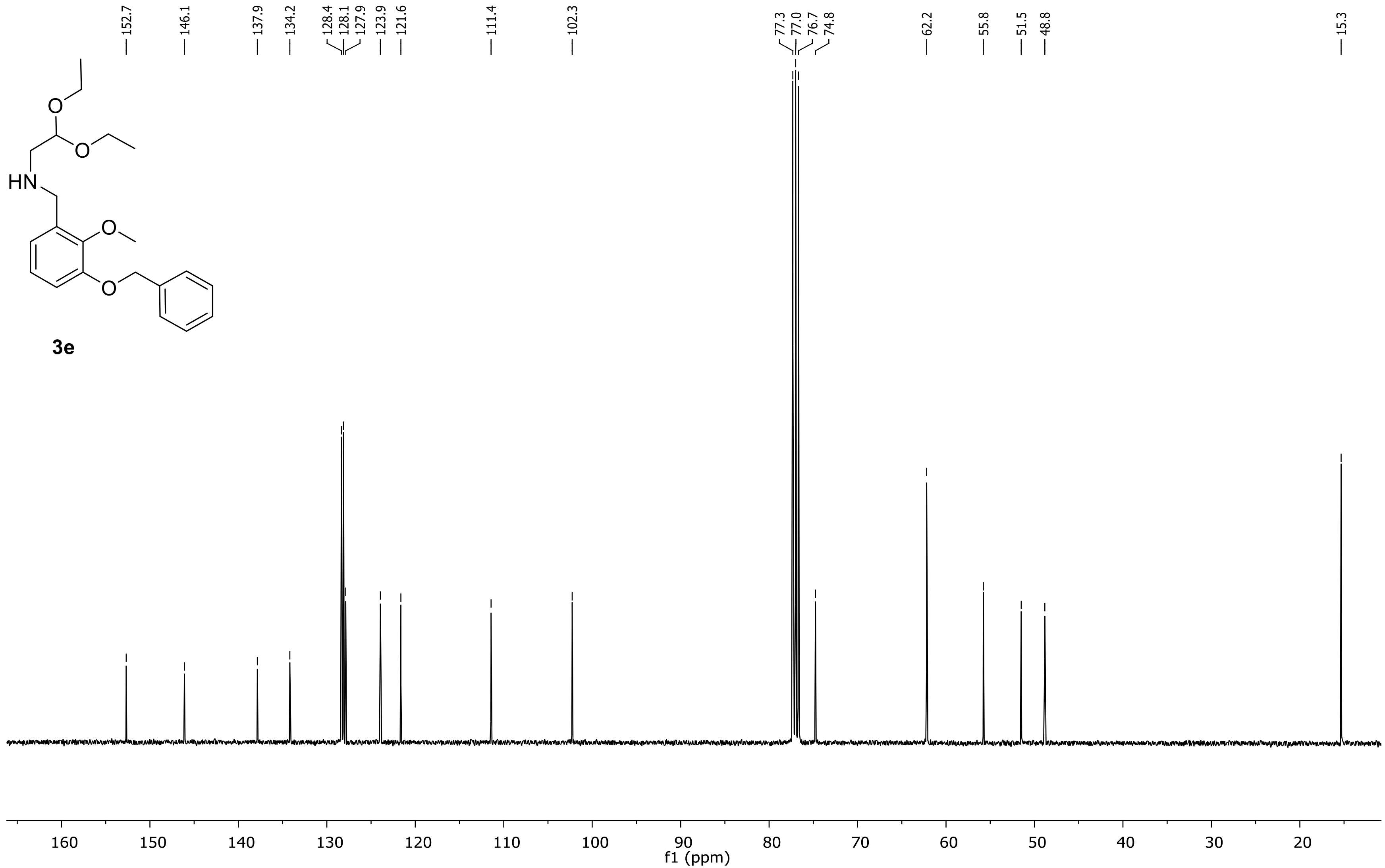

^{1}H NMR of *N*-(2,3-dimethoxybenzyl)-*N*-(2,2-diethoxyethyl)-3,4-dimethoxyphenylglycine **(6a)**

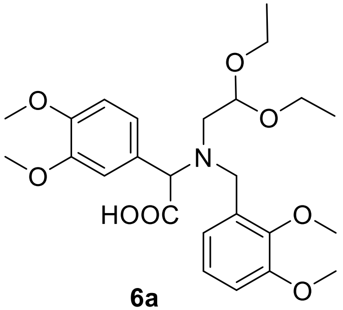

6a

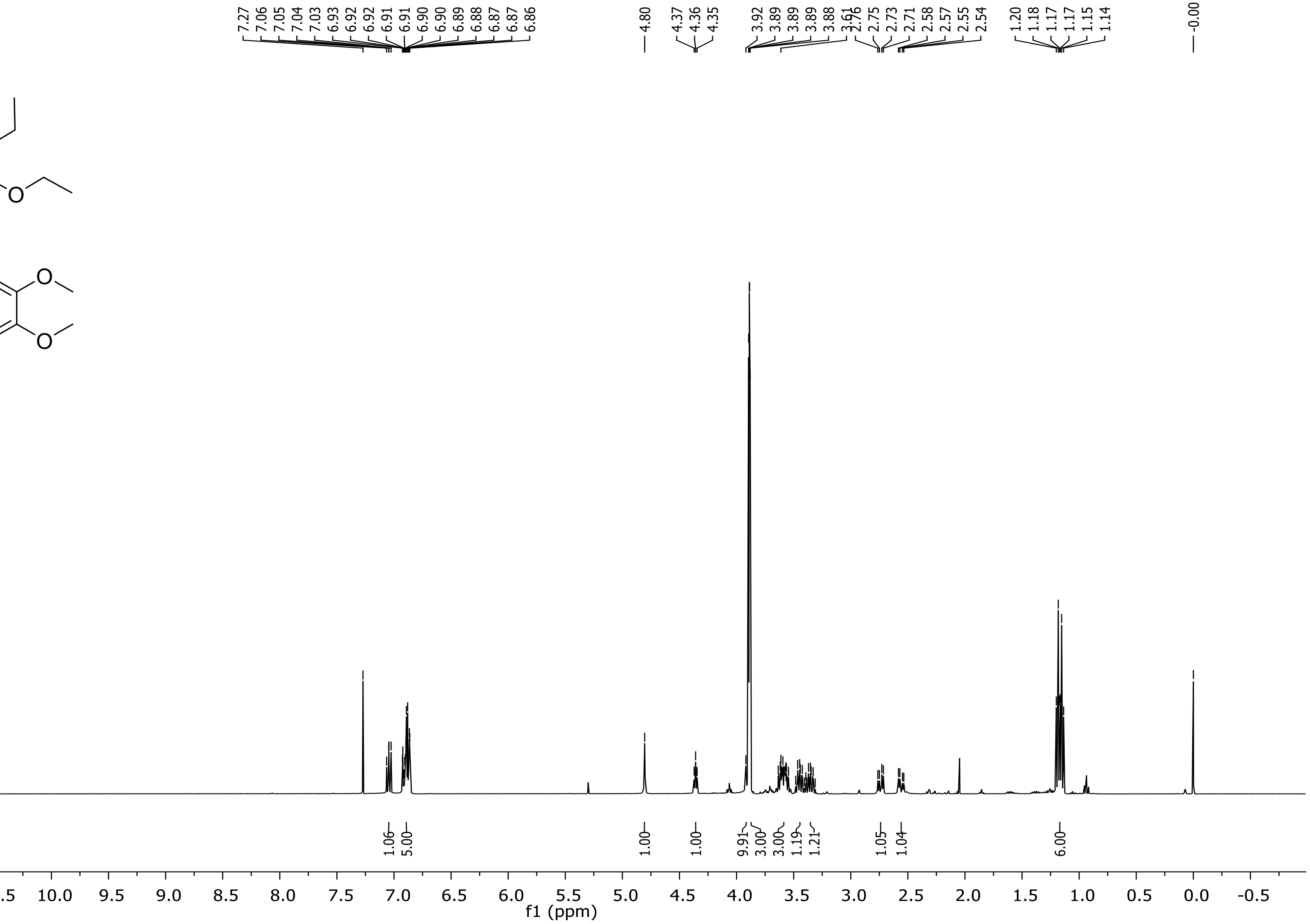

^{13}C NMR of *N*-(2,3-dimethoxybenzyl)-*N*-(2,2-diethoxyethyl)-3,4-dimethoxyphenylglycine **(6a)**

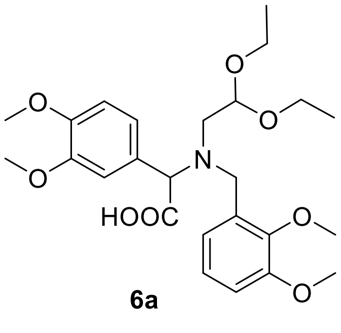

6a

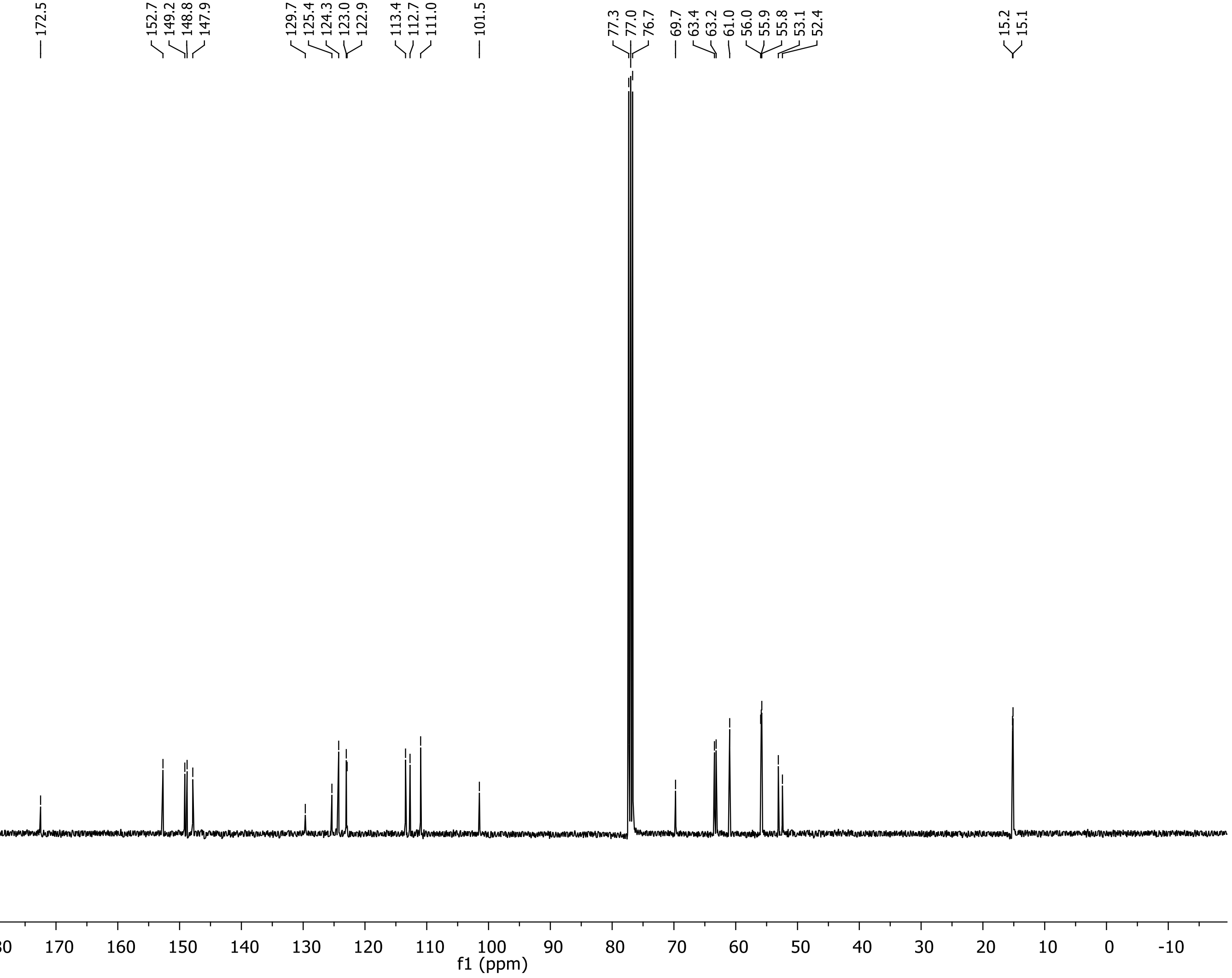

^{1}H NMR of *N*-(2,3-methylenedioxybenzyl)-*N*-(2,2-diethoxyethyl)-3,4-dimethoxyphenylglycine **(6b)**

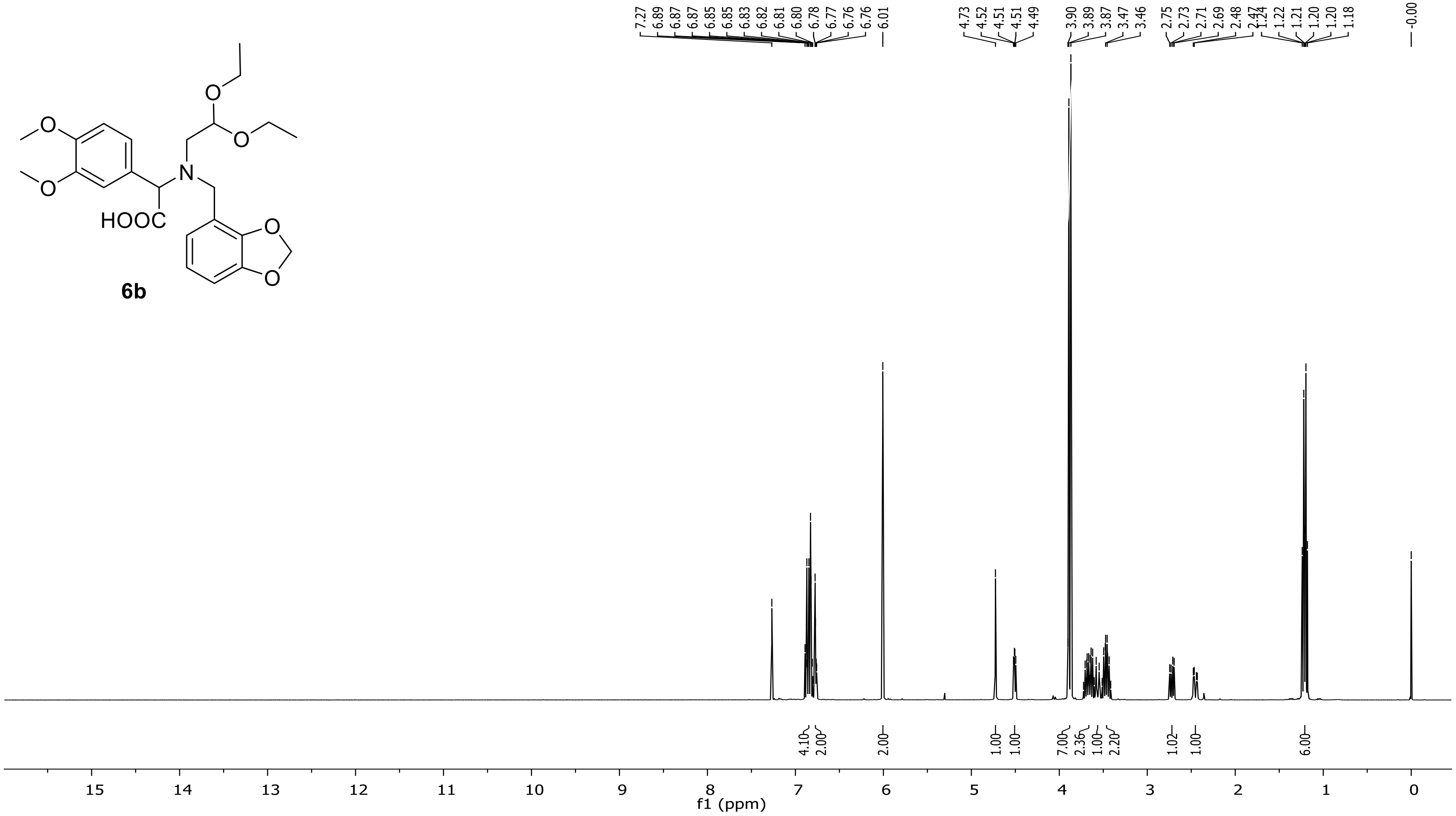

6b

^{13}C NMR of *N*-(2,3-methylenedioxybenzyl)-*N*-(2,2-diethoxyethyl)-3,4-dimethoxyphenylglycine **(6b)**

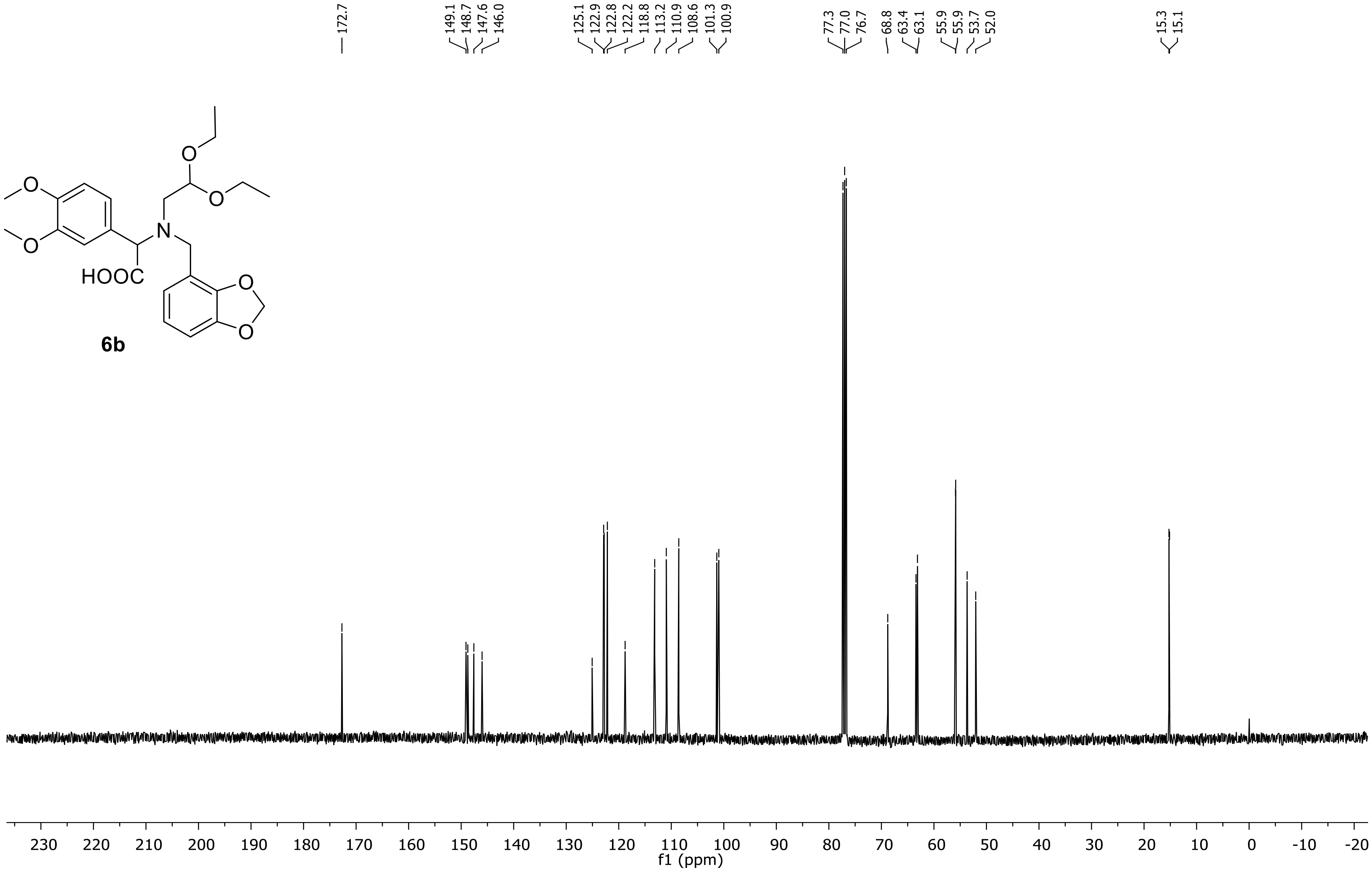

^{1}H NMR of *N*-(3,4,5-trimethoxybenzyl)-*N*-(2,2-diethoxyethyl)-3,4-dimethoxyphenylglycine **(6c)**

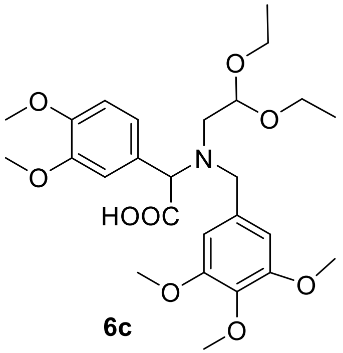

6c

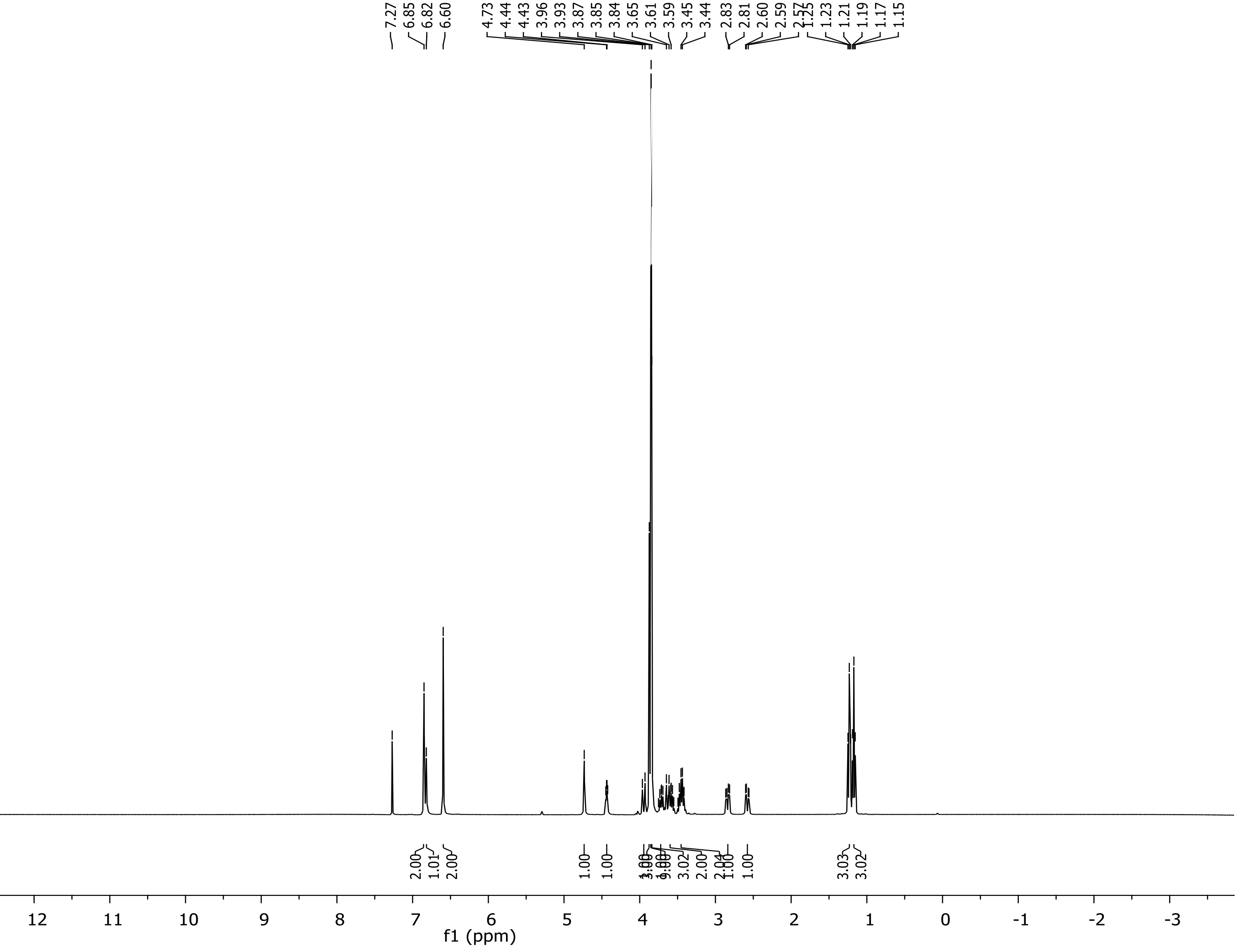

^{13}C NMR of *N*-(3,4,5-trimethoxybenzyl)-*N*-(2,2-diethoxyethyl)-3,4-dimethoxyphenylglycine **(6c)**

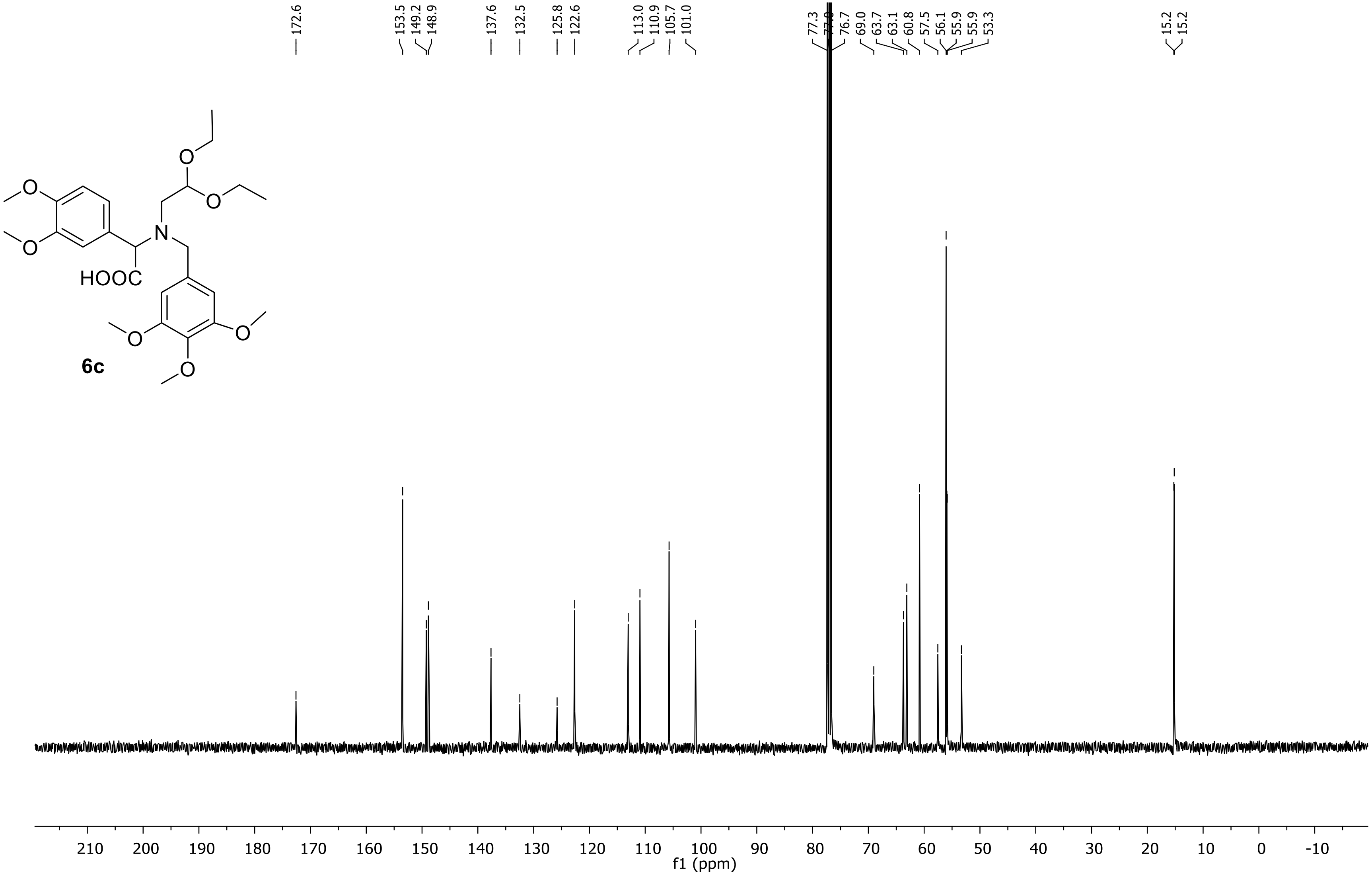

^{1}H NMR of *N*-(2,3-methylenedioxybenzyl)-*N*-(2,2-diethoxyethyl)-3-methoxyphenylglycine **(6d)**

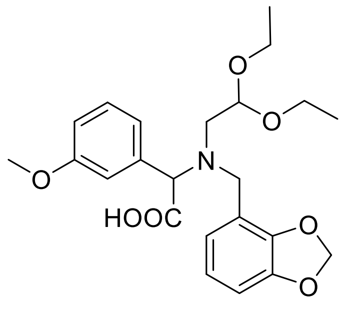

6d

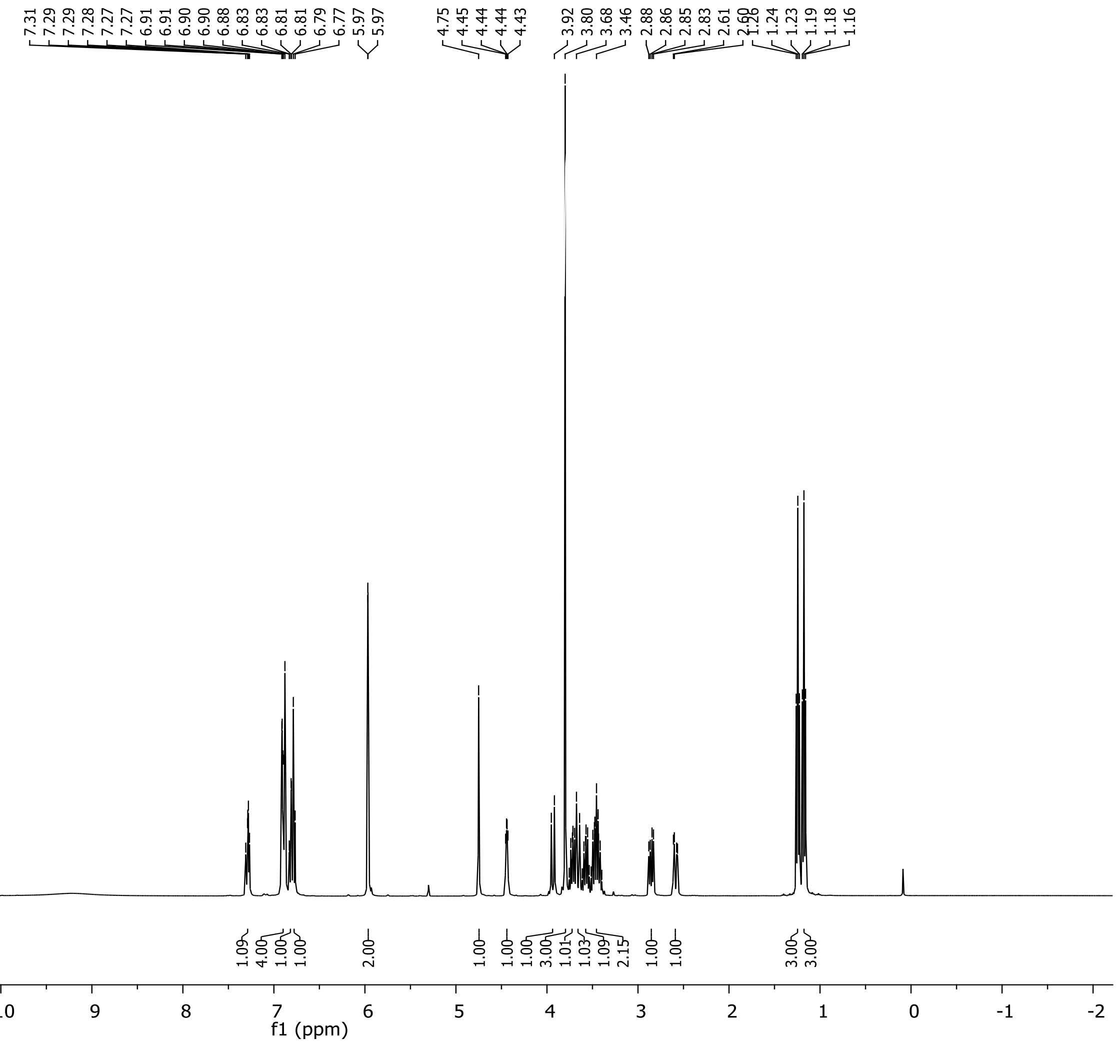

^{13}C NMR of *N*-(2,3-methylenedioxybenzyl)-*N*-(2,2-diethoxyethyl)-3-methoxyphenylglycine **(6d)**

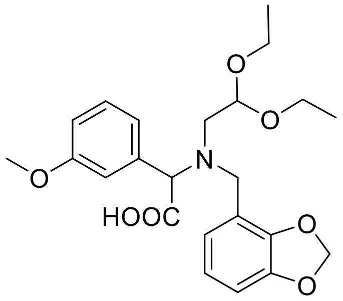

6d

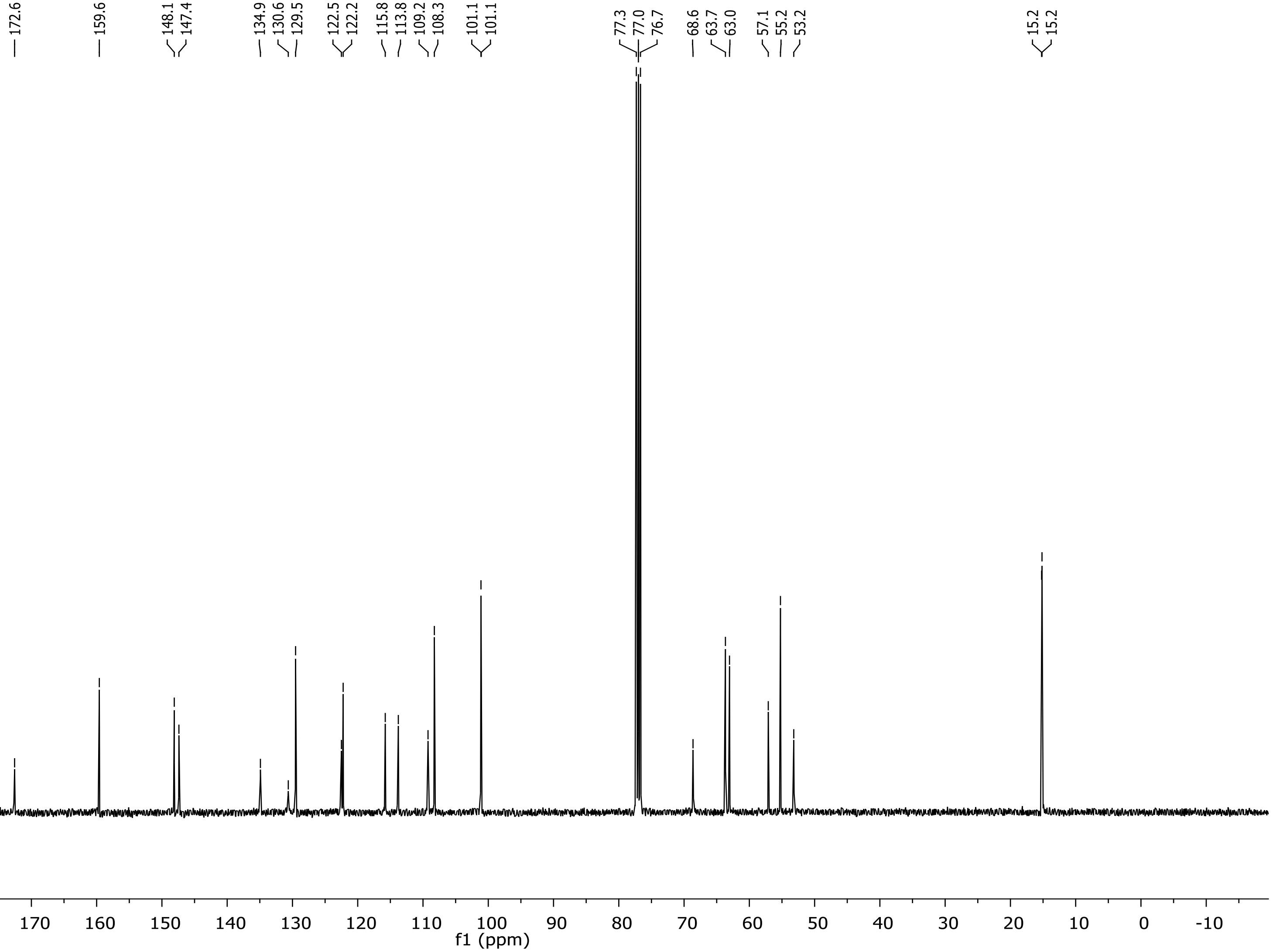

^{1}H NMR of *N*-(2,3-dimethoxybenzyl)-*N*-(2,2-diethoxyethyl)-3,4-methylenedioxyphenylglycine **(6e)**

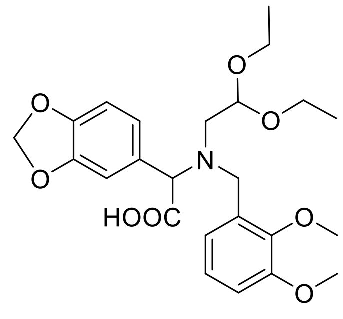

6e

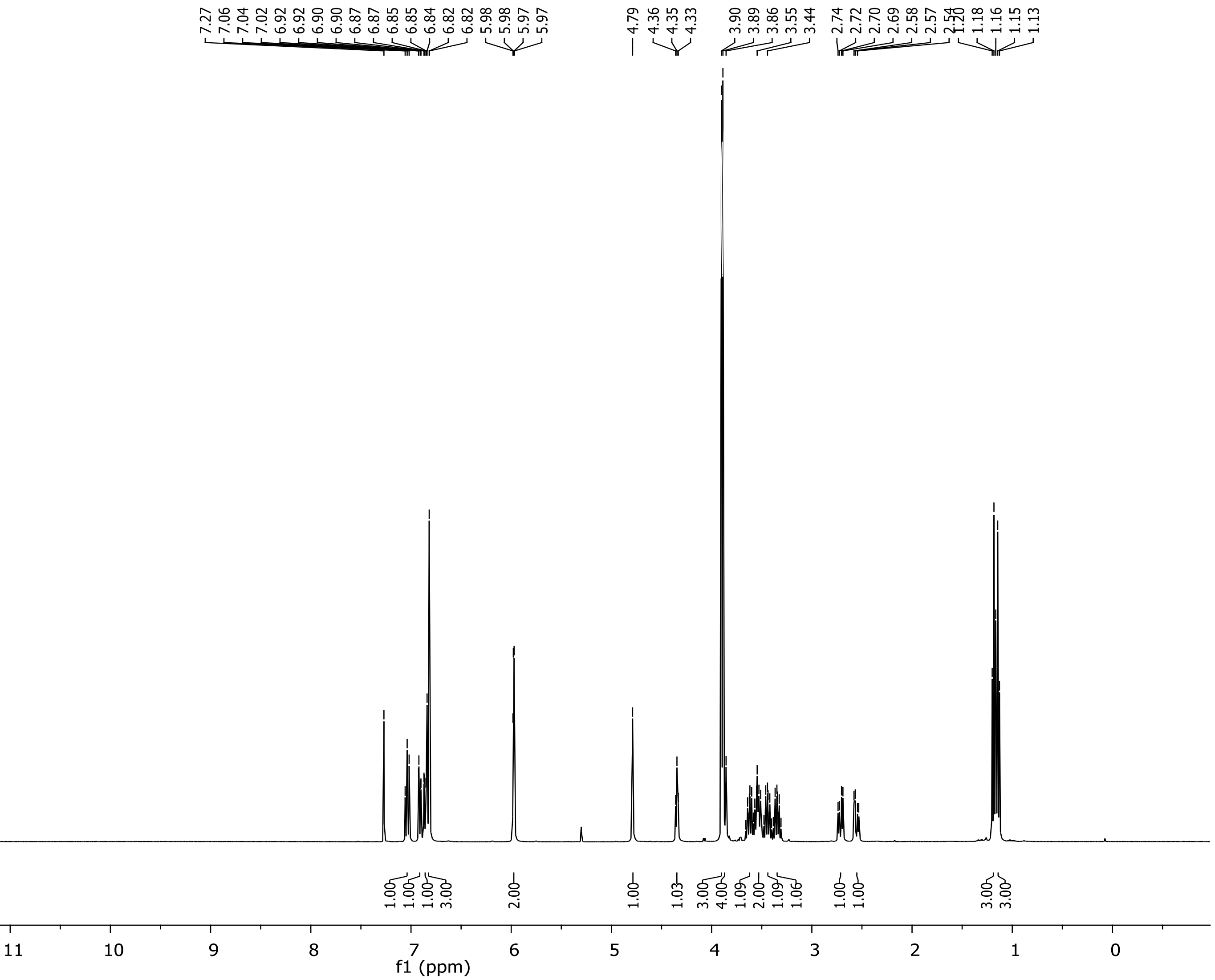

^{13}C NMR of *N*-(2,3-dimethoxybenzyl)-*N*-(2,2-diethoxyethyl)-3,4-methylenedioxyphenylglycine **(6e)**

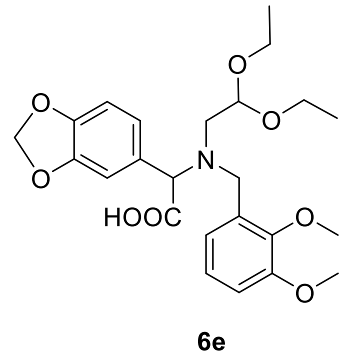

6e

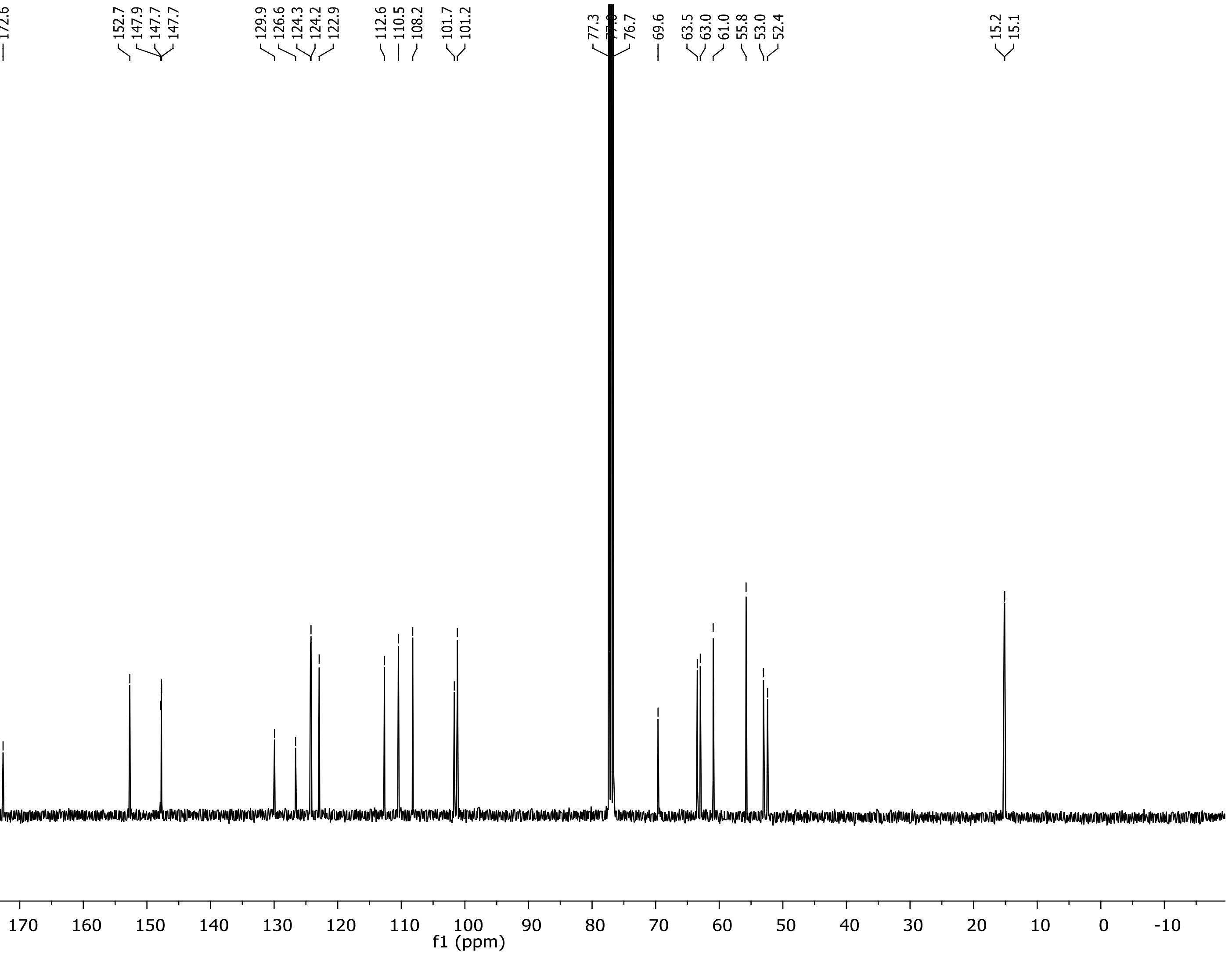

^{1}H NMR of *N*-benzyl-*N*-(2,2-diethoxyethyl)phenylglycine **(6f)**

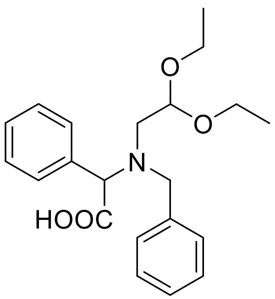

6f

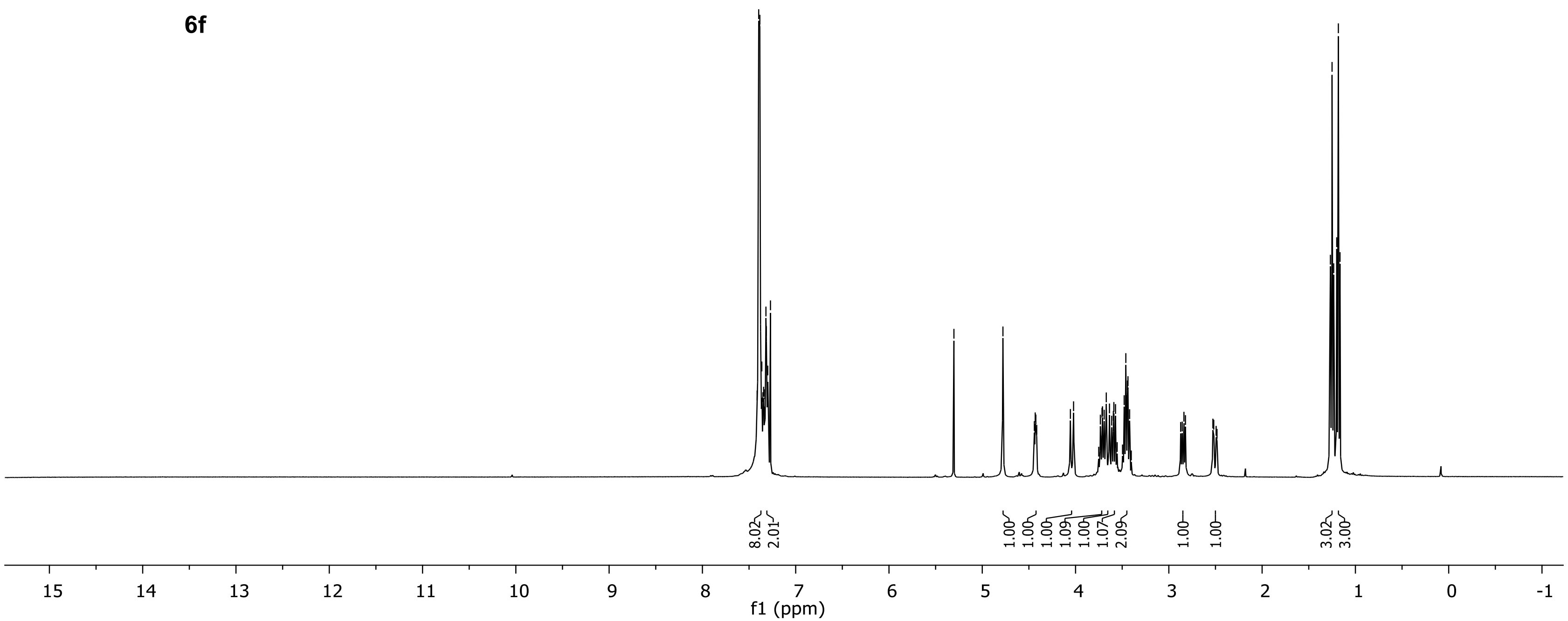

^{13}C NMR of *N*-benzyl-*N*-(2,2-diethoxyethyl)phenylglycine **(6f)**

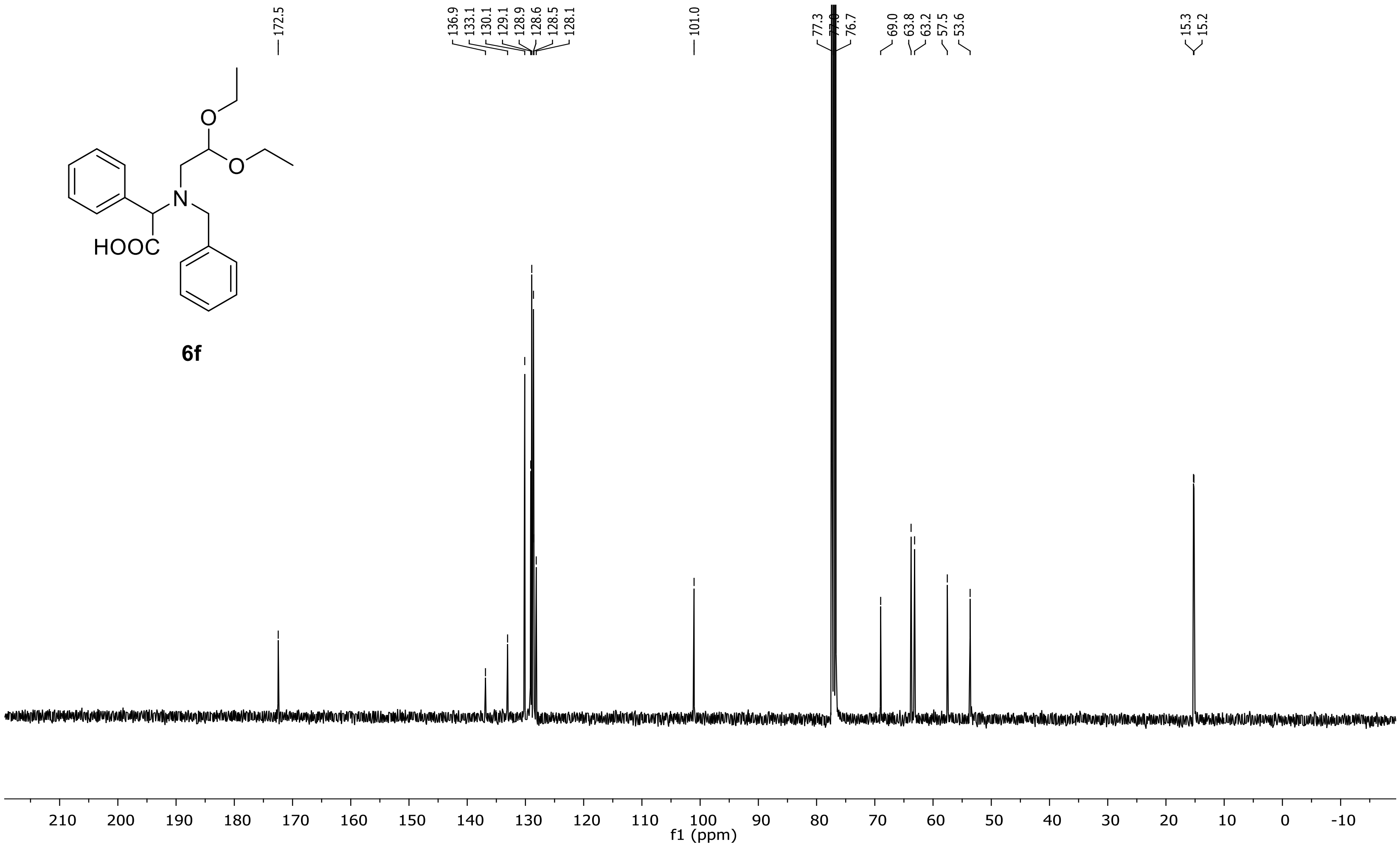

^{1}H NMR of *N*-(3-benzyloxy-2-methoxybenzyl)-*N*-(2,2-diethoxyethyl)-3,4-dimethoxyphenylglycine **(6g)**

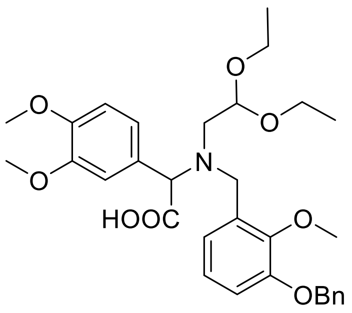

6g

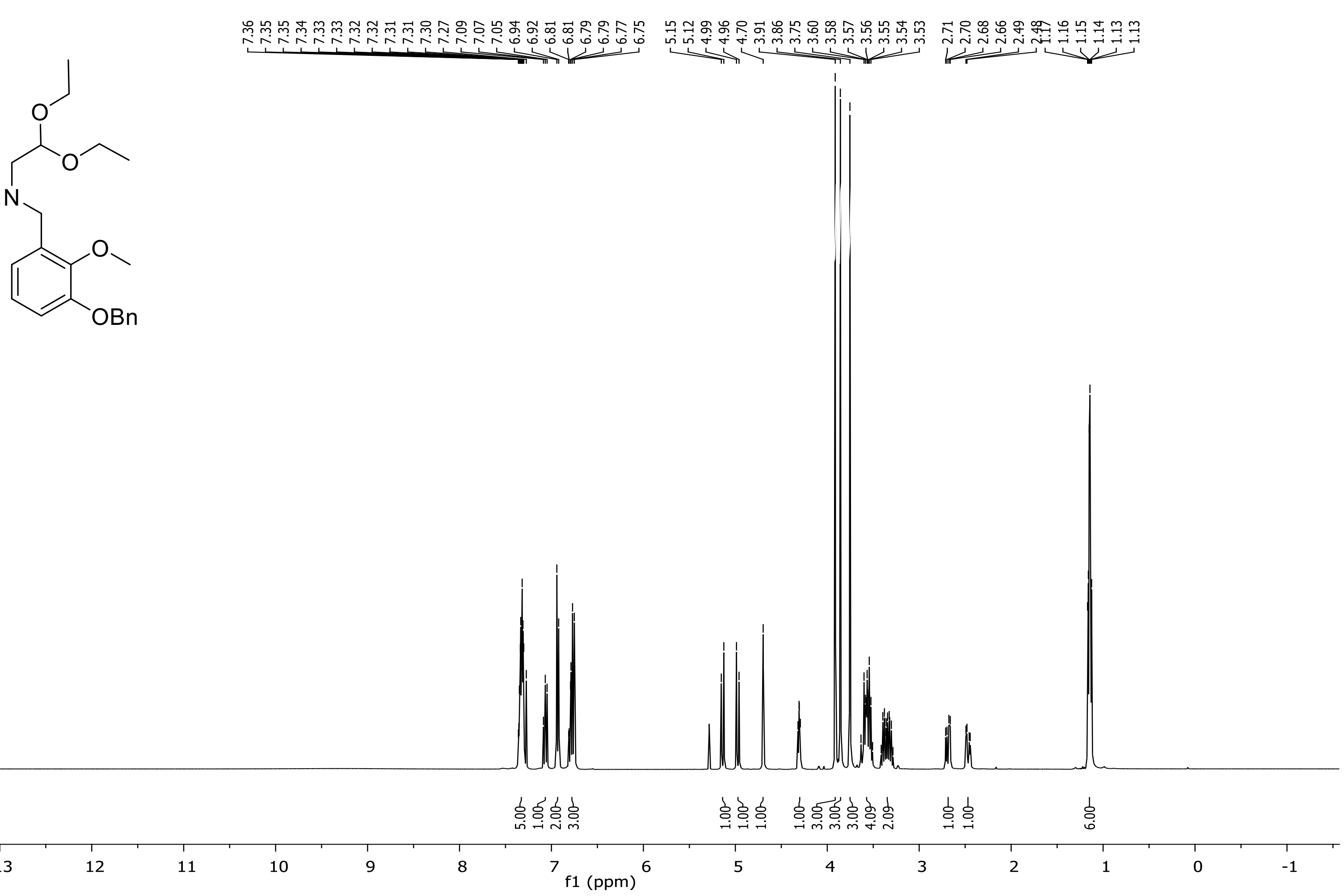

^{13}C NMR of *N*-(3-benzyloxy-2-methoxybenzyl)-*N*-(2,2-diethoxyethyl)-3,4-dimethoxyphenylglycine **(6g)**

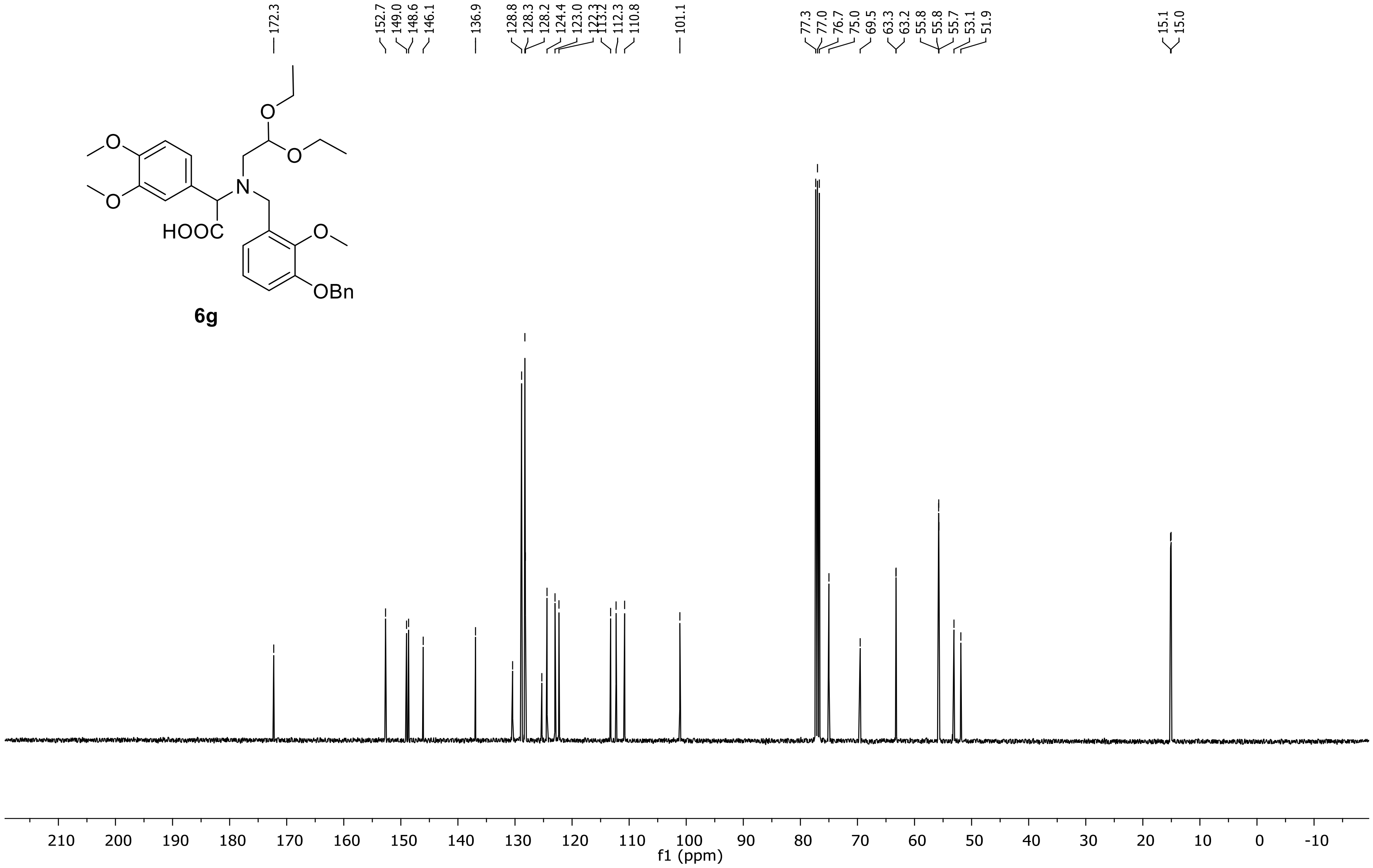

^{1}H NMR of 2,3,8,9-tetramethoxy-7,12-dihydro-6,12-methanodibenzo[*c*,*f*]azocine-5-carboxylic acid **(7a)**

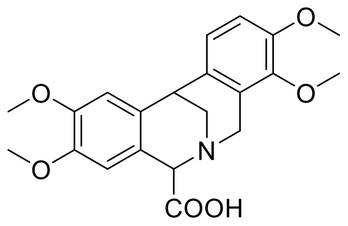

7a

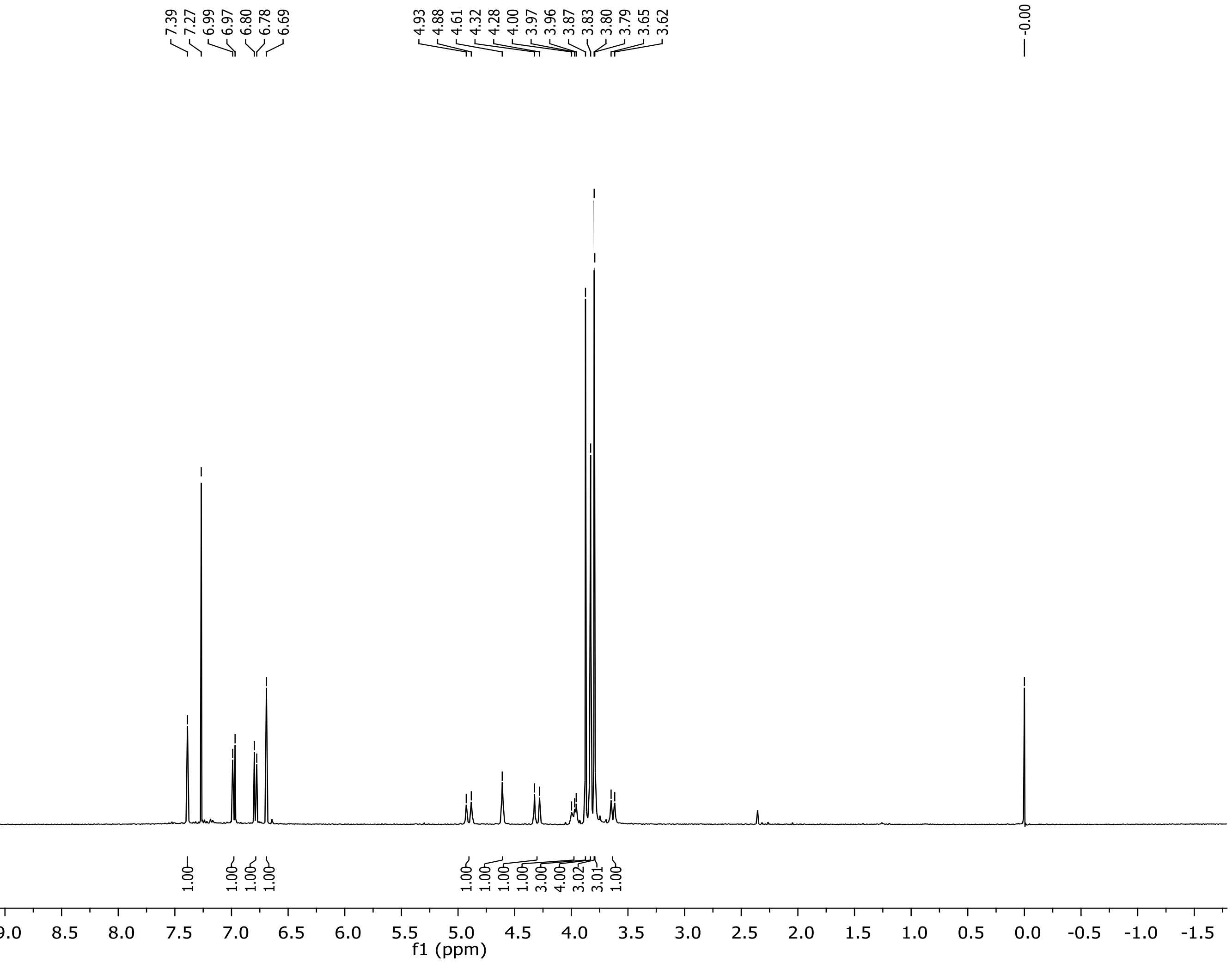

^{13}C NMR of 2,3,8,9-tetramethoxy-7,12-dihydro-6,12-methanodibenzo[*c*,*f*]azocine-5-carboxylic acid **(7a)**

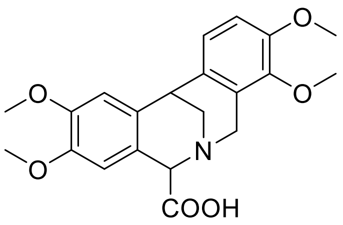

7a

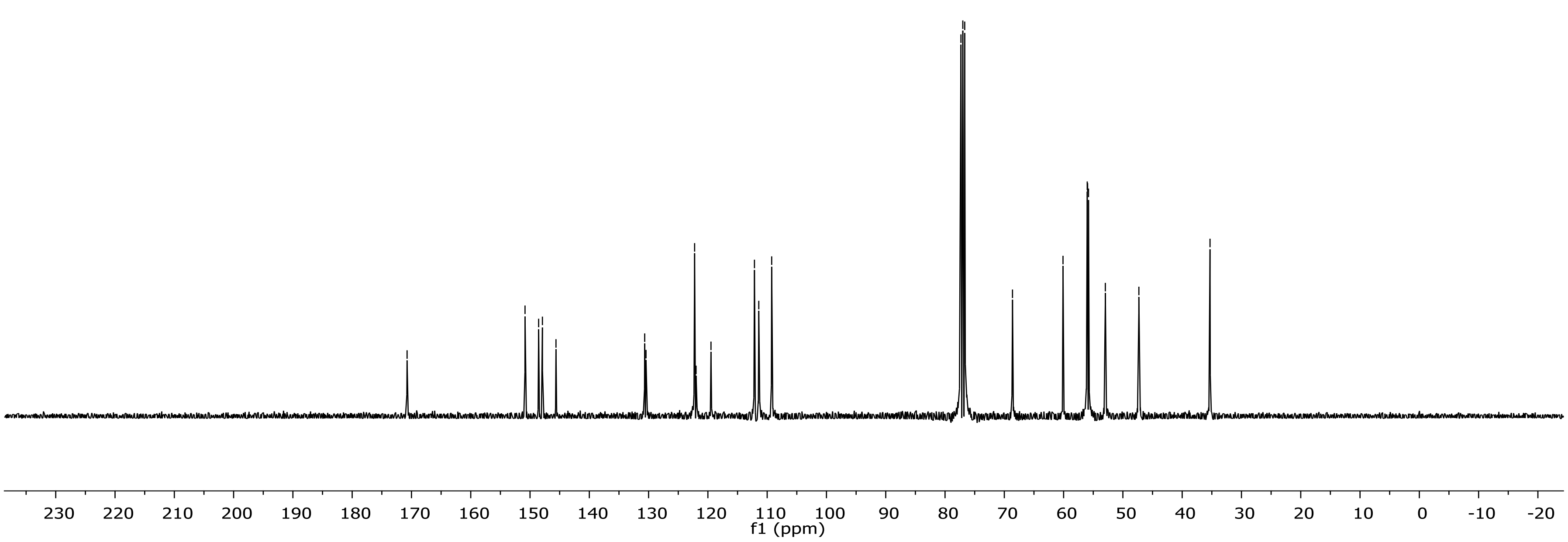

^{1}H NMR of 2,3-dimethoxy-8,9-methylenedioxy-7,12-dihydro-6,12-methanodibenzo[*c*,*f*]azocine-5-carboxylic acid **(7b)**

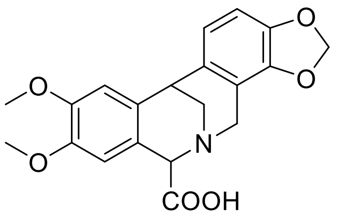

7b

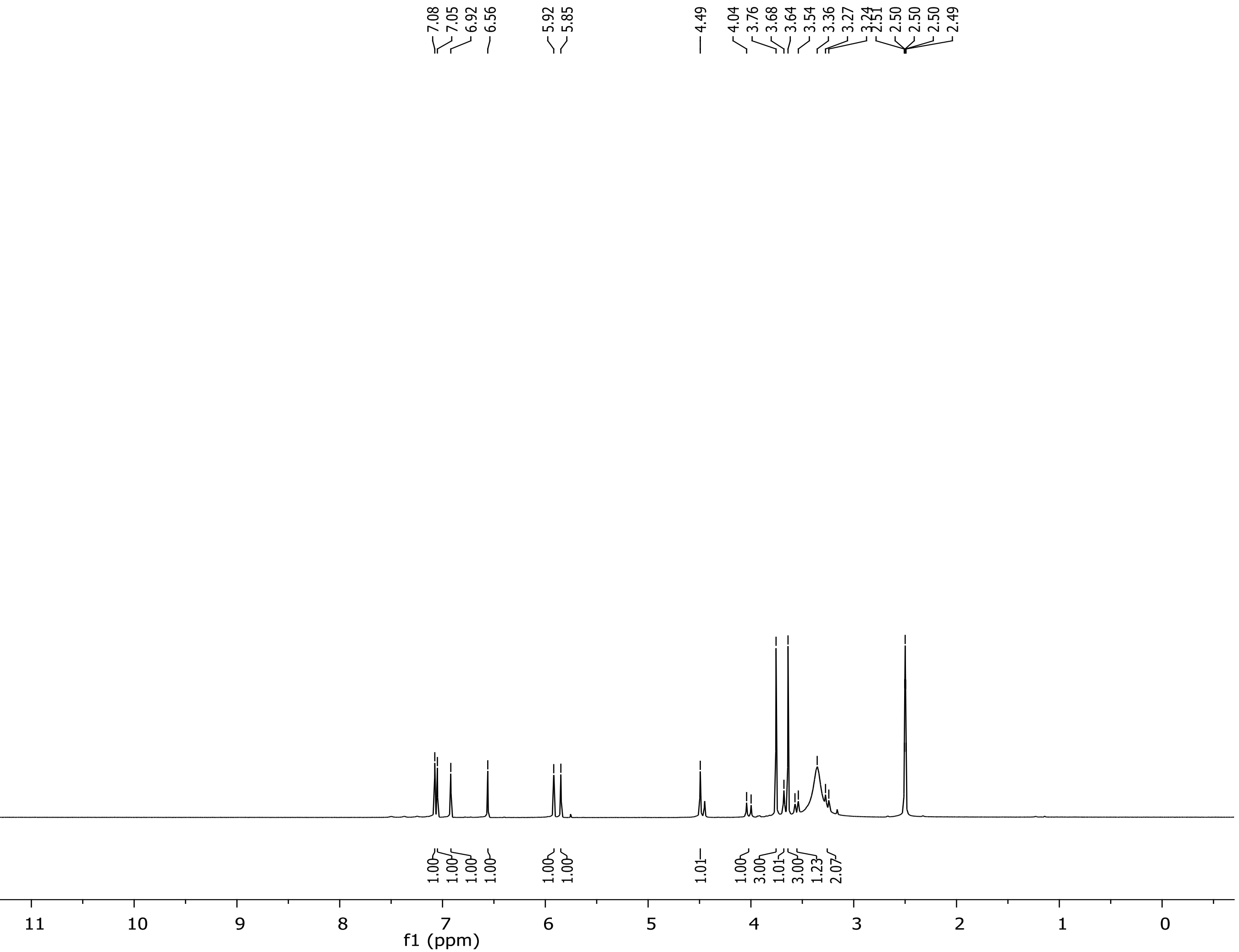

^{13}C NMR of 2,3-dimethoxy-8,9-methylenedioxy-7,12-dihydro-6,12-methanodibenzo[*c*,*f*]azocine-5-carboxylic acid **(7b)**

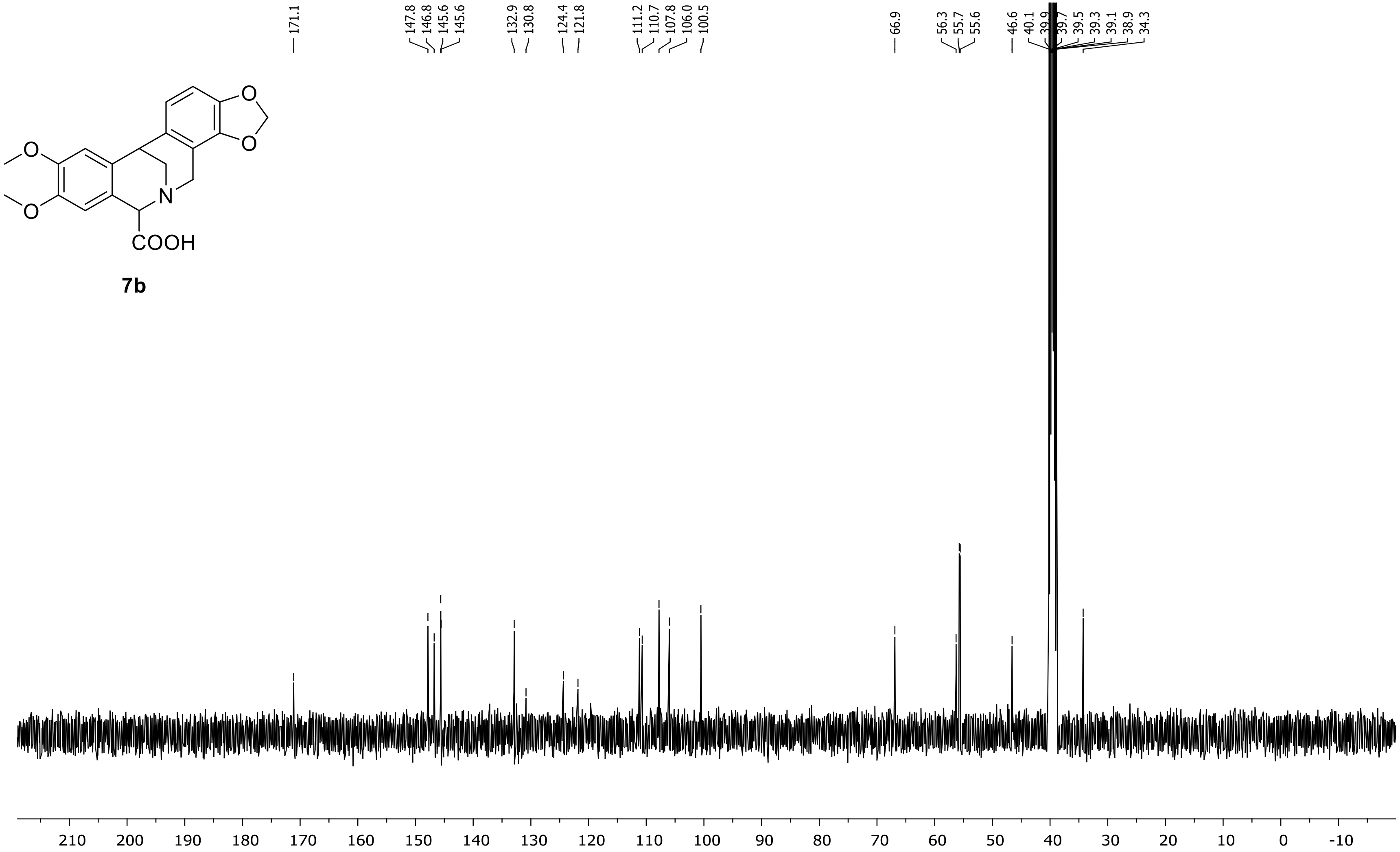

^{1}H NMR of 2,3,9,10,11-pentamethoxy-7,12-dihydro-6,12-methanodibenzo[*c*,*f*]azocine-5-carboxylic acid **(7c)**

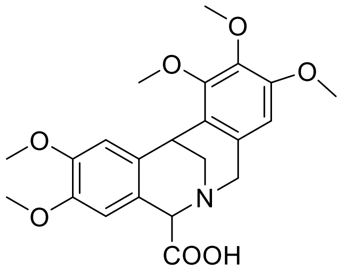

7c

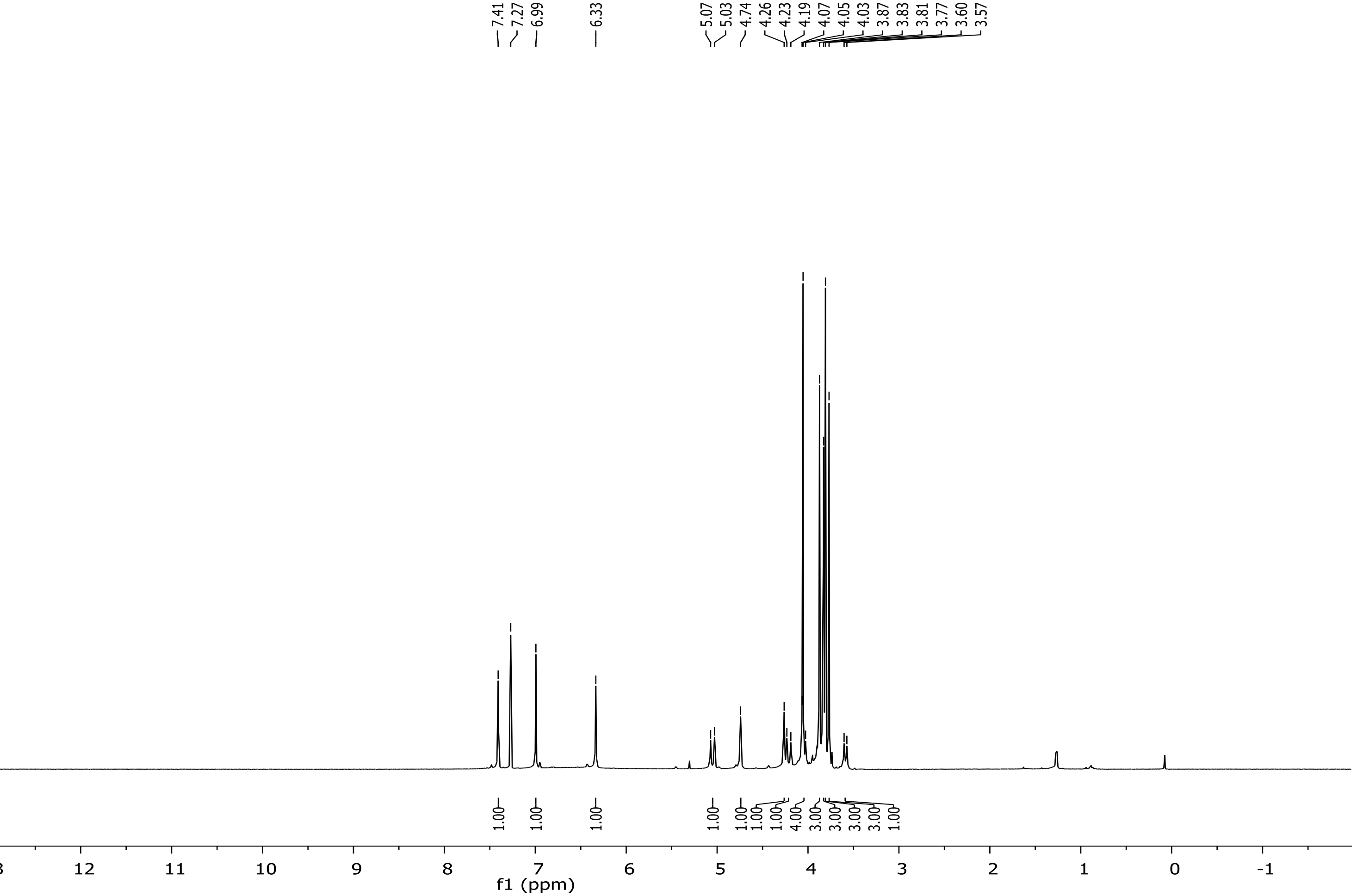

^{13}C NMR of 2,3,9,10,11-pentamethoxy-7,12-dihydro-6,12-methanodibenzo[*c*,*f*]azocine-5-carboxylic acid **(7c)**

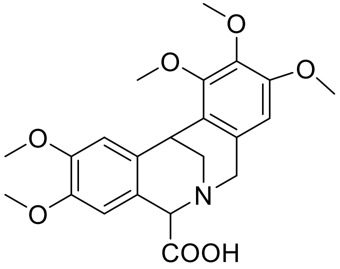

7c

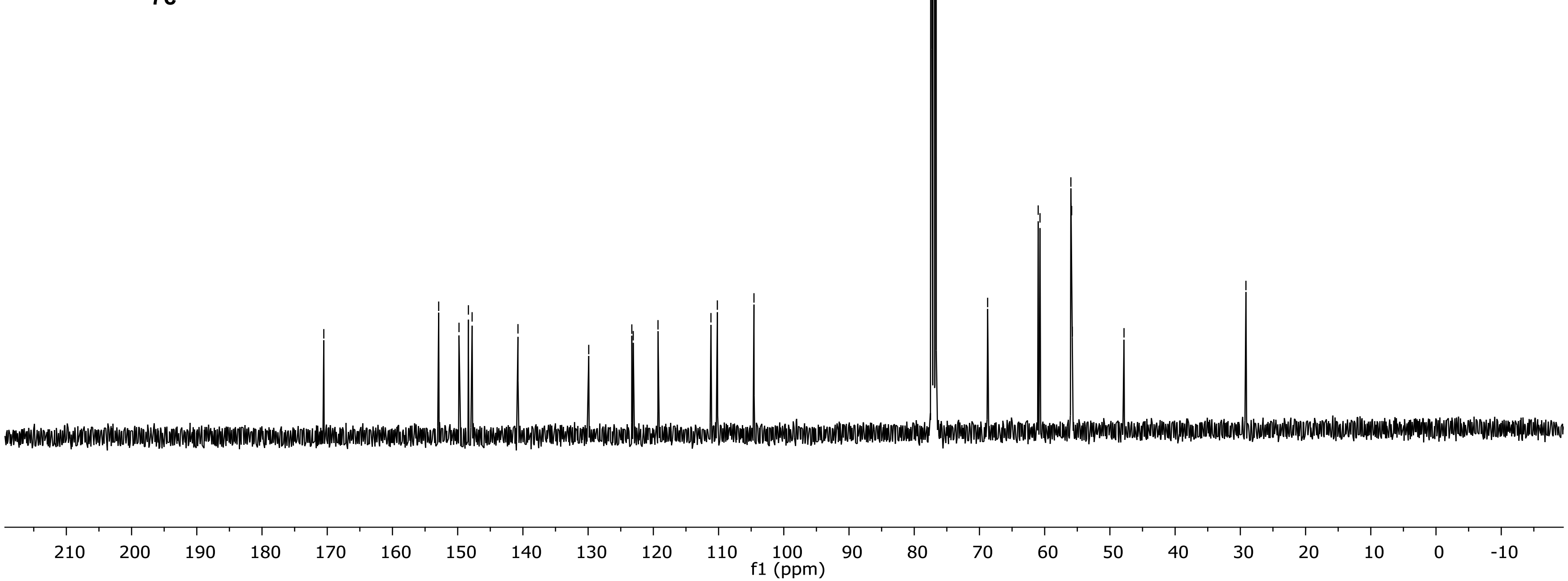

^{1}H NMR of 3-methoxy-8,9-methylenedioxy-7,12-dihydro-6,12-methanodibenzo[*c*,*f*]azocine-5-carboxylic acid **(7d)**

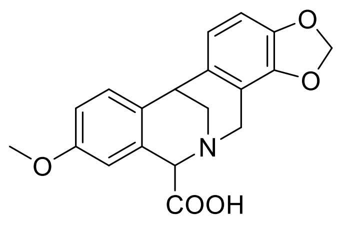

7d

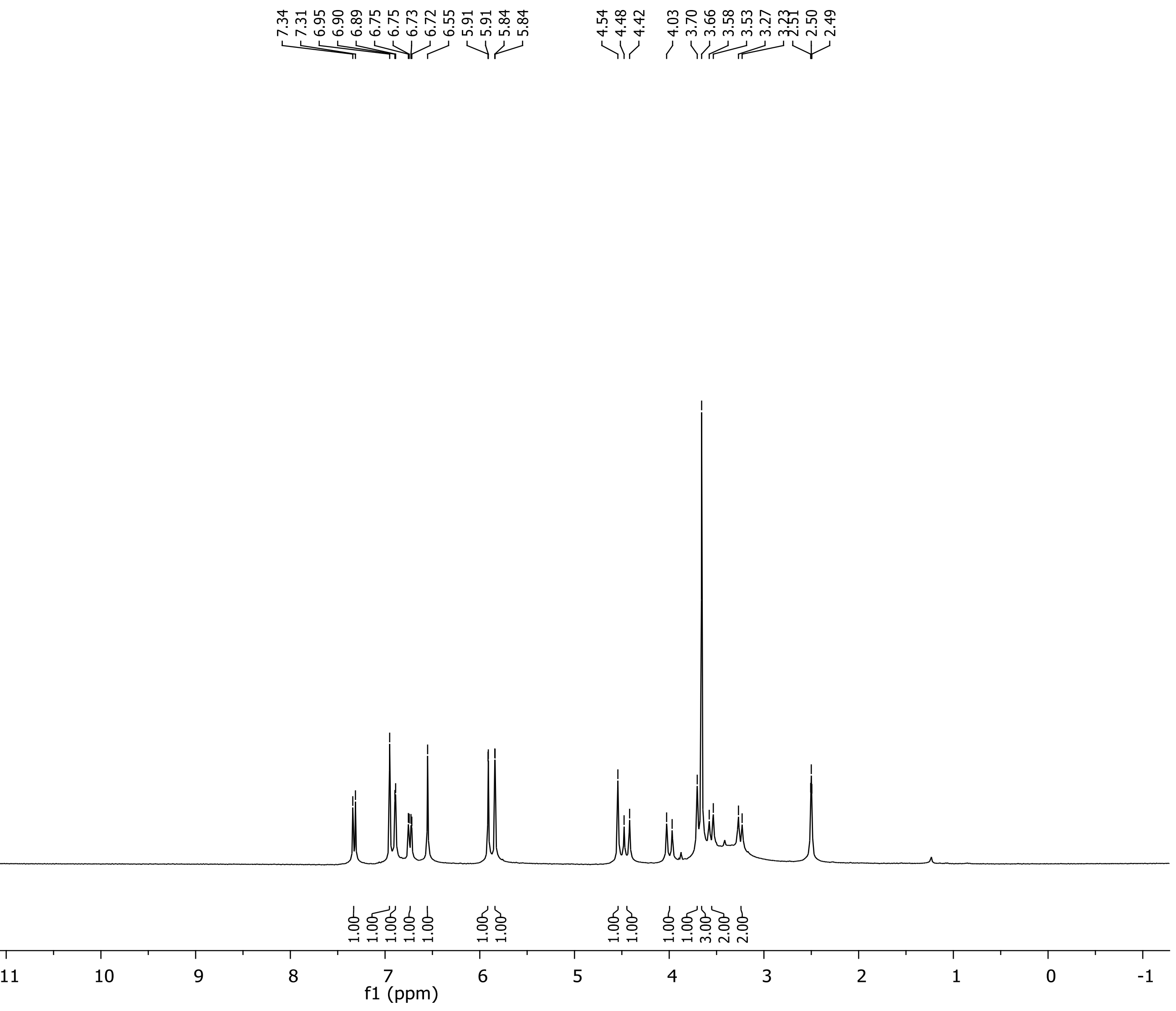

^{13}C NMR of 3-methoxy-8,9-methylenedioxy-7,12-dihydro-6,12-methanodibenzo[*c*,*f*]azocine-5-carboxylic acid **(7d)**

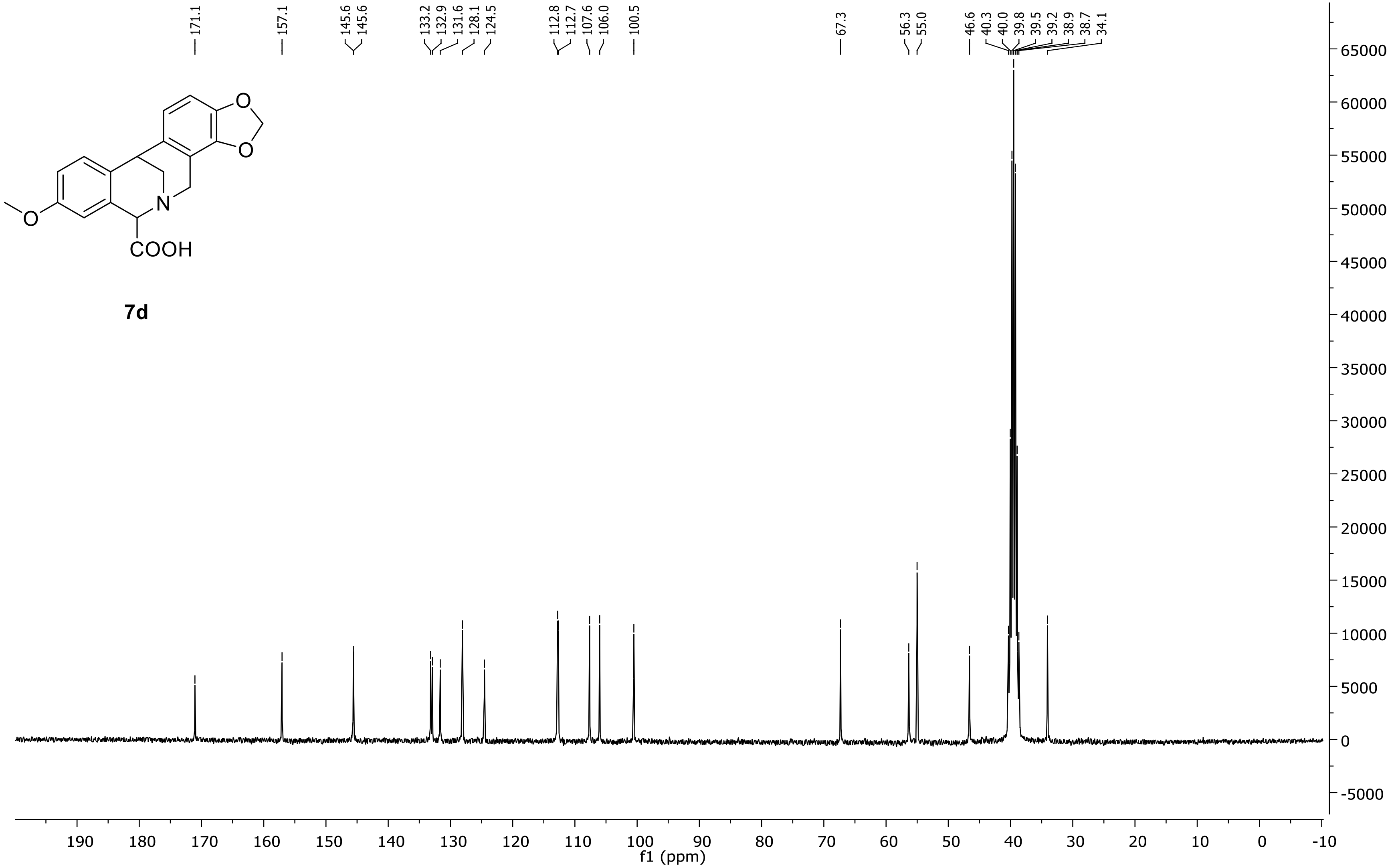

^{1}H NMR of 2,3-methylenedioxy-8,9-dimethoxy-7,12-dihydro-6,12-methanodibenzo[*c*,*f*]azocine-5-carboxylic acid **(7e)**

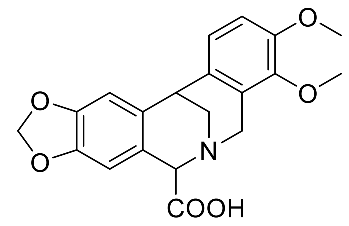

7e

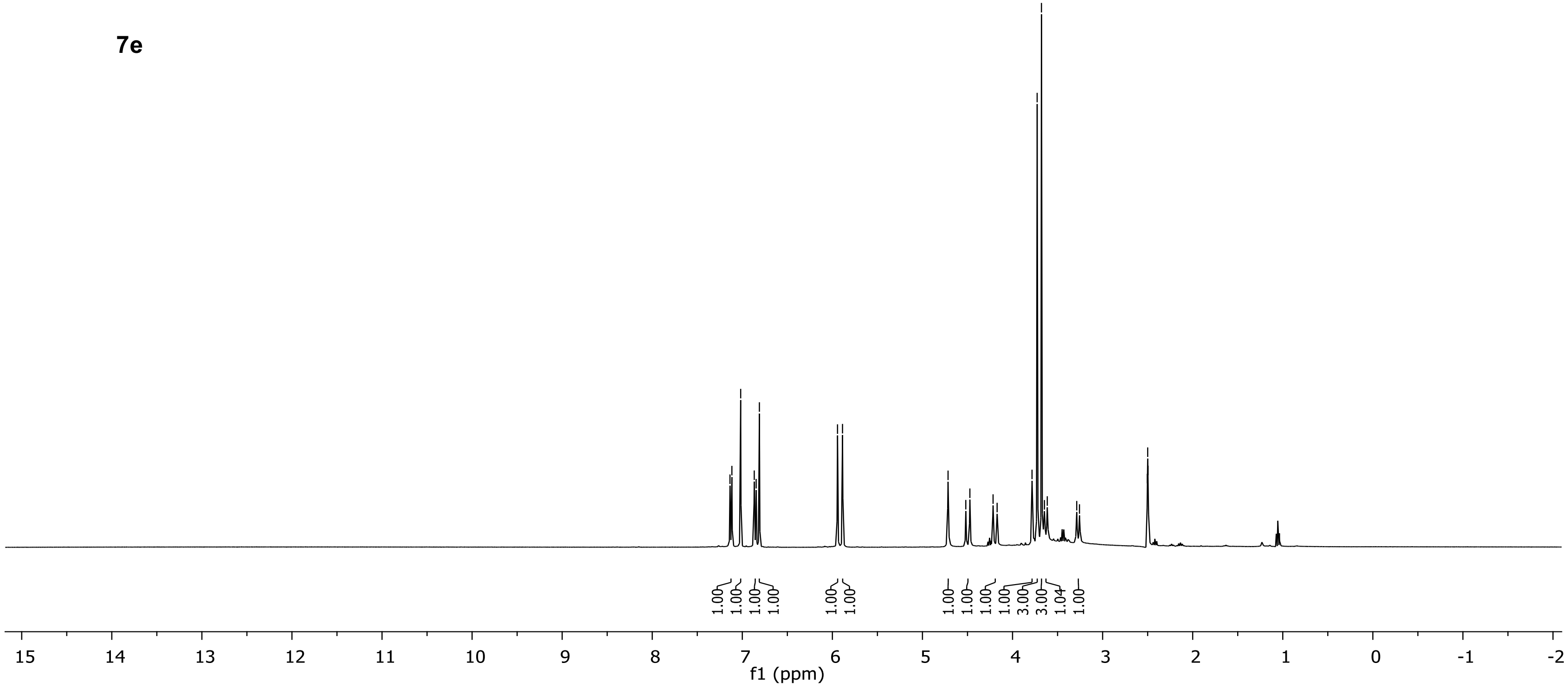

^{13}C NMR of 2,3-methylenedioxy-8,9-dimethoxy-7,12-dihydro-6,12-methanodibenzo[*c*,*f*]azocine-5-carboxylic acid **(7e)**

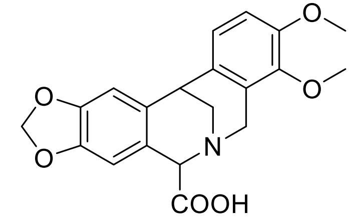

7e

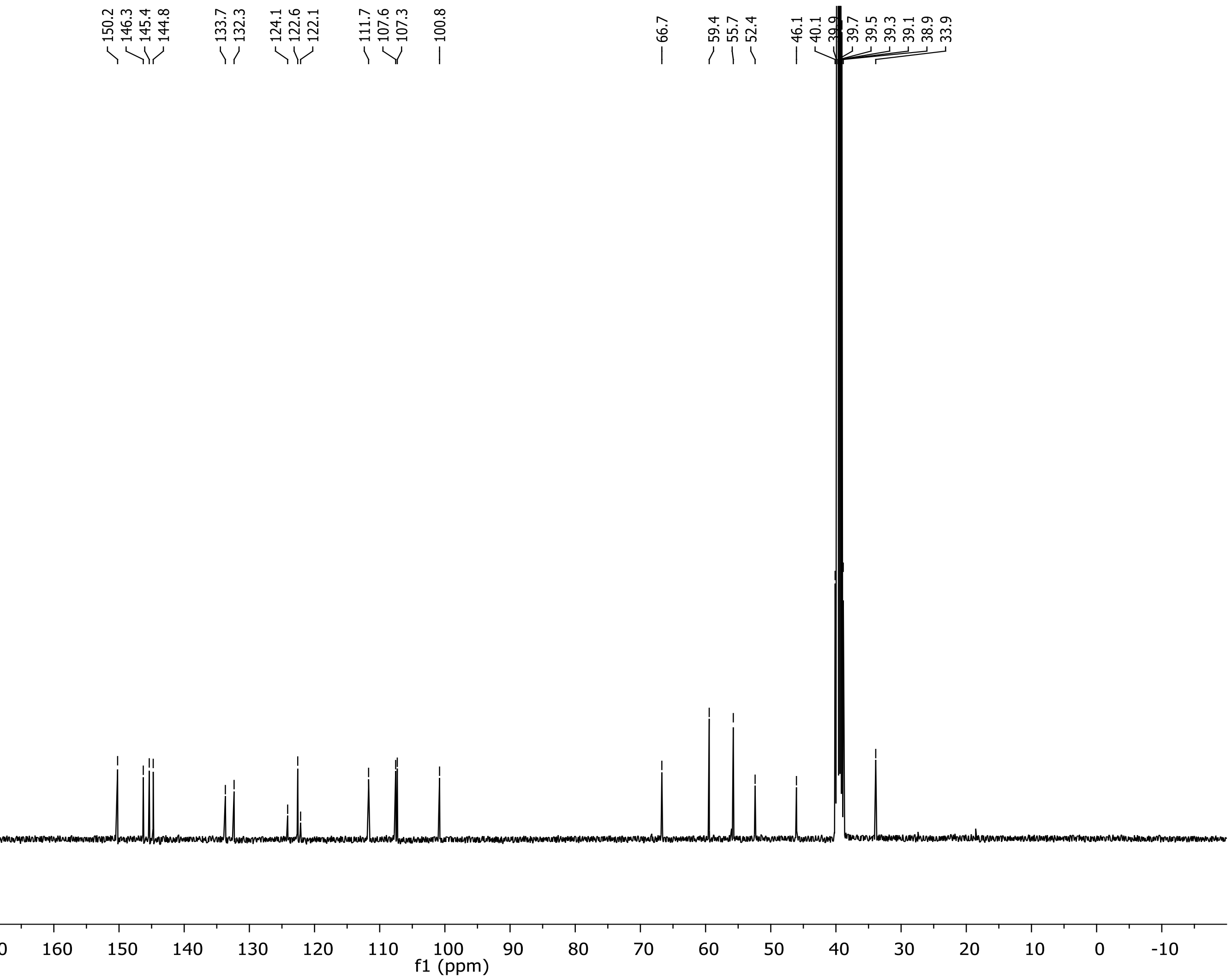

^{1}H NMR of 7,12-dihydro-6,12-methanodibenzo[*c*,*f*]azocine-5-carboxylic acid **(7f)**

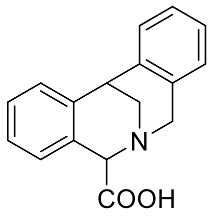

7f

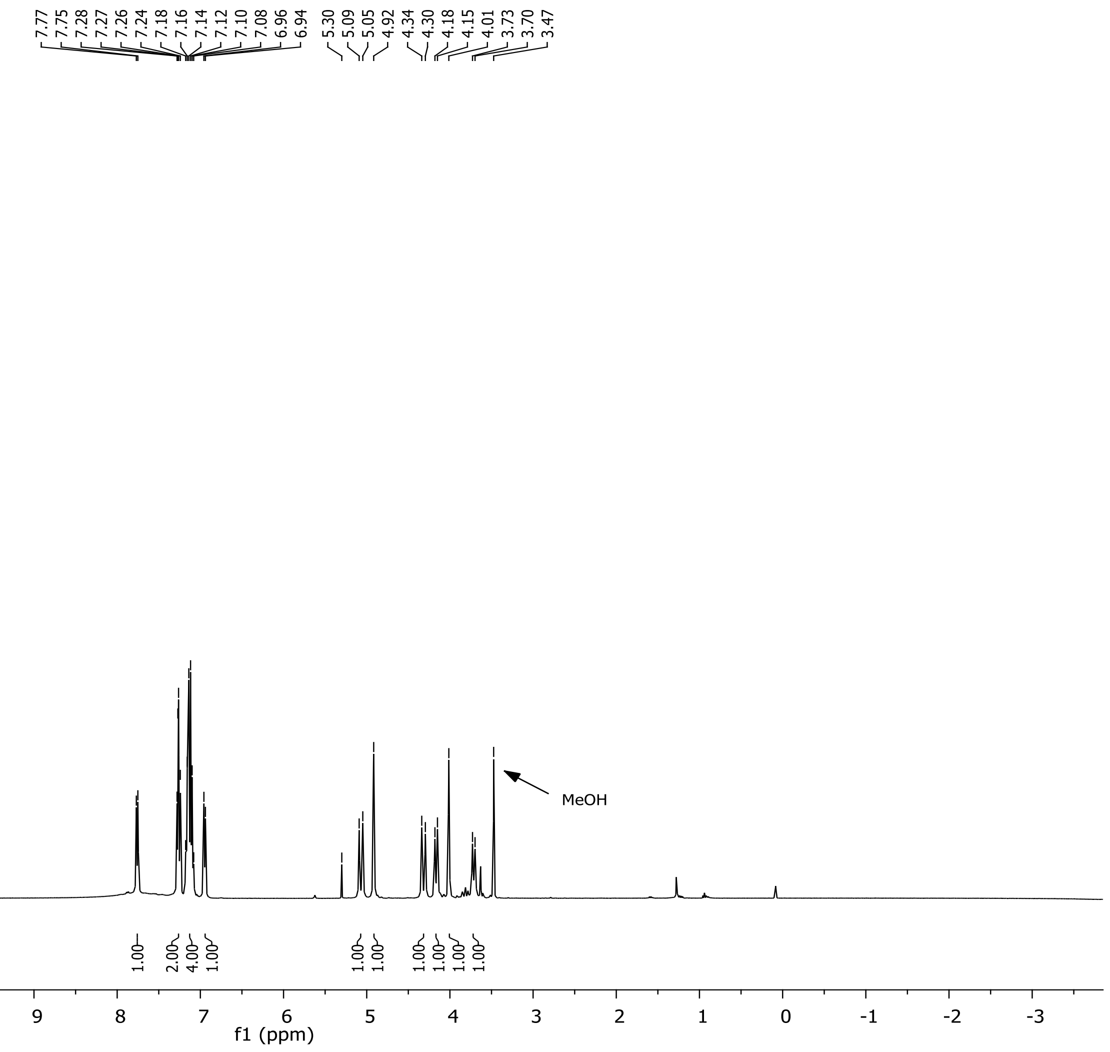

^{13}C NMR of 7,12-dihydro-6,12-methanodibenzo[*c*,*f*]azocine-5-carboxylic acid **(7f)**

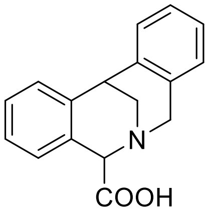

7f

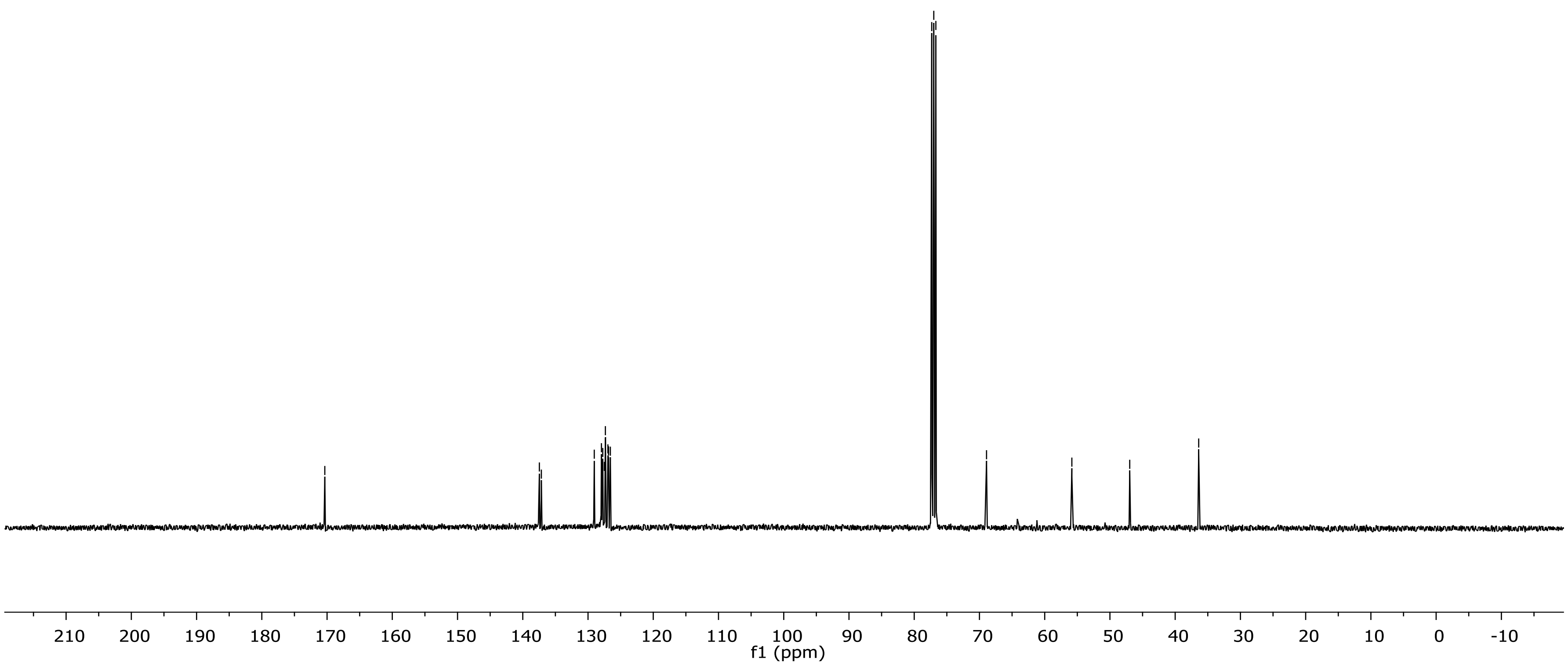

^{1}H NMR of *N*-(2,3-dimethoxybenzyl)-3,4-dimethoxyphenylglycine **(8)**

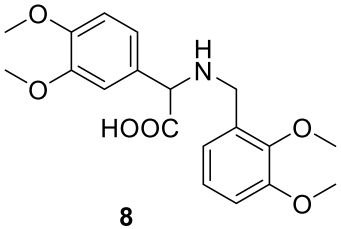

8

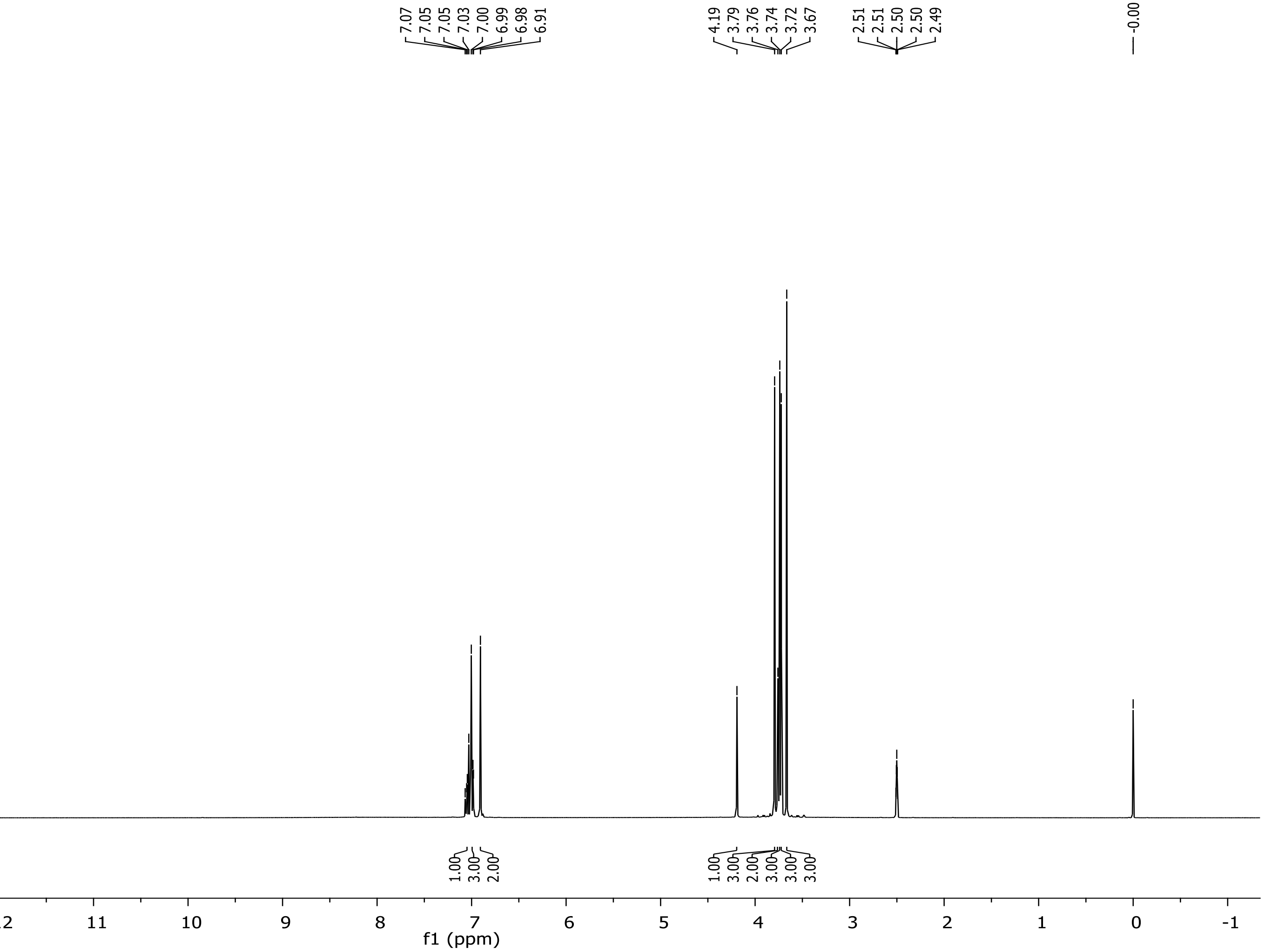

^{13}C NMR of *N*-(2,3-dimethoxybenzyl)-3,4-dimethoxyphenylglycine **(8)**

8

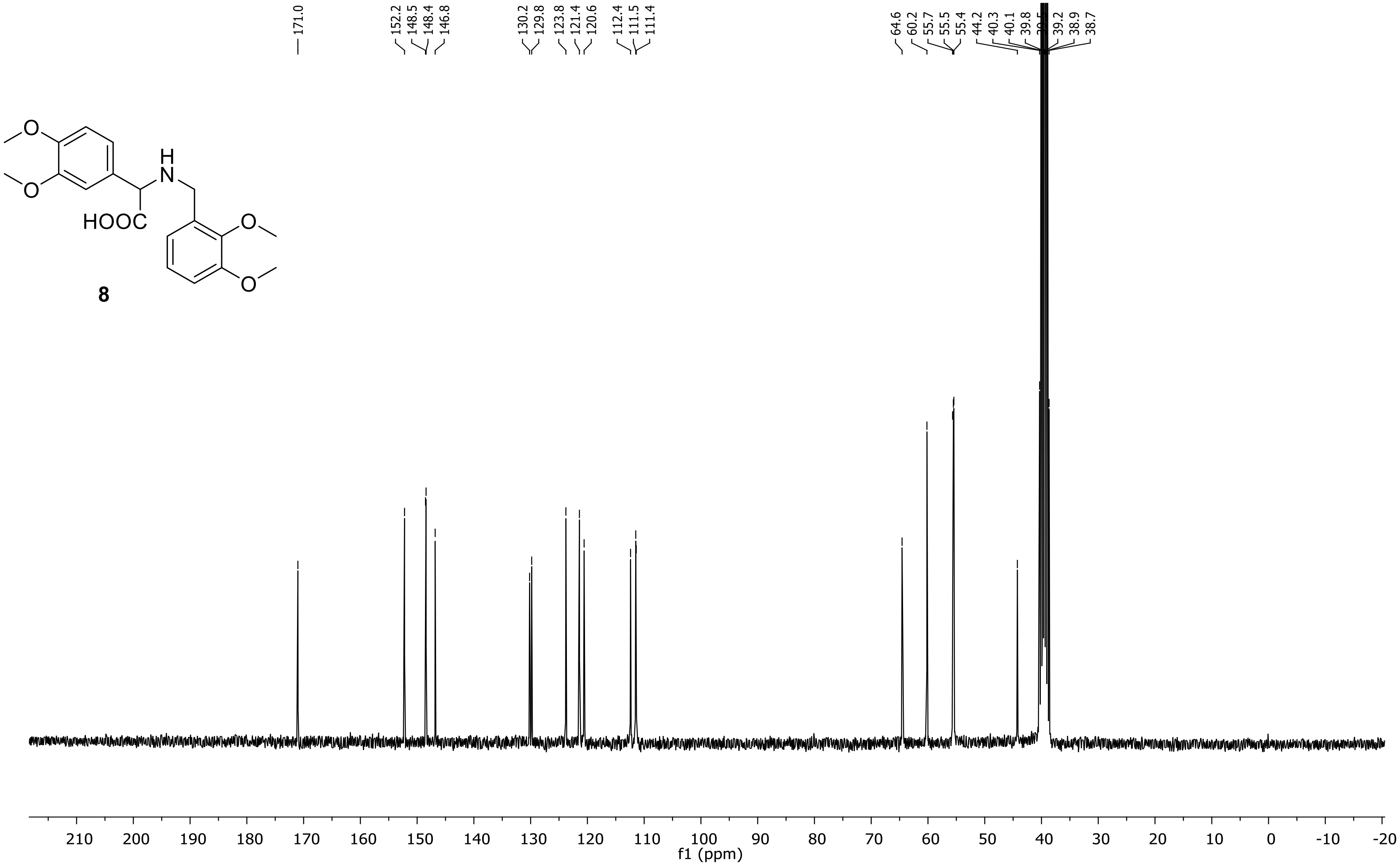

^{1}H NMR of *N*-(2,3-dimethoxybenzyl)-3,4-dimethoxybenzylamine **(10)**

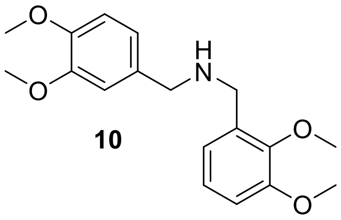

10

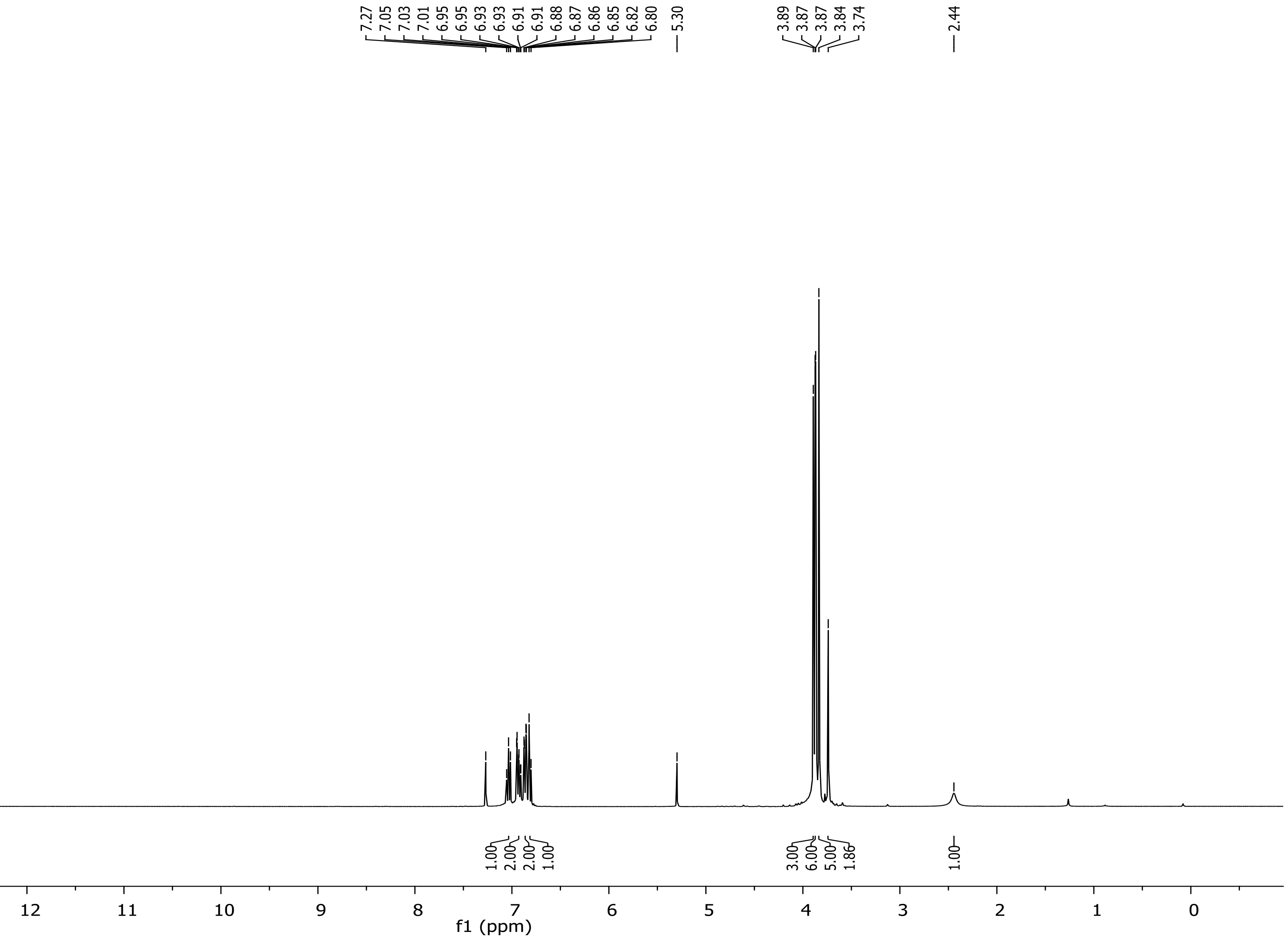

^{13}C NMR of *N*-(2,3-dimethoxybenzyl)-3,4-dimethoxybenzylamine **(10)**

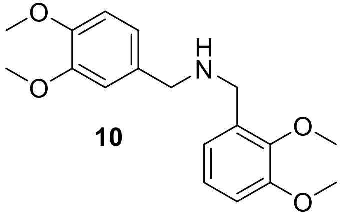

10

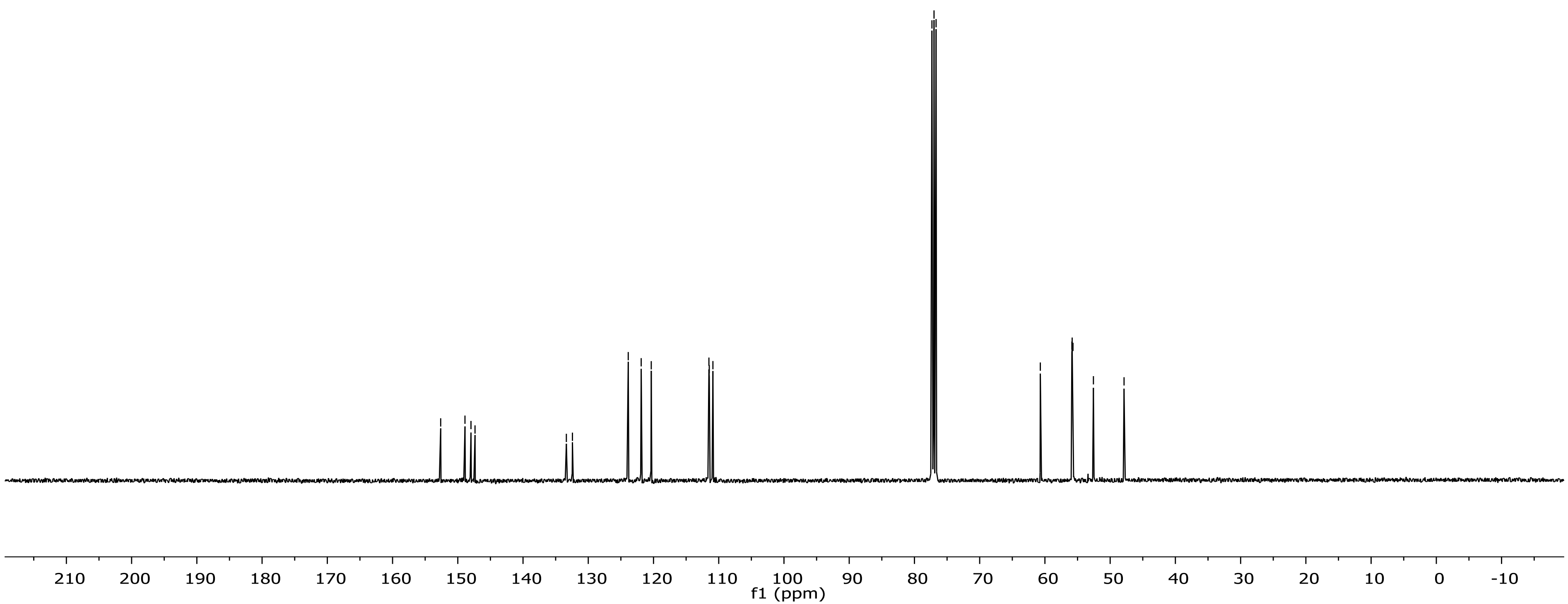

^{1}H NMR of *N*-(2,3-dimethoxybenzyl)-*N*-(3,4-dimethoxybenzyl)aminoacetaldehyde diethyl acetal **(12)**

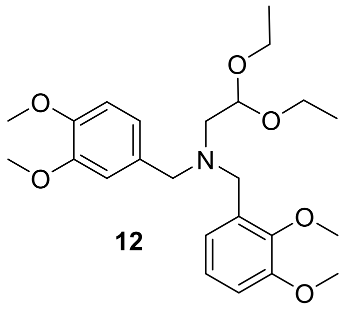

12

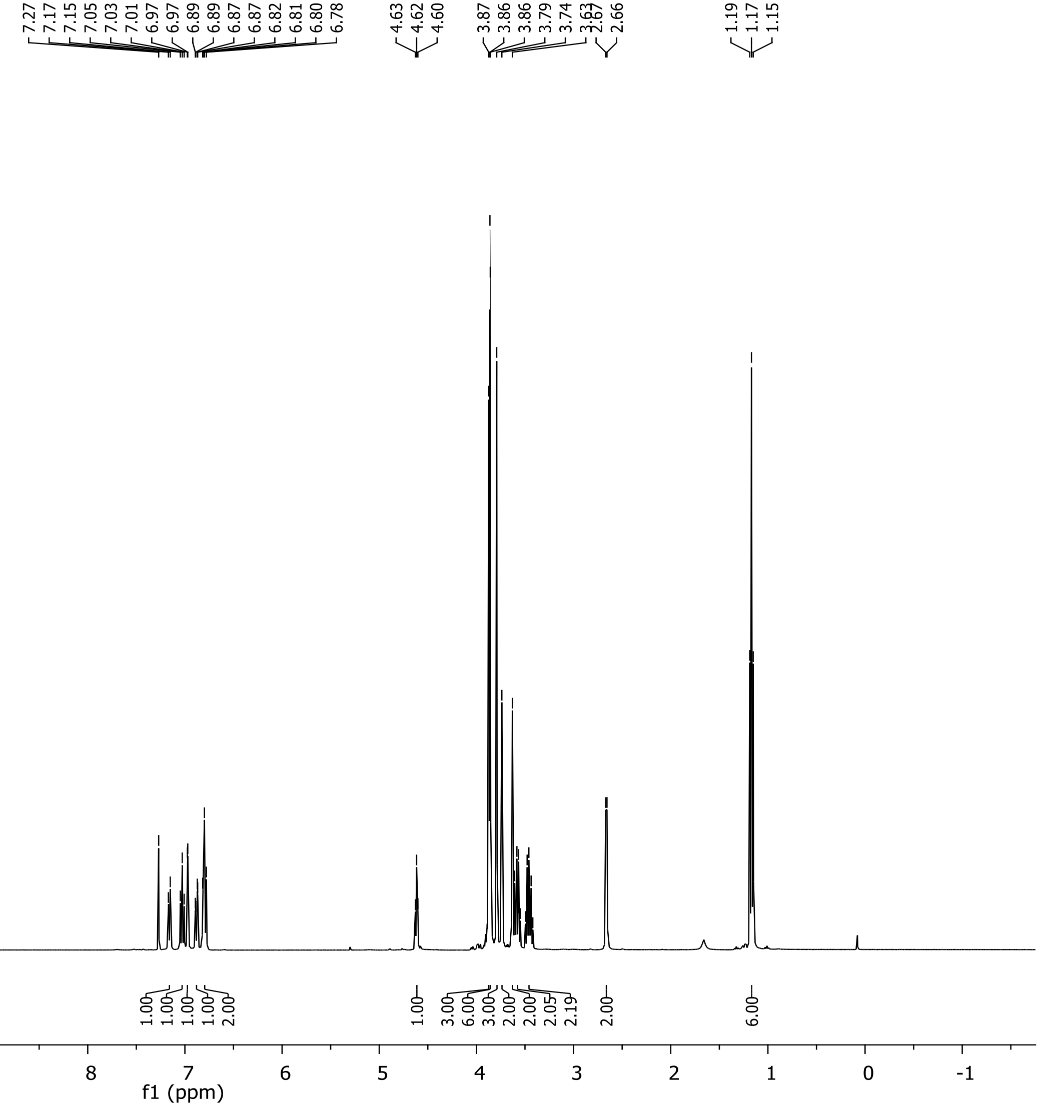

^{13}C NMR of *N*-(2,3-dimethoxybenzyl)-*N*-(3,4-dimethoxybenzyl)aminoacetaldehyde diethyl acetal **(12)**

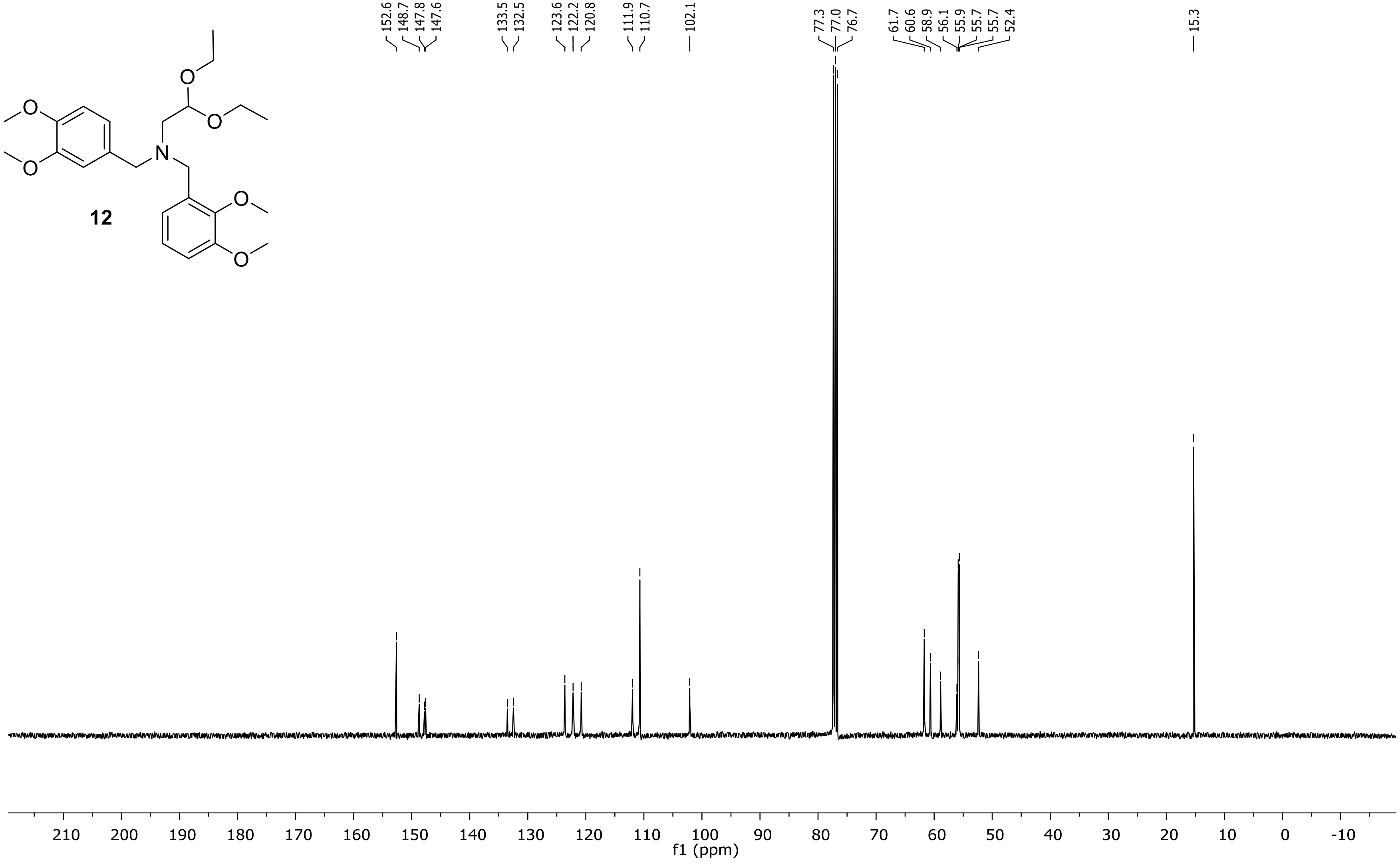

^{1}H NMR of 2,3,8,9-tetramethoxy-7,12-dihydro-6,12-methanodibenzo[*c*,*f*]azocine **(14)**

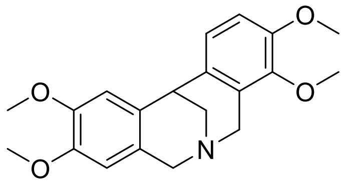

14

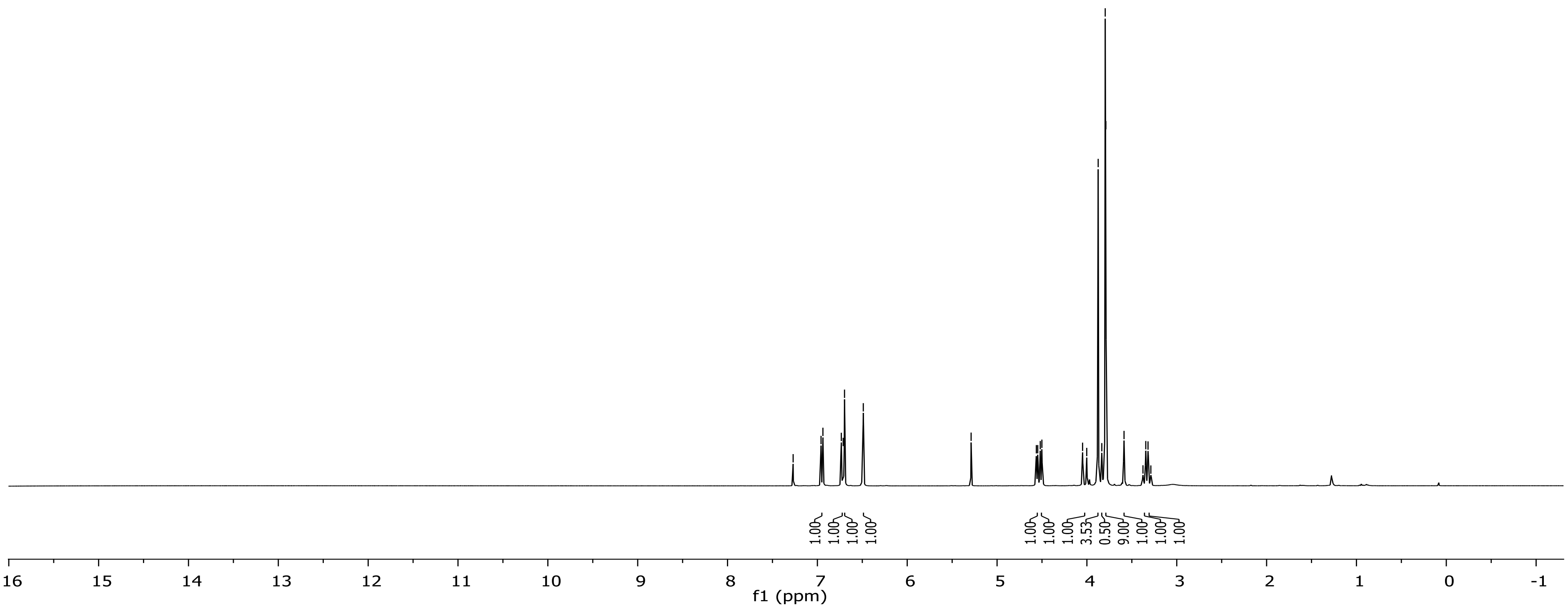

^{13}C NMR of 2,3,8,9-tetramethoxy-7,12-dihydro-6,12-methanodibenzo[*c*,*f*]azocine **(14)**

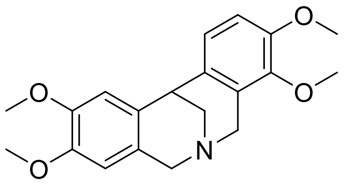

14

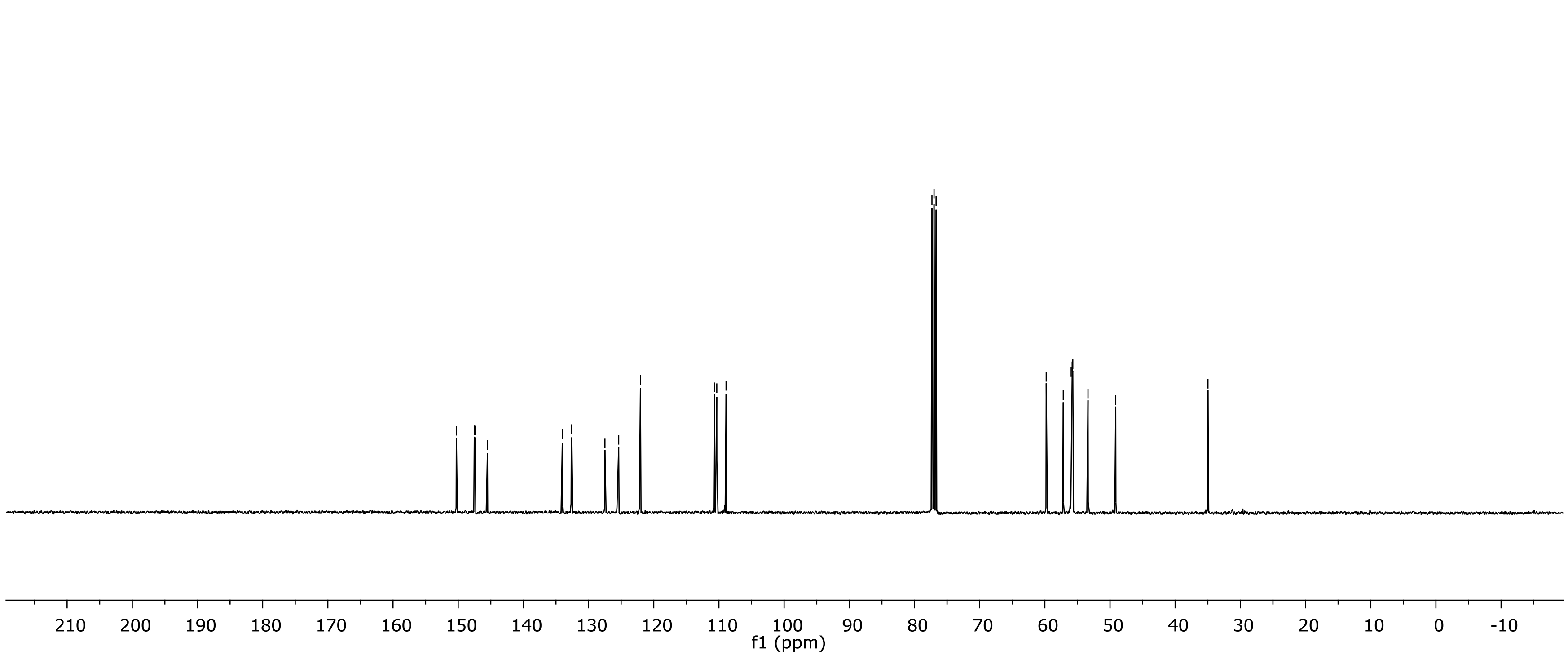

Supplement: File 2 — Copies of 1H NMR and 13C NMR spectra. [file Beilstein_J_Org_Chem-17-2511-s002.pdf]
